# The effect of integrating pain neuroscience education with stabilization exercises using a flexi-bar on functional disability, pain, and geometry of abdominal and multifidus muscles in patients with chronic non-specific low back pain

**DOI:** 10.1371/journal.pone.0355692

**Published:** 2026-08-27

**Authors:** Zahra Dehghani, Sara Abolahrari-Shirazi, Farahnaz Emami, Ghazal Roshdi, Salman Nazary-Moghadam

**Affiliations:** 1 Student Research Committee, Shiraz University of Medical Sciences, Shiraz, Iran; 2 Physical Therapy Department, School of rehabilitation Sciences, Shiraz University of Medical Sciences, Shiraz, Iran; 3 Orthopedic & Rehabilitation Research Center, Shiraz University of Medical Science, Shiraz, Iran; 4 Physical Therapy Department, School of Rehabilitation Sciences, Shiraz University of Medical Science, Shiraz, Iran; 5 Department of Physiotherapy, School of Paramedical and Rehabilitation Sciences, Mashhad University of Medical Sciences, Mashhad, Iran; Nitte (Deemed to be University), Nitte Institute of Physiotherapy (NIPT), INDIA

## Abstract

**Objective:**

Chronic non-specific low back pain is a multifactorial condition encompassing both physical and psychosocial components, frequently characterized by maladaptive pain-related cognitions and fear-avoidance behaviours. Pain neuroscience education seeks to reconceptualize these cognitions and mitigate pain-related fear. This study evaluated the additional benefits of integrating pain neuroscience education with Flexi-Bar-based stabilization exercises on pain intensity, functional disability, and geometry of the abdominal and lumbar Multifidus muscles in individuals with chronic non-specific low back pain.

**Methods:**

Forty-eight participants diagnosed with chronic non-specific low back pain were randomly allocated to one of two groups: (1) Pain neuroscience education followed by Flexi-Bar stabilization exercises or (2) Flexi-Bar stabilization exercises alone. Both groups underwent supervised Flexi-Bar stabilization training sessions three times a week for six weeks. The primary outcome was functional disability assessed by the Roland–Morris Questionnaire at baseline, immediately post-intervention, and at the 4-week follow-up. Secondary outcomes included pain intensity, kinesiophobia, pain catastrophizing, and fear-avoidance beliefs, all measured at the same three time points, as well as ultrasound-based geometry of abdominal and lumbar multifidus muscles, which was assessed at baseline and post-intervention in supine, seated, and standing positions.

**Result:**

Repeated-measures ANOVA revealed significant Time × Group interaction effects, main effects of time, and main effects of Group for both functional disability and pain intensity (p < 0.05). Ultrasound analysis demonstrated significant post-intervention increases in Transverse Abdominis and lumbar Multifidus muscle thickness across all tested positions (p < 0.05), with the pain neuroscience education plus exercise group exhibiting significantly greater increases compared to the exercise-only group.

**Conclusion:**

The combined PNE plus stabilization exercises yielded superior improvements in pain, disability, and muscle geometry compared with stabilization alone. However, the absence of a PNE-only group precludes isolating the educational component’s unique contribution. **Iranian Registry of Clinical Trials; prospectively registered** (Trial number**:** IRCT20230505058095N1).

## Introduction

Low back pain (LBP) is recognized as one of the most prevalent health concerns globally [1]. Estimates suggest a prevalence of 60% −80% among American adults [2] and a lifetime prevalence of approximately 25.2% within the Iranian population [3]. Beyond its health impact, LBP imposes a substantial economic burden, with treatment costs in the United States alone exceeding $87 billion annually [4].

The fear-avoidance model is a widely recognized framework for understanding chronic musculoskeletal pain and has gained considerable attention over the past two decades [5]. According to this model, experiencing painful injury can lead to maladaptive thoughts (e.g., catastrophizing), which result in fear of pain, avoidance behaviors, and, consequently, a reduction in daily activities and a greater functional disability [6]. Avoidance behaviors often persist as they are associated with the expectation of pain occurrence rather than the actual experience of it. Since the individual lacks opportunities to encounter experiences that challenge their beliefs (i.e., engaging with feared activities or situations where pain does not occur), they are unlikely to correct distorted perceptions about the relationship between activities and pain [5].

Reducing fear of movement is a primary aim of Pain Neuroscience Education (PNE). PNE has been identified as an effective approach for improving patient understanding of pain mechanisms and reducing fear, pain, and disability [7]. This approach focuses on educating patients about the mechanisms associated with chronic pain, including central mechanisms of pain and central sensitization, as well as the cognitive-emotional aspects of pain. By providing this knowledge, this educational approach aims to help patients gradually resume the activities they fear [8]. While PNE has demonstrated some effectiveness on its own, its combination with therapeutic exercises and other interventions has shown greater benefits in alleviating symptoms and enhancing patient motivation for rehabilitation [7].

In many patients with chronic LBP, specific musculoskeletal changes have been reported, particularly in key stabilizing muscles such as the Transversus Abdominis (TA) and Multifidus (MF) [9]. These muscles are essential for anticipatory postural adjustment and segmental spinal control. Structural assessments have revealed alterations in the thickness and cross-sectional area (CSA) of these muscles, which may reflect disuse atrophy or compensatory maladaptation. Furthermore, such changes are often accompanied by heightened excitability of the central nervous system (CNS) [10].

Based on previous studies on managing chronic LBP, exercise therapy has strong evidence for reducing pain and disability and facilitating a return to work. Among the various exercise approaches, stabilization exercises have emerged as a promising intervention for reducing pain and disability associated with low back pain [11]. These exercises aim to enhance muscle coordination and minimize unnecessary movements in the lumbopelvic region, thus protecting the trunk against damage and pain [12]. It has been shown that the use of vibration tools such as the Flexi bar in exercises can help to stimulate proprioceptors, improve trunk muscle activation, and promotes neuromuscular control [13]. Furthermore, Flexi-bar exercises specifically target the abdominal and spinal muscles [14]. Moreover, a recent study reported that Flexi-Bar exercise significantly increased erector spinae activation under different standing weight-bearing conditions, supporting its use for targeted spinal muscle activation and re-education [15]

Despite the promising individual effects of PNE and stabilization exercises, there is currently a lack of research on the combined impact of these approaches on pain, functional disability, and muscle geometry in individuals with chronic non-specific LBP. Therefore, this study aims to examine the impact of adding PNE to stabilization exercises using a Flexi-bar on pain, functional disability, and the geometry of abdominal and MF muscles in patients with chronic non-specific LBP. We hypothesize that PNE reduces maladaptive pain-related cognitions and fear-avoidance behaviors, leading to reductions pain intensity, decreased functional disability, and improved exercise adherence. These changes may subsequently enhance neuromuscular control during Flexi-Bar stabilization training, potentially resulting in greater improvements in abdominal and lumbar Multifidus muscle geometry compared to stabilization exercises alone.

## Materials and methods

### Study design

This randomized clinical study was conducted at the School of Rehabilitation Sciences, Shiraz University of Medical Sciences, Shiraz, Iran. **Participant enrollment** began on 1 July 2023 and was completed when the last participant was recruited on 28 January 2024. The **6-week intervention period** for the last enrolled participant was completed on 10 March 2024, and the **final 4-week follow-up assessment** for this participant was completed on 10 April 2024. The trial was registered in the Iranian Registry of Clinical Trials (registration number: IRCT20230505058095N1) which is an approved WHO primary registry.

The present study employed a two-arm, parallel-group randomized controlled trial design. Participants were randomly allocated to either an experimental group receiving PNE plus Flexi-Bar stabilization exercises, or a control group receiving Flexi-Bar exercises alone.

### Participants

Forty-eight patients with chronic non-specific LBP were randomly divided into two groups.

**Fig 1:**
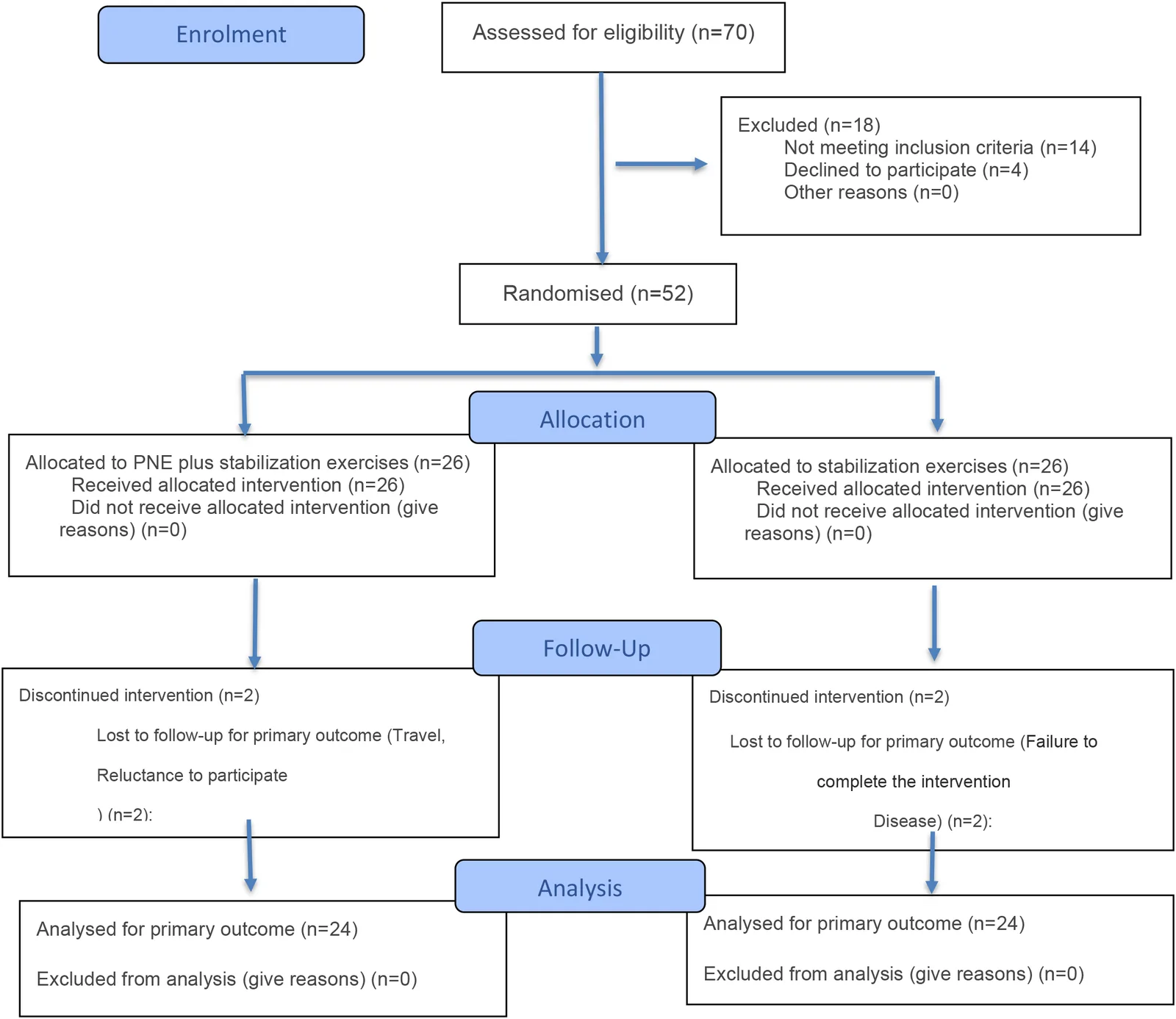
Flow diagram of participant recruitment and allocation. Created by the authors. Published under CC BY 4.0.

All patients were informed about the study’s conditions, and informed consent was obtained from each participant. Participants were screened for eligibility, and those who met the inclusion criteria were randomly allocated to the experimental or control group (Fig 1). The primary outcome was functional disability, assessed using the Roland‑Morris Questionnaire (RMQ). The secondary outcomes were pain intensity, fear‑avoidance beliefs, pain catastrophizing, and kinesiophobia. Ultrasound measures of muscle thickness and cross‑sectional area served as physiological secondary outcomes.The LBP patients were included in the present study if they met the following criteria: aged between 20 and 50 years, reported LBP for at least three months, scored 3–7 on the Visual Analogue Scale (VAS), held at least a high school diploma, and scored over 8 on the RMQ [16]. Exclusion criteria included a history of lumbar spine malignancy or metastasis [17], acute infections [17], inflammatory or systemic diseases [18], structural spinal disorders [19], neurological conditions or brain injury [18], prior lower limb, abdominal or lumbar surgeries [20], pregnancy [21], recent physiotherapy or sports participation within the last three months [22], and musculoskeletal or orthopedics disorders of the limbs [23]. In addition, relevant tests were administered to confirm the diagnosis of non-specific LBP.

### Ethics statement

All participants provided written informed consent prior to enrollment. The study protocol was approved by the Ethics Committee of Shiraz University of Medical Sciences (Ethics Code: IR.SUMS.REHAB.REC.1402.001). The full trial protocol is available (Supporting Information, S3 File and S4 File). We confirm that we have obtained permission from the study sponsor to publish the full trial protocol under CC BY 4.0; the formal permission letter is available in Supporting Information

### Randomization

After the baseline assessment, patients were randomly allocated into two groups. A balanced block randomization method was used to allocate participants into two groups. Each block contained four participants, and block randomization was performed within these blocks. To achieve equal distribution, six possible arrangements of the four blocks were created. Within each block, two participants were assigned to the experimental group and two to the control group. The allocation sequence was generated prior to the study using the website www.Randomization.com, with the letter A designating the experimental group and the letter B the control group. This approach ensured that the groups were comparable and that any differences observed between them were due to the intervention and not random allocation. To conceal the allocation sequence, sealed opaque envelopes were prepared. The randomization sequence was printed on separate sheets and placed in opaque envelopes. A research assistant, blinded to baseline assessment findings, opened the sealed, opaque envelopes to reveal group allocation prior to starting the intervention.

### Blinding

The assessor was blinded to group allocation during data collection. Patients were instructed not to disclose any information regarding group allocation to the assessor conducting the assessments. An expert physiotherapist evaluated the patients pre-treatment, post-treatment, and at follow-up.

### Measurements

**Fig 2:**
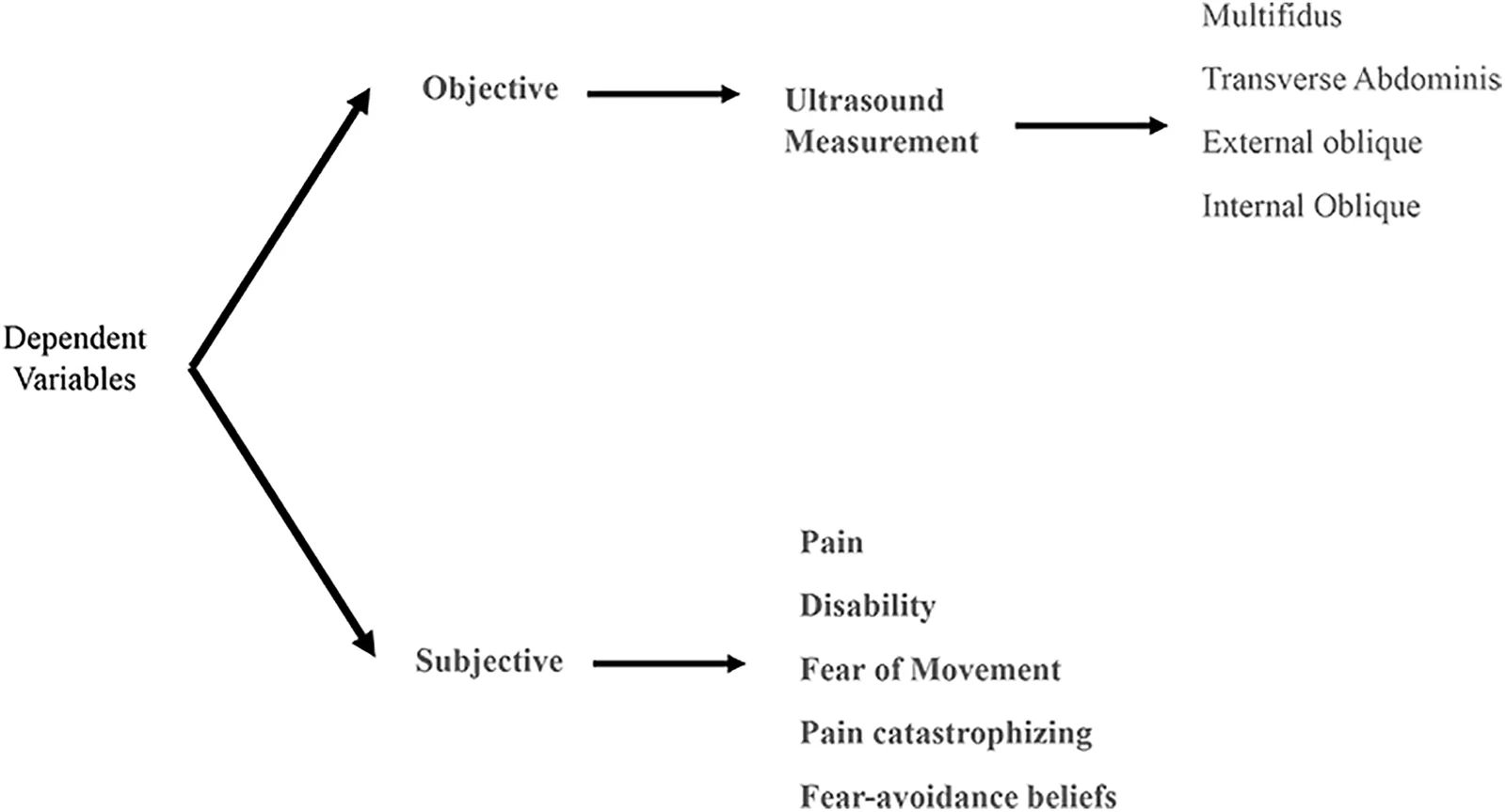
Variables of the study. Created by the authors. Published under CC BY 4.0.

All variables were measured at baseline, post-intervention, and follow-up, except for ultrasonographic measures, which were collected only at baseline and post-intervention (Fig 2).

#### Primary outcome.

**Disability**: Functional disability was the primary outcome, assessed using the RMQ. This variable was designated as the primary endpoint because the sample size calculation was based on the expected change in RMQ scores reported by Bagheri et al. The RMQ is most sensitive for patients with mild to moderate disability due to low back pain, with a 24-item scale (where 0 = no disability and 24 = maximum disability). The RMQ does not categorize the severity of functional disability in specific levels (for example, 40%–60% of disability is severe).The Persian version of the RMQ has an ICC of 0.83 [24]. The minimal clinically important difference (MCID) for the RMQ is 2.5 points [25].

#### Secondary outcomes.

**Pain:** Pain intensity was measured using a VAS ranging from 0 (no pain) to 10 (highest pain level), a valid and reliable tool with an intra-class correlation coefficient (ICC) of 0.91 [26]. The MCID for the VAS is 2 points [27].

**Fear of movement:** The Tampa Scale of Kinesiophobia)TSK(is a self-reported questionnaire that uses a 4-point Likert scale with Activity Avoidance and Somatic Focus subscales. The 17item TSK total scores range from 17 to 68 where the lowest 17 means no or negligible kinesiophobia, and the higher scores indicate an increasing degree of kinesiophobia. The Persian version of TSK demonstrated good reliability, with an ICC of 0.86 [28]. Additionally, the MCID for this Questionnaire is 6 points [29].

**Pain catastrophizing:** The Pain Catastrophizing Scale (PCS) is a 13-item self-report questionnaire assessing participants’ pain-related thoughts and feelings, scored 0–4 for each item. It yields a total score (0–52) and three subscales: rumination, magnification and helplessness. Higher score indicates greater level of catastrophizing. The Persian version demonstrated good reliability (ICC = 0.71–0.81) [30]. The MCID for this Questionnaire is 9.1 points [31].

**Fear-avoidance beliefs:** Fear-avoidance beliefs were evaluated using the FABQ, which comprises 16 items across two domains-physical activity and work. Participants rate each item on a 7-point Likert scale (0 = completely disagree to 6 = completely agree), yielding a total score of 0–96, with higher score indicating stronger fear avoidance beliefs. The Persian version showed good test-retest reliability (ICC = 0.80) and the MCID of 11 points [32,33].

**Ultrasound Measurement:** Ultrasound assessments of the thickness and CSA of the abdominal and MF muscles were conducted before and after the six-week intervention using a 7.5 MHz linear -array probe and B-mode ultrasound (HS-2600, Honda, Japan) (Fig 2). The reliability of ultrasound measurements for muscle thickness was well established in previous studies, with intra-rater ICCs ranging from 0.97 to 0.99 for the transversus abdominis and 0.92 to 0.98 for the lumbar multifidus [34]. All imaging in this study was performed by a single experienced physiotherapist to maximize consistency [35].

#### Transversus Abdominis (TA), Internal Oblique (IO) and External Oblique (EO).

**Fig 3:**
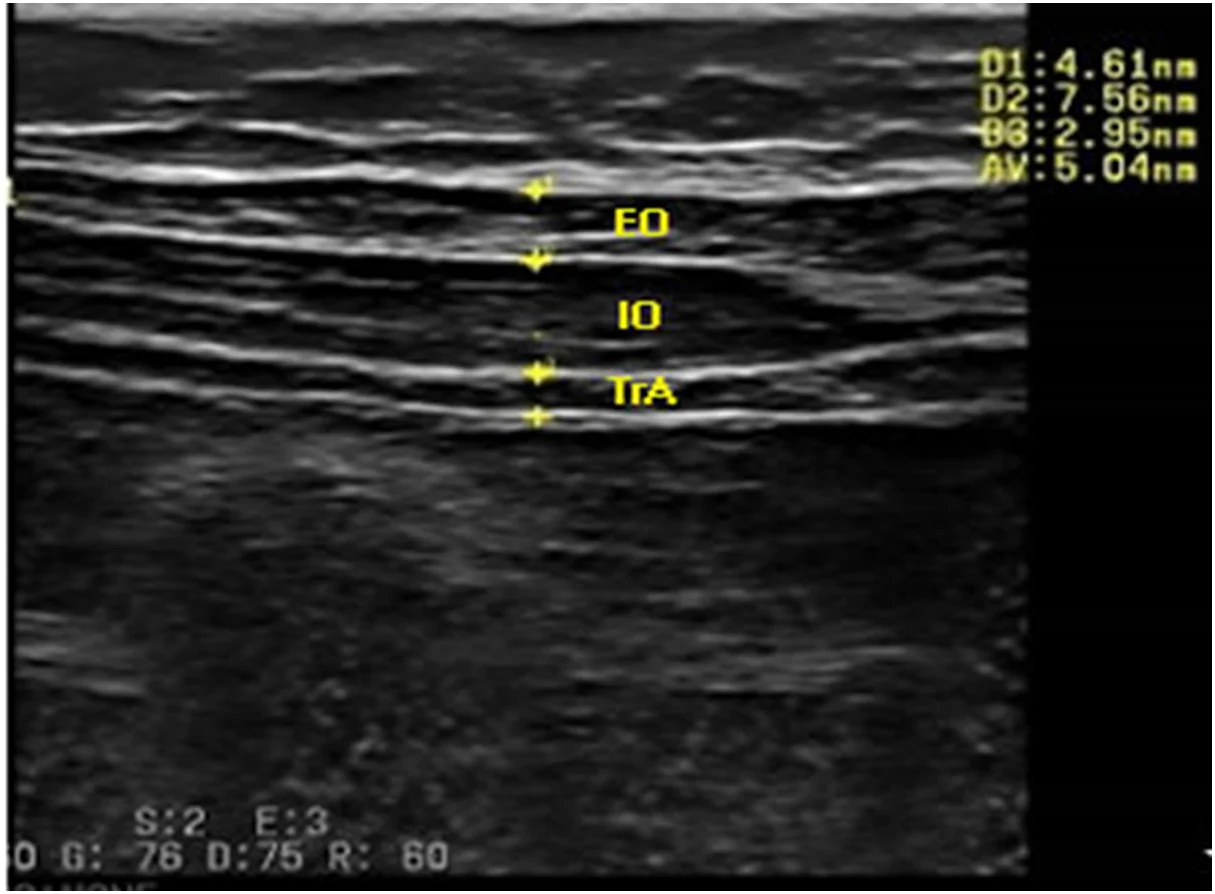
Ultrasound image of abdominal muscles at rest. Created by the authors. Published under CC BY 4.0.

**Fig 4:**
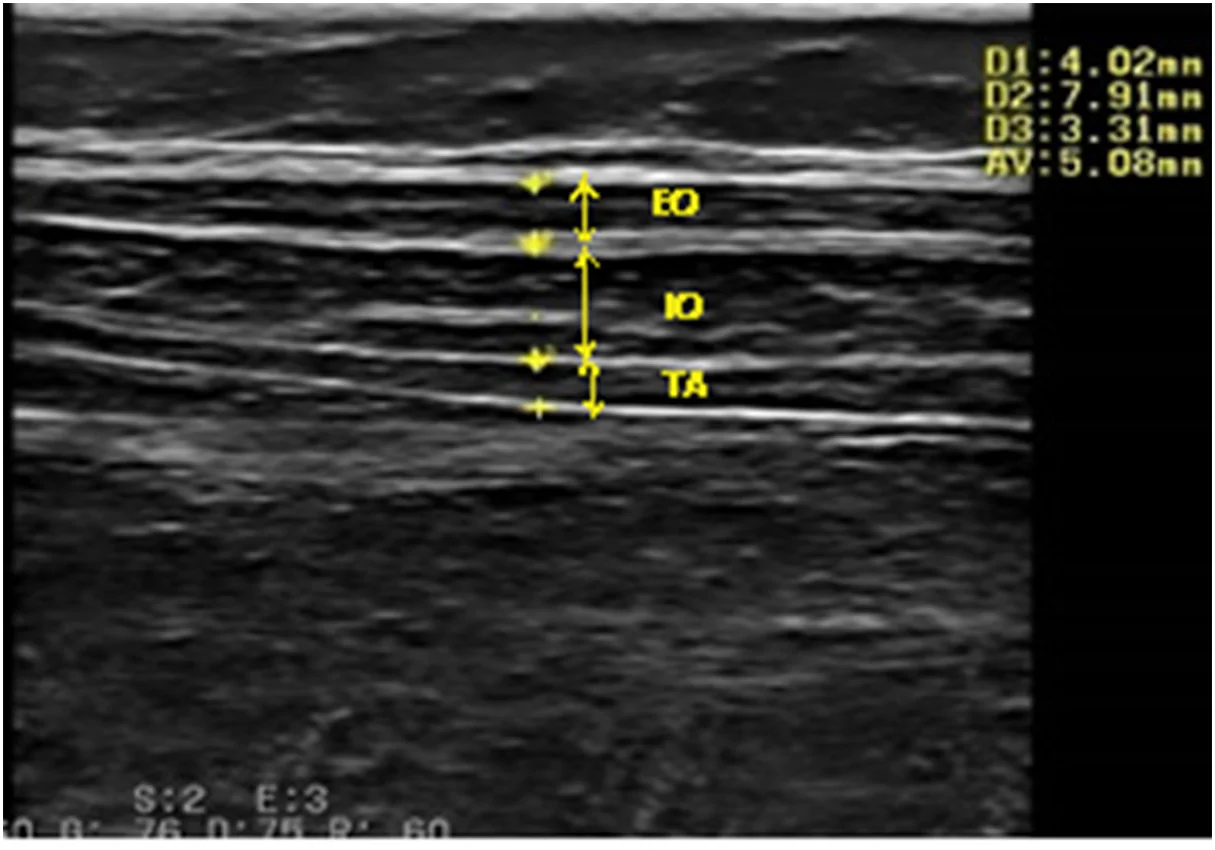
Ultrasound image of abdominal muscles in contracted state. Created by the authors. Published under CC BY 4.0.

To measure TA, IO and EO muscle thickness, participants lay supine with knees flexed at 90 ° and hips flexed at 60 °. The transducer was positioned transversely at an oblique angle between the anterior superior iliac spine (ASIS) and the midpoint of the last rib along the anterior axillary line [35]. Three images from both sides were recorded in a resting state at the end of exhalation, with a 30-second interval between each capture (Fig 3). Additionally, three images are captured from both sides during the contraction condition while participants performed the Abdominal Drawing-In Maneuver (ADIM), with a 30-second interval between each image, all at the end of exhalation (Fig 4). A Stabilizer Pressure Biofeedback Unit (PBU, Chattanooga Group, Hixson, TN, USA) was placed between the firm mattress and the participant’s lumbar region in a crook-lying position, and the bulb was inflated to a baseline pressure of 40 mmHg [36]. Participants were instructed to gradually draw in their lower abdominal muscles and to maintain the contraction for seven seconds. Participants were specifically instructed to contract their lower abdominal muscles—focusing on the TA and IO—at approximately 30% of maximum voluntary isometric contraction (MVC), as determined using pressure biofeedback calibration during pilot testing [37]. This submaximal intensity was selected to minimize undesired co-contraction of superficial musculature (e.g., Rectus Abdominis) [38]. A controlled contraction–relaxation sequence was implemented to prevent premature fatigue and to ensure methodological consistency across participants. Pressure levels corresponding to 30% MVC were maintained using the calibrated handheld biofeedback unit. During the execution of the ADIM, the pressure recorded by the PBU was increased by 0–2 mmHg. At the end of expiration, clear images of the deep abdominal layers were captured for subsequent analysis [39,40].

### Multifidus

**Fig 5:**
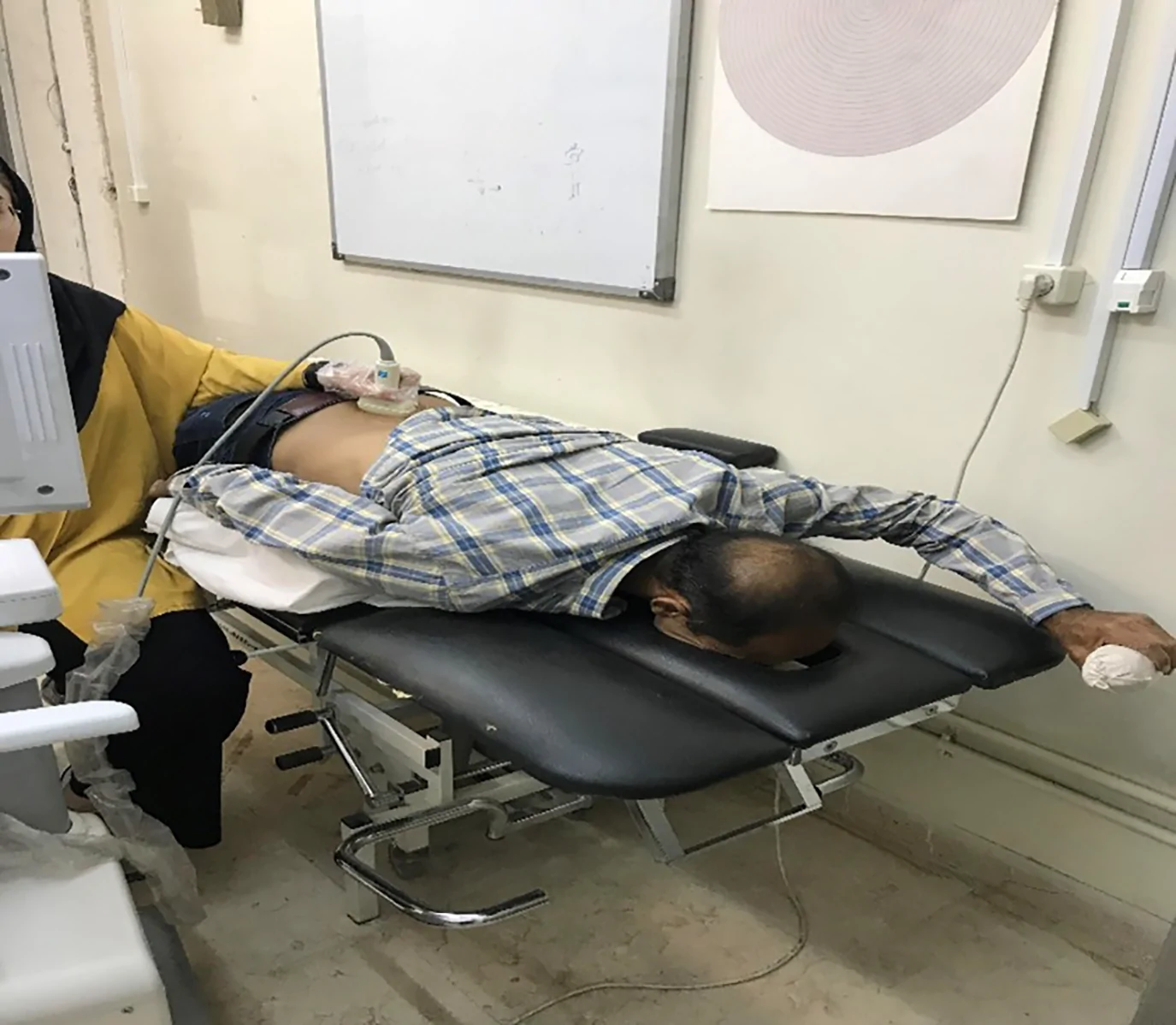
Ultrasound image showing of the thickness of the Multifidus muscle in the prone position during contraction. Created by the authors. Published under CC BY 4.0.

**Fig 6:**
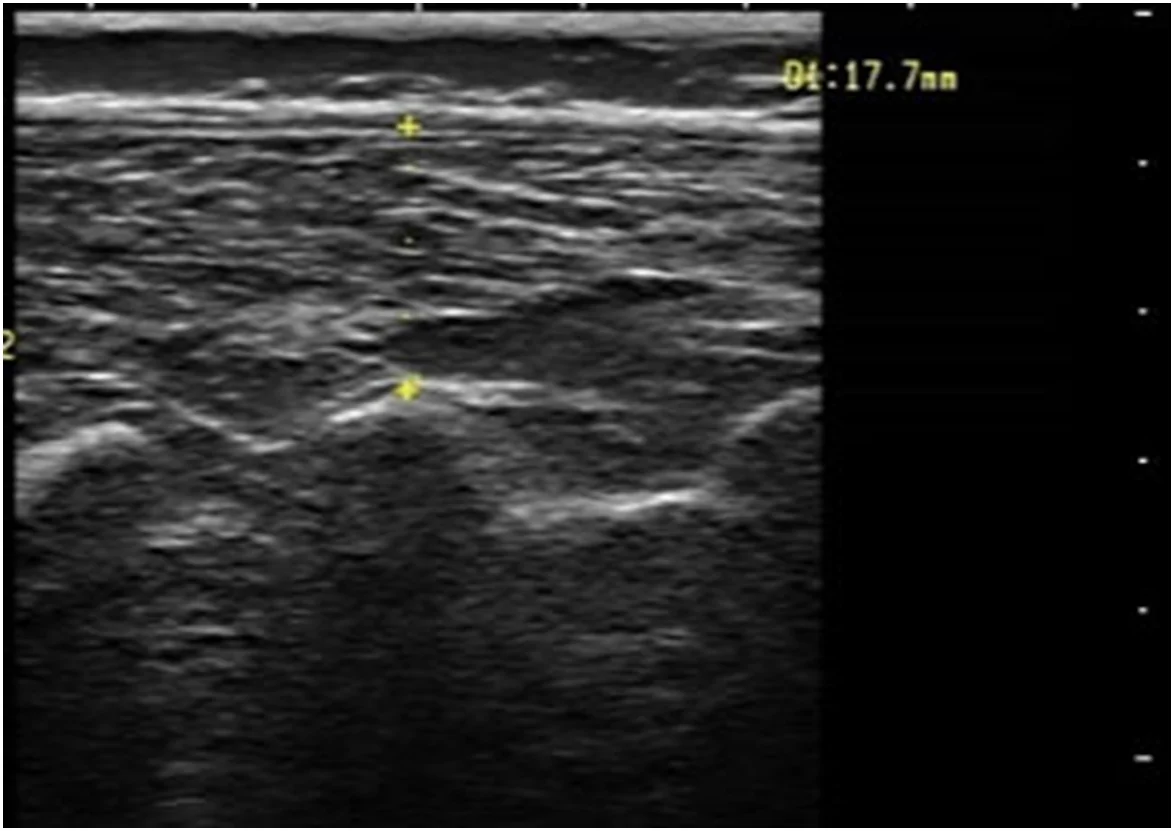
Ultrasound image of the thickness of the Multifidus muscle at rest. Created by the authors. Published under CC BY 4.0.

**Fig 7:**
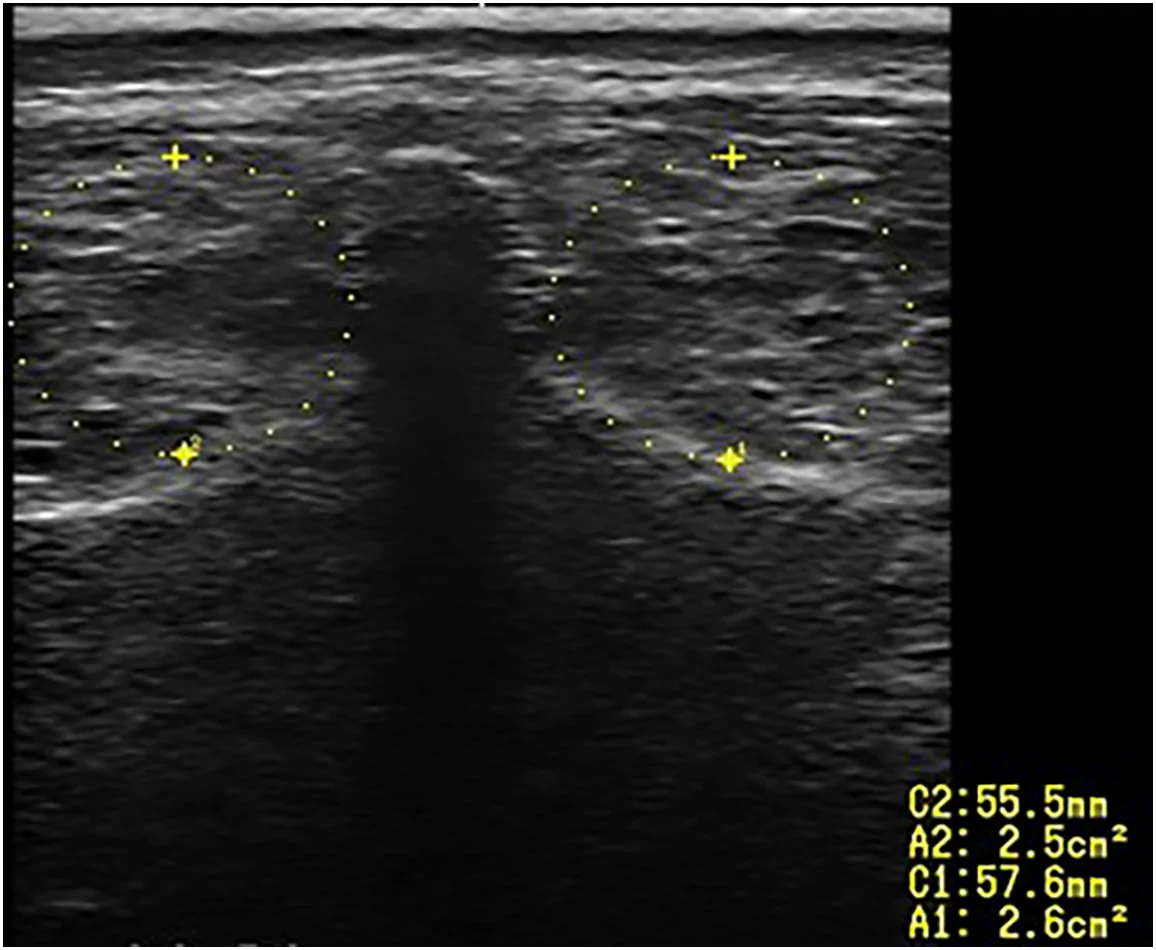
Ultrasound image of the cross-section of the Multifidus muscle. Created by the authors. Published under CC BY 4.0.

**Fig 8:**
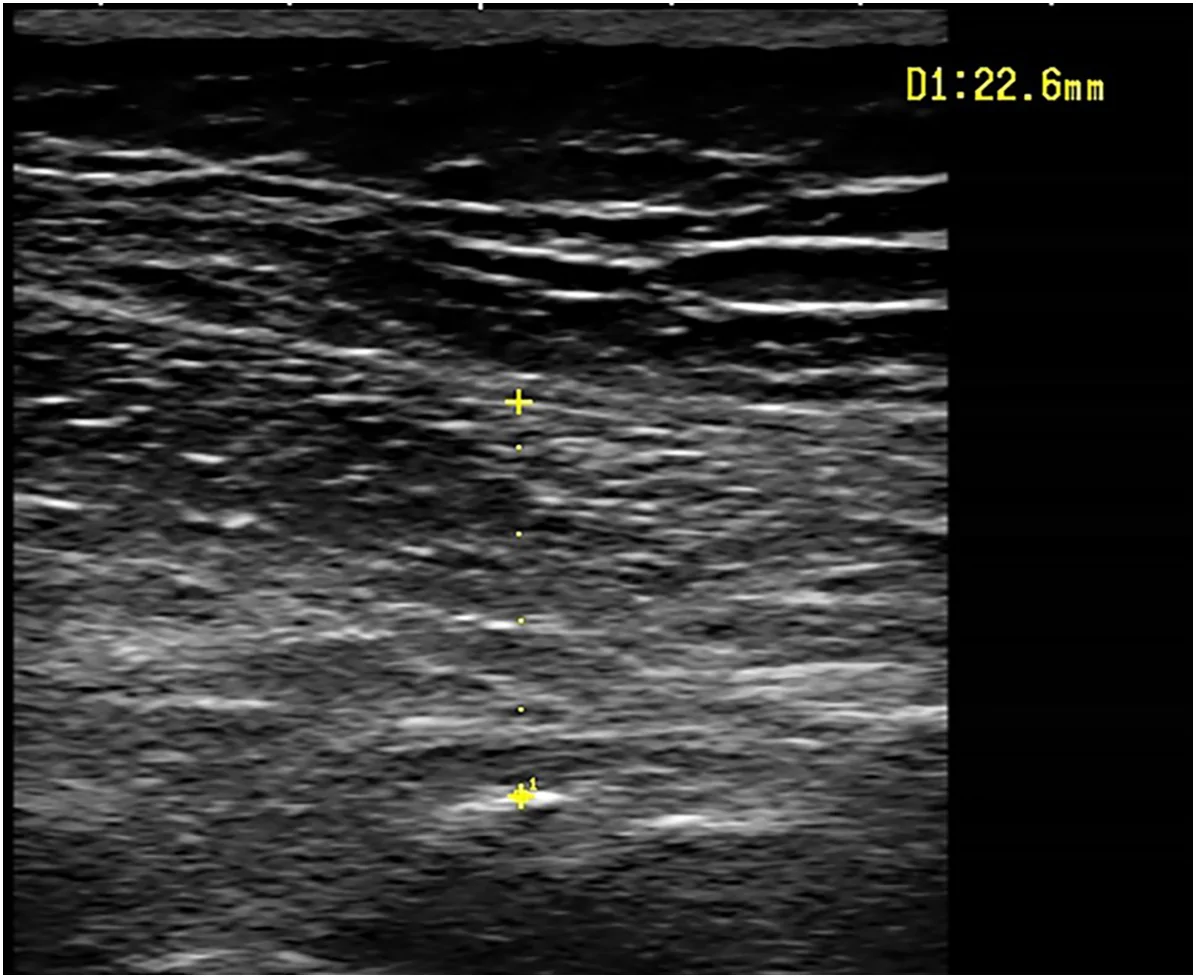
Ultrasound image of the thickness of the Multifidus muscle during contraction. Created by the authors. Published under CC BY 4.0.

Multifidus muscle thickness and CSA were measured with participants lying prone, arms positioned alongside the body, head placed in a face hole, and abdomen supported by a pillow to minimize lumbar lordosis [41]. To measure the thickness of the MF muscle on both side at the L4-L5 level, the spinous process of the fifth lumbar vertebra served as the reference point. The transducer is then placed longitudinally, just lateral to the L4 spinous process, and was slightly rotated in both the cephalocaudal and mediolateral directions to create a small angle (approximately 10 °) with the midline of the body. This positioning helped to identify the zygapophyseal joint between the fourth and fifth lumbar vertebrae and allowed the MF muscle to be recorded in the resting state, with three measurements taken on each side [42] (Fig 5). The thickness of the MF muscle was measured from the muscle fascia located beneath the subcutaneous tissue to the echogenic shadow of the lamina. CSA was measured by placing the transducer transversely on the fourth lumbar spinous process to record MF images bilaterally and simultaneously (Fig 6). In cases where this was not possible, measurements were taken separately from each side. This procedure resulted in three recordings per side for both the resting and contracting states. To measure the resting-state CSA of the MF muscle, the echogenic shadow of the lamina of the fourth lumbar vertebra was used as the deepest boundary, the thoracolumbar fascia served as the superior boundary, the fascia separating the MF muscle from the longissimus was considered the lateral boundary, and the acoustic shadow of the spinous process was regarded as the medial margin [41,43] (Fig 7). For MF muscle contraction, weight was added to the opposite hand, according to individual body weight: 0.68 kg for participants< 68.2 kg, 0.9 kg for those between 68.2 and 90.9 kg, and 1.36 for those > 90.9 kg. The weight was lifted 5 cm from the bed with shoulder abduction at 120 ° and elbow flexion at 90 ° [43]. The amount of load introduced by the weight to the MF muscle was about 30% of the MVC. Each contraction was held for three seconds at the end of exhalation and was repeated three times per side [43] (Fig 8). To assess activation of the deep stabilizing muscle (MF) in the prone, sitting, and standing positions, a standardized resistance load was applied through the opposite arm to induce a controlled rotational torque across the lumbar spine. The resistance weight was calibrated to approximately 30% of each participant’s MVC, determined relative to body mass index (BMI) and overall physical capacity during initial screening. This load stratification approach was adopted from validated lumbopelvic stabilization research protocols [35,44], ensuring uniform mechanical loading across all participants despite differences in body composition and physical capability. In addition to capturing images in the supine position, all ultrasound evaluations were conducted in both sitting and standing positions, with a 5-minute rest interval between each evaluation [35].

Ultrasound imaging procedures were performed across three postural states—supine (minimal gravitational load), sitting (intermediate load), and standing (maximal load)—to assess the effect of posture on muscle geometry, specifically thickness and CSA. Each assessment session included three independent, non-fatiguing repetitions per side for each targeted muscles (TA, IO, EO, MF). Between postural assessments, participants maintained a standardized rest period of five minutes, timed with a stopwatch, to minimize residual muscle fatigue that could affect the accuracy of fascicle-length or thickness measurements.

**Fig 9:**
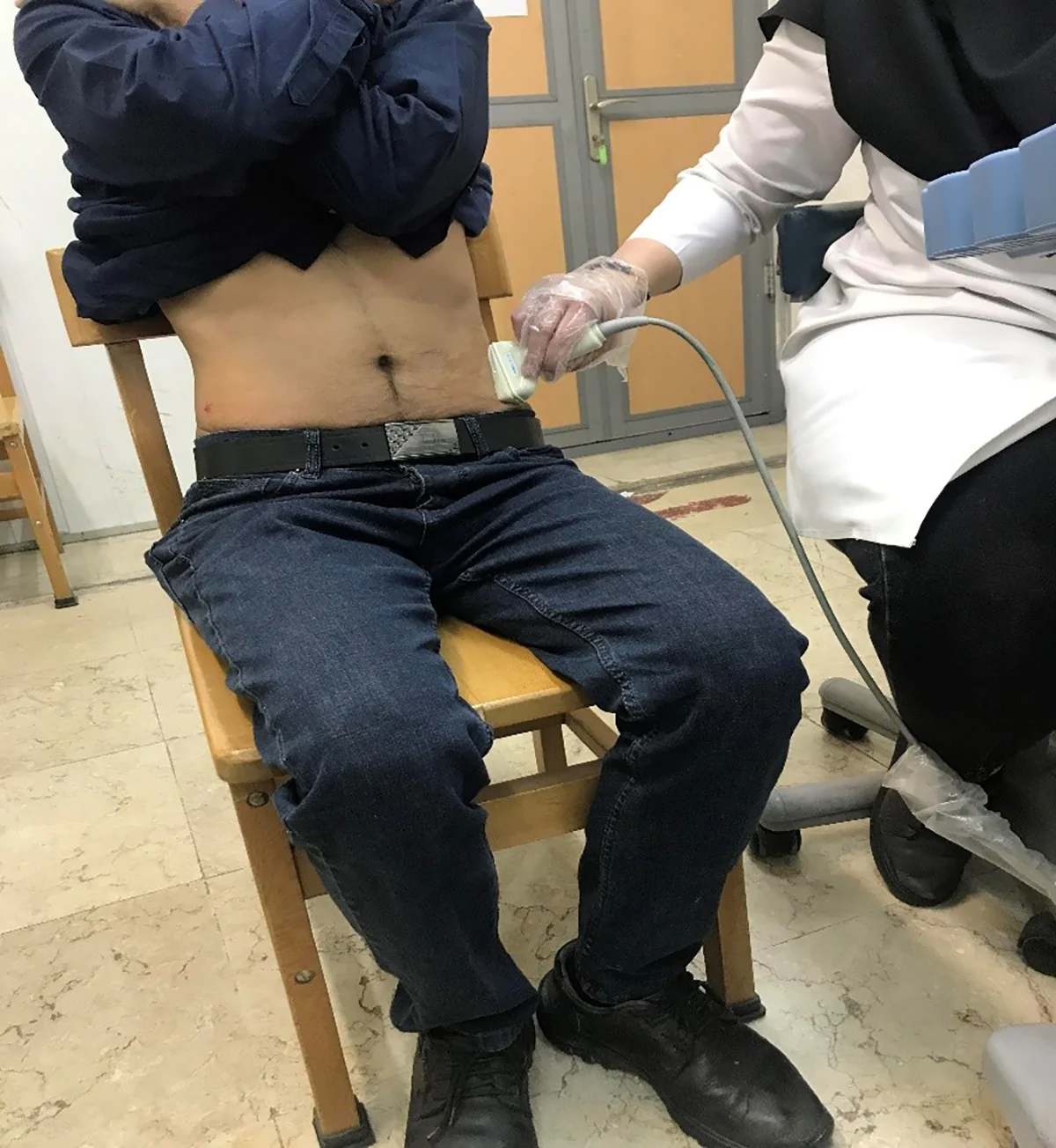
Ultrasound image showing abdominal muscle thickness in the sitting position. Created by the authors. Published under CC BY 4.0.

**Fig 10:**
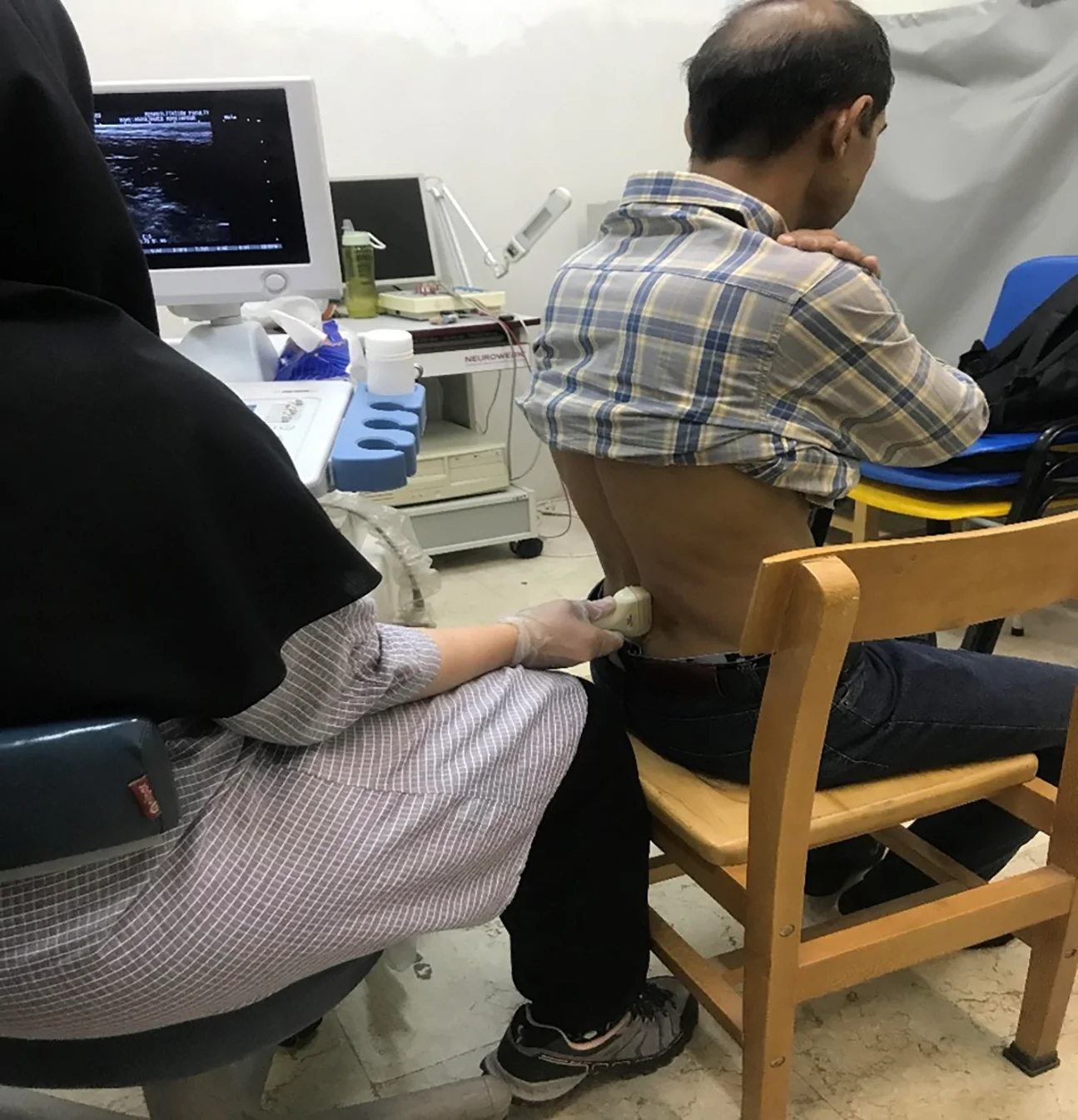
Ultrasound image showing Multifidus muscle thickness in the sitting position. Created by the authors. Published under CC BY 4.0.

For sitting measurements, participants sat on a 43 cm-high, handle-free chair in a neutral-spine position, with hands resting on opposite shoulders and feet flat on the floor, hips and knees flexed at 90 ° [9]. Similar to the prone measurement, bilateral resting-state assessments of the abdominal and MF muscles were performed (Fig 9 and 10). ADIM was performed for abdominal muscle contraction [44]. Contraction of MF muscle was elicited by holding a weight with the contralateral (opposite) hand to minimize compensatory activation of ipsilateral trunk muscles. During this task, the shoulder was flexed at 90°, and the elbow fully extended. The selected weights (0.68–1.36 kg) corresponded to approximately 30% of maximal voluntary isometric contraction (MVIC) and remained consistent across sessions for each participant. Each contraction was held for three seconds at the end of exhalation and recorded three times per side [45,46].

**Fig 11:**
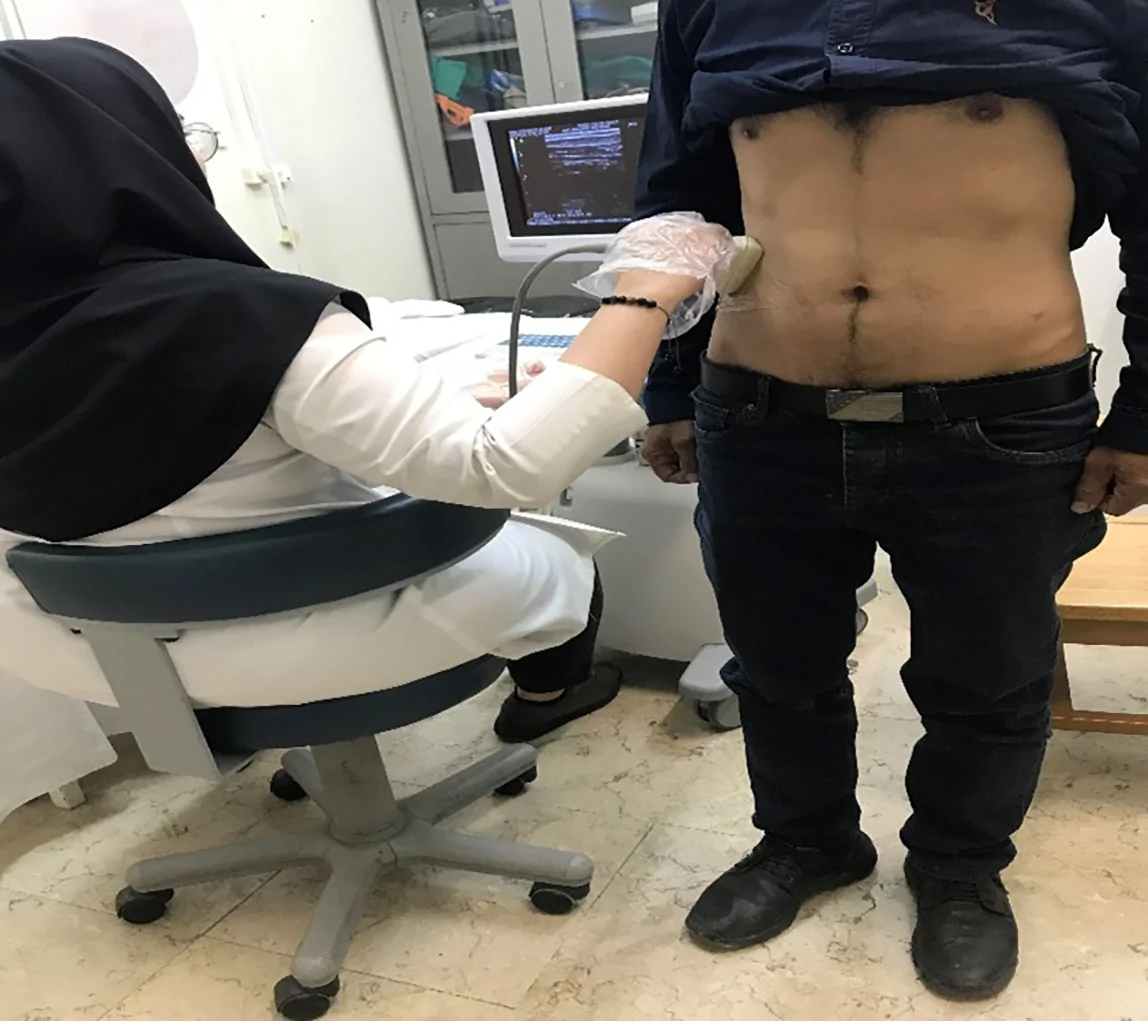
Ultrasound image showing abdominal muscle thickness in the standing position. Created by the authors. Published under CC BY 4.0.

**Fig 12:**
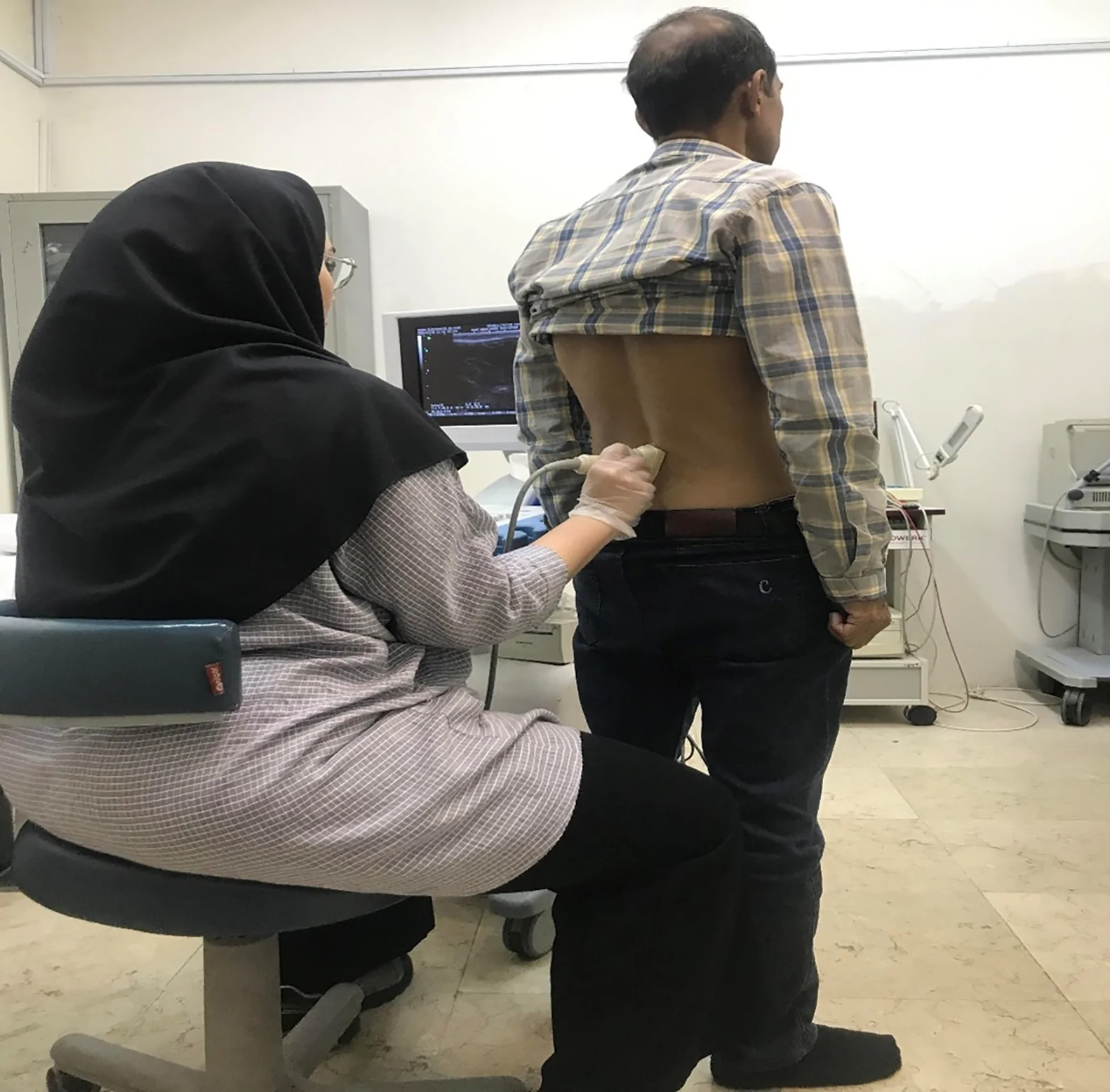
Ultrasound image showing the Multifidus muscle thickness in the standing position. Created by the authors. Published under CC BY 4.0.

Standing-position images were acquired participants standing upright, hands relaxed beside the body, and eyes directed forward. To ensure a natural standing posture, participants were asked to take a few steps in place for several seconds and then stop at the same point [43]. As in the prone position, resting-state measurements of the abdominal and MF muscles were performed (Fig 11 and 12). ADIM was used to provoke abdominal contraction [44]. Contraction of MF muscle was induced by holding a weight with the contralateral (opposite) hand to minimize compensatory activation of ipsilateral trunk muscles. The shoulder was flexed at 90°, and the elbow fully extended while maintaining the hold for three seconds. The selected weights (0.68–1.36 kg) represented approximately 30% of MVIC and were consistent across sessions for each individual [43]. The selection of each position for evaluation was randomized. All ultrasound images were analyzed offline using ultrasound imaging software.

### Interventions

Both patient groups underwent treatment three times a week for six weeks. Exercise progression was determined by each tolerance and recovery rate.

**Fig 13:**
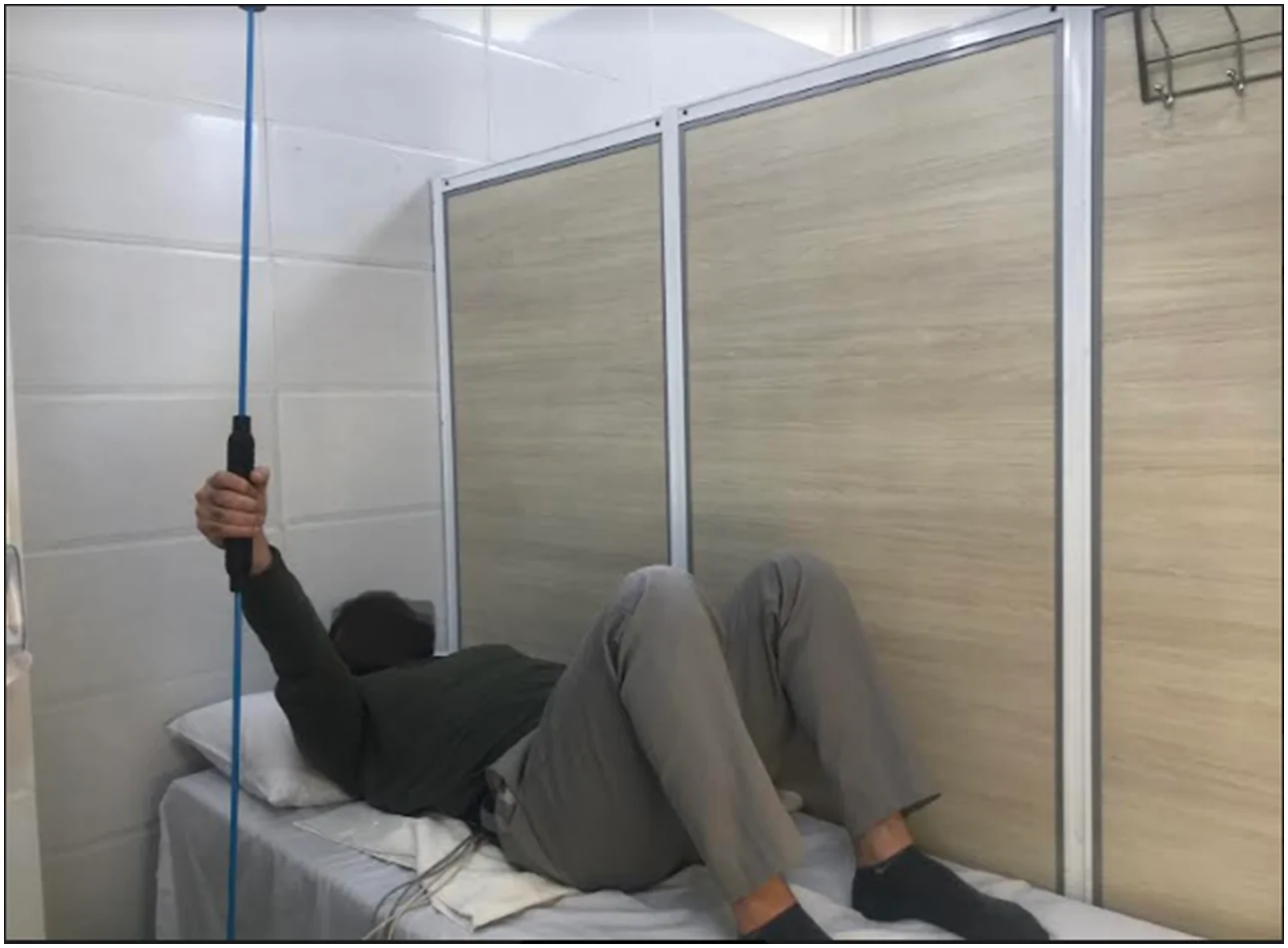
Stabilization exercises using a Flexi- Bar in the supine position. Created by the authors. Published under CC BY 4.0.

**Fig 14:**
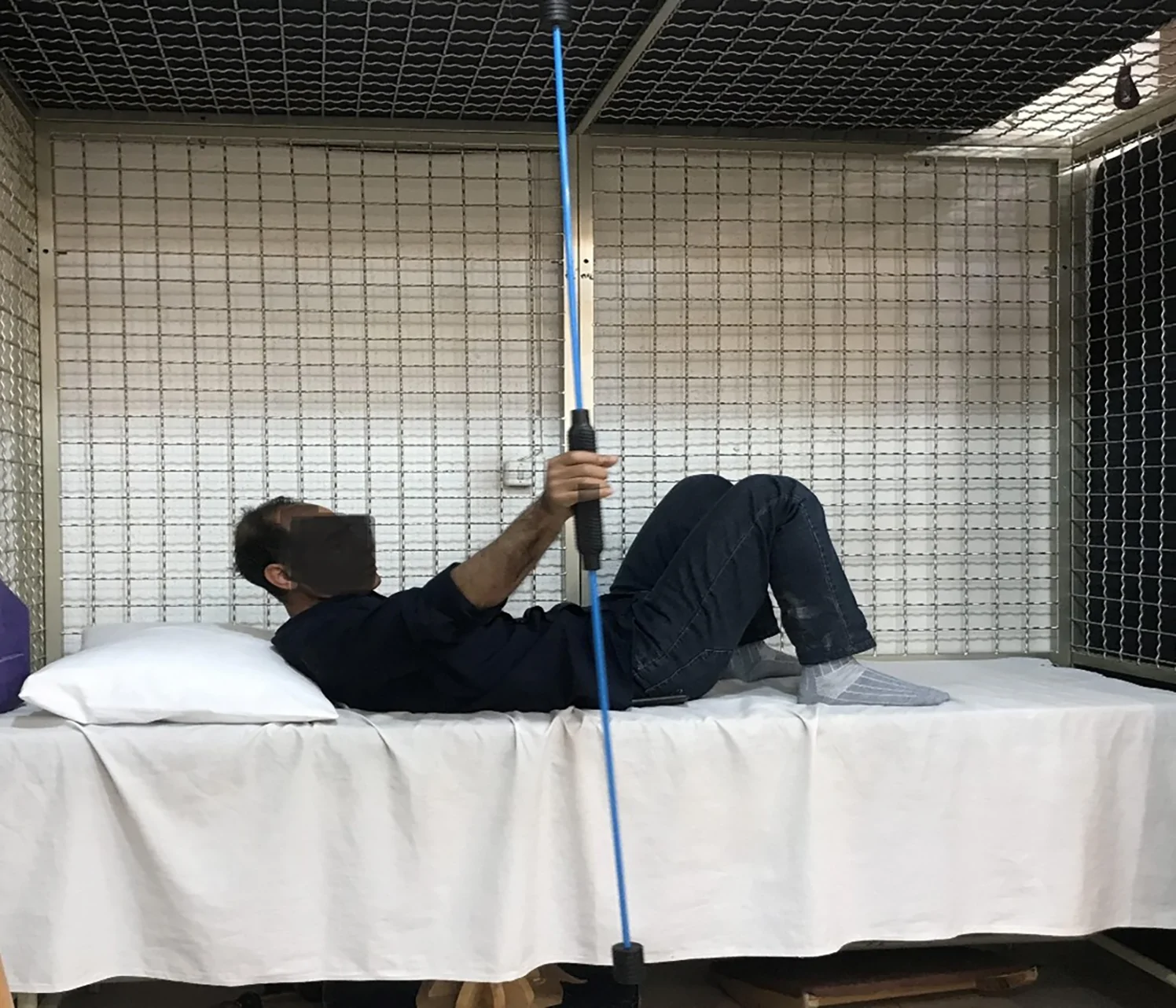
Stabilization exercise using a Flexi- Bar in the curl-up position. Created by the authors. Published under CC BY 4.0.

**Fig 15:**
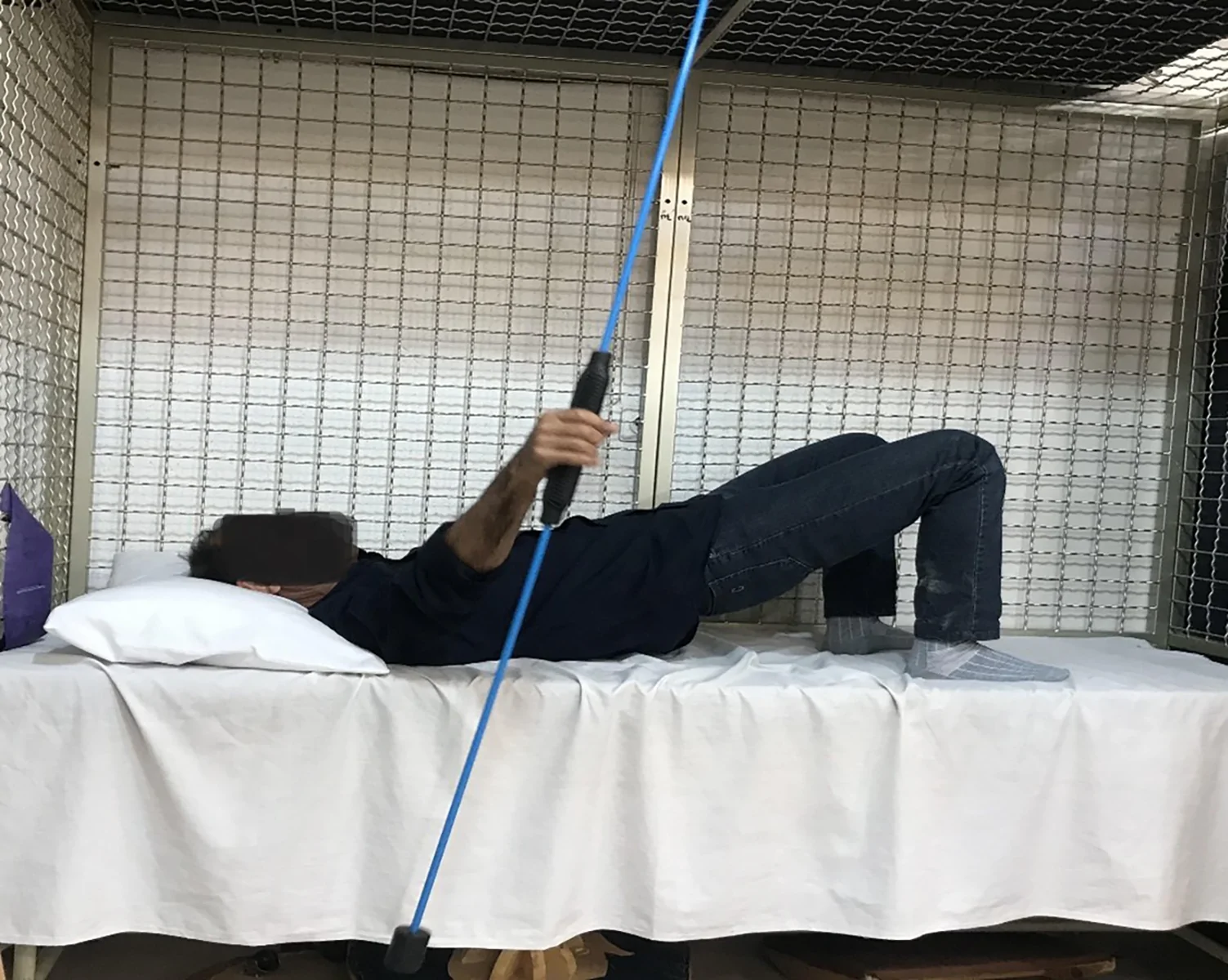
Stabilization exercise using a Flexi- Bar in the bridging position. Created by the authors. Published under CC BY 4.0.

**Fig 16:**
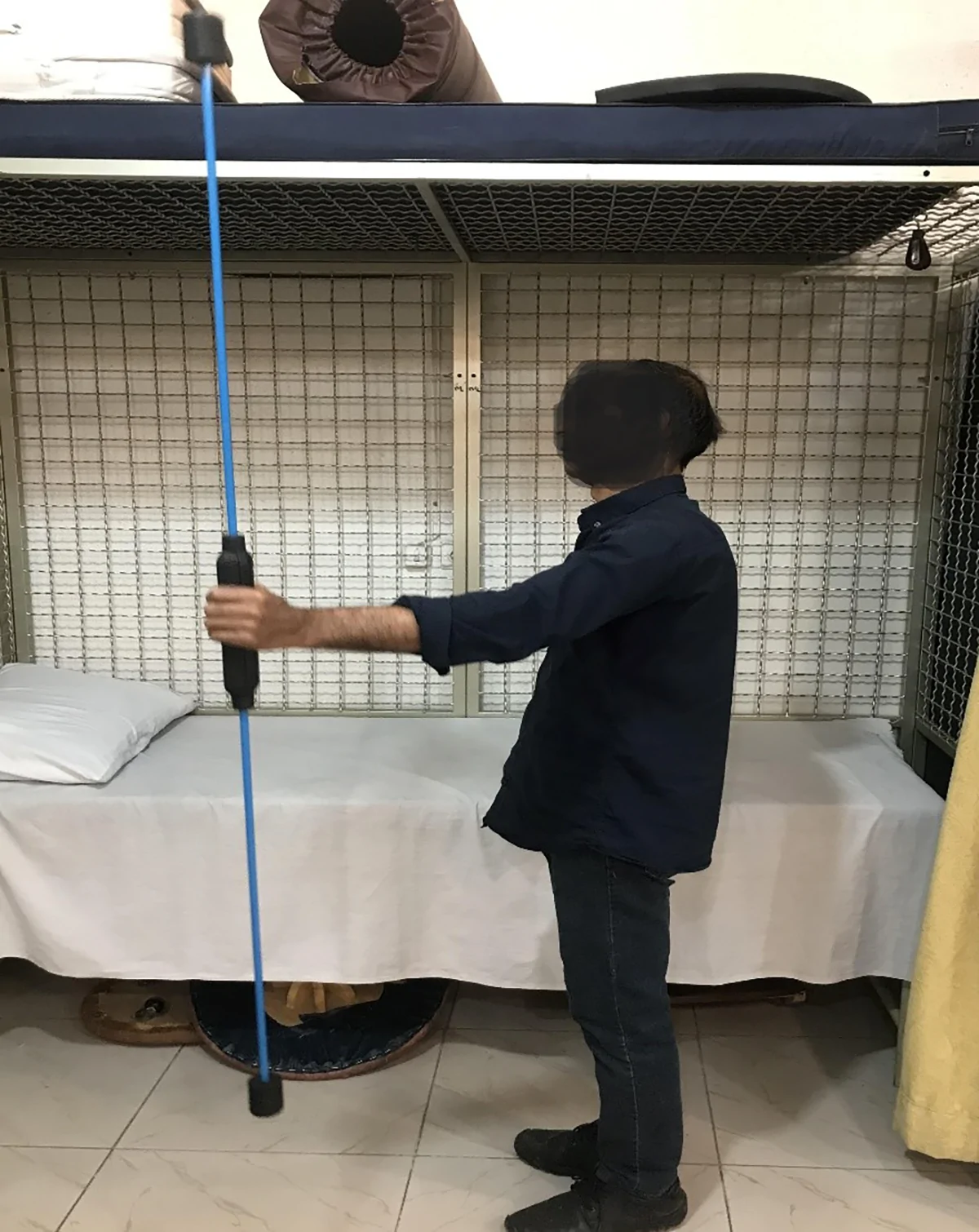
Stabilization exercise using a Flexi- Bar in the standing position. Created by the authors. Published under CC BY 4.0.

In both groups, each session began with 20 minutes of low-frequency Transcutaneous Electrical Nerve Stimulation (TENS) (frequency: 1–4 Hz, pulse duration: 100–400 µs, and amplitude adjusted to participant’s tolerance), followed by a 20-minute hot-pack application [47]. Before begining the exercises, participants were instructed in the ADIM, which involved drawing the navel inward and upward without pelvic tilt and maintaining the contraction for 10 seconds. Training intensity was progressively increased by adjusting oscillation frequency and amplitude across weeks 1–6. Stabilization exercises were performed exclusively with the Flexi-Bar (Flexi-bar Inc., Germany; length: 1530 mm, weight: 650 g). The device is pre-manufactured to generate standardized vibrations at a constant rate of 270 oscillations per minute (4.6 Hz) and was not custom-designed for this study. In the experimental group, after instruction in the proper use of the Flexi-Bar, participants performed stabilization exercises combined with PNE. Stabilization exercises included supine hook-lying, prone, curl-up, bridging, quadruped and standing positions, all incorporating the ADIM [48,49] (Fig 13–16). Each exercises consisted of three sets of ten repetitions, with each repetition involving 30 seconds of anterior–posterior oscillation performed with the dominant hand while maintaining 90 ° of shoulder flexion and full elbow extension throughout [49,50]. All participants completed these sessions three times a week for six consecutive weeks. Calibration of all Flexi-Bar devices was verified at baseline and remained unchanged throughout the trial. PNE was provided exclusively to the experimental group before each exercise session. Educational materials comprised a structured slide set with sixteen illustrations prepared by the research team specifically for this study. The content was adapted to a basic literacy level (approximately ninth-grade equivalent) to ensure clarity and engagement for all participants. In the control group, participants performed stabilization exercises using a Flexi- bar only.

Each session lasted 20–25 minutes, consistent with the protocol of Ismail Saracoglu et al. [20]**,** and was conducted once per week for six consecutive weeks**.** All sessions were delivered in person by a physiotherapist experienced in patient education. The presentation covered fundamental concepts of pain neurophysiology, including peripheral and central mechanisms of pain signaling, synaptic plasticity, and the distinction between nociception and pain perception. It further emphasized how cognitive and psychosocial factors could contribute to pain persistence. All sessions followed a standardized script to ensure treatment fidelity, and attendance was recorded for each participant. The same physiotherapist delivered all sessions to minimize variability.

To enhance participant understanding, simple metaphors and practical examples were used-for instance, comparing the nervous system to a home alarm system to illustrate over-sensitization). Visual diagrams accompanied each topic, and common everyday language was maintained throughout. No technical or medical jargon was included, allowing participants with diverse educational backgrounds to fully comprehend the information. This format ensured consistent delivery and minimized variability between sessions [20].

### Sample size calculation

A priori sample size estimation was performed using G Power* (version 3.1.10) based on the functional disability outcome measure reported by Bagheri et al. [51]. Assuming a type Ⅰ error rate (α) of 0.05, and a statistical power of 0.80. The estimation assumed two groups and three time points in a repeated‑measures design. According to these parameters, a minimum of 24 participants per group was required. Assuming a 10% attrition rate during follow-up, we recruited 26 participants per group to ensure adequate statistical power.

### Statistical analysis

All statistical analyses were performed using SPSS version 26 (IBM Corp., Armonk, NY, USA). All raw data are accessible in the Supporting Information file (S1 File). The Kolmogorov–Smirnov test examined the normal distribution of all variables. Repeated‑measures analysis of variance (ANOVA) was conducted to test the main effects of Time, Group, and their interaction (Time × Group) across three assessment points (baseline, immediately post‑intervention, and one‑month follow‑up). For repeated-measures ANOVA, the assumption of sphericity was examined using Mauchly’s test. When sphericity was violated, Greenhouse–Geisser corrections were applied, and corrected degrees of freedom are reported. When omnibus ANOVA tests showed significant effects, post‑hoc pairwise comparisons were conducted with Bonferroni adjustment to control for multiple testing. For within‑group contrasts across the three time points, the adjusted significance threshold was set at α_adj = 0.05/ 3 ≈ 0.017. Thus, comparisons between baseline vs. mid, baseline vs. post, and mid vs. post were Bonferroni-corrected. Between‑group p‑values presented in Tables 2 and 3 reflect the unadjusted omnibus ANOVA results, whereas time‑related post‑hoc comparisons are presented with Bonferroni‑adjusted p‑values. Effect sizes were interpreted as small (≤ 0.06), medium (0.06–0.14), or large (≥ 0.14) according to partial η² values. Statistical significance was set at p < 0.05 for all analyses.

## Results

Seventy individuals were initially screened for eligibility based on the predefined inclusion criteria. The trial report complies with the CONSORT 2010 statement (Supporting Information, S2 File). eighteen participants were excluded for not meeting the criteria, leaving fifty-two individuals with chronic non-specific low back pain included in the study (Fig 1). The fifty-two participants were randomly assigned to either the experimental group or the control group. After six weeks of treatment, four participants from both groups withdrew owing to non-compliance with the treatment protocol. Consequently, 48 participants completed the study and were included in the final analysis. Table 1 presents the baseline demographic and clinical characteristics of the patients. The results indicated no significant between-groups differences in any baseline variable (p > 0.05). For clinical endpoints, the repeated-measures ANOVA model first evaluated the overall main effects of Time, Group, and their interaction across the baseline–follow-up period. For ultrasound endpoints, which were assessed only at baseline and immediately post-intervention, the same model was used to identify significant main effects and interactions over this two-time-point period. Summaries of these omnibus results appear in Tables 2 and 3, followed by Bonferroni-adjusted comparisons for specific time points within each group.

**Table 1:** Baseline demographic and clinical characteristics of participants in the experimental (PNE + Flexi-Bar) and control (Flexi-Bar only) groups. Created by the authors. Published under CC BY 4.0.

Variables	Exp (n = 24) (mean ± SD)	Ctrl (n = 24) (mean ± SD)	P value
**Age**	**41.04 ± 7.42**	**41.33 ± 7.62**	**0.89**
**Sex(female/male)**	**(18/6)**	**(17/7)**	**1.00**
**Weight(kg)**	**70.92 ± 11.35**	**69.46 ± 13.25**	**0.68**
**Height(m)**	**1.66 ± 0.08**	**1.65 ± 0.09**	**0.54**
**BMI**	**25.49 ± 3.17**	**25.35 ± 2.99**	**0.87**

Significant at P ≤ 0.05.

P values obtained from independent t-test.

PNE: Pain Neuroscience Education, BMI: Body Mass Index, Exp: Experimental group, Ctrl: Control group.

**Table 2:** Results of repeated-measures ANOVA for clinical outcomes (RMQ, VAS, TSK, PCS, FABQ) showing main effects of Time, Group, and Time × Group. Created by the authors. Published under CC BY 4.0.

Outcome	Group	Baseline (Mean ±SD) [95% CI]	Post (Mean ±SD) [95% CI]	Follow-up (Mean ±SD) [95% CI]	Time effect (F, p, η² [S/M/L])	Group effect (F, p, η²)	Interaction (F, p, η²)
RMQ	Exp (n = 24)	14.2 ± 0.6 [11.2,13.8]	8.1 ± 0.5 [7.4,9.1]	7.4 ± 0.4 [6.2,8.4]	F = 78.37, p < 0.001, 0.63 [L]	F = 5.17, p = 0.02, 0.10 [M]	F = 10.11, p < 0.001, 0.18 [L]
	Ctrl (n = 24)	14.5 ± 0.6 [11.2,13.9]	9.5 ± 0.5 [9.6,12.3]	9.0 ± 0.4 [8.4,11.3]			
Pain	Exp (n = 24)	6.8 ± 0.4 [5.8,6.6]	3.9 ± 0.3 [3.2,4.2]	3.5 ± 0.3 [2.8,3.6]	F = 65.29, p < 0.001, 0.59 [L]	F = 6.12, p = 0.017, 0.12 [M]	F = 9.84, p = 0.003, 0.18 [L]
	Ctrl (n = 24)	6.9 ± 0.4 [5.8,6.6]	4.8 ± 0.3 [4.9,5.8]	4.6 ± 0.3 [3.7,4.4]			
PCS	Exp (n = 24)	32.5 ± 1.8 [29.0,36.6]	24.9 ± 1.6 [22.1,26.6]	24.1 ± 1.4 [19.9,24.4]	F = 50.71, p < 0.001, 0.52 [L]	F = 4.21, p = 0.045, 0.09 [S]	F = 8.17, p = 0.006, 0.15 [M]
	Ctrl (n = 24)	33.2 ± 1.7 [28.1,37.2]	26.5 ± 1.5 [23.0,29.1]	26.0 ± 1.3 [23.3,30.2]			
TSK	Exp (n = 24)	40.7 ± 1.5 [42.7,50.0]	33.1 ± 1.4 [30.2,36.5]	32.7 ± 1.2 [29.3,34.8]	F = 59.48, p < 0.001, 0.56 [L]	F = 5.88, p = 0.02, 0.11 [M]	F = 7.12, p = 0.01, 0.13 [M]
	Ctrl (n = 24)	41.1 ± 1.4 [42.3,49.0]	34.9 ± 1.3 [35.9,41.5]	34.2 ± 1.2 [35.6,40.9]			
FABQ	Exp (n = 24)	51.6 ± 2.1 [52.6,65.4]	39.0 ± 1.8 [33.4,43.1]	38.2 ± 1.7 [29.3,39.6]	F = 71.02, p < 0.001, 0.60 [L]	F = 4.55, p = 0.038, 0.09 [S]	F = 8.86, p = 0.005, 0.16 [M]
	Ctrl (n = 24)	52.1 ± 2.0 [50.7,64.8]	43.4 ± 1.8 [39.9,55.4]	42.9 ± 1.7 [38.5,51.3]			

Significant at P ≤ 0.05.

FABQ: Fear-Avoidance Beliefs Questionnaire, TSK: Tampa Scale for Kinesiophobia, PCS: Pain Catastrophizing Scale, RMQ: Roland-Morris Questionnaire; ctrl: control group, Exp: experimental group.

Values are derived from repeated-measures ANOVA with main effects of Time, Group, and Time × Group. Bonferroni correction was applied to within-group comparisons across the three time points (α_adjusted = 0.017). Unadjusted p-values for omnibus Group effects are displayed.

Partial η² interpreted as small (≤0.06), medium (0.06–0.14), large (≥0.14).

**Table 3:** Two-way mixed ANOVA results for abdominal muscle thickness (TA, IO, EO). Created by the authors. Published under CC BY 4.0.

Outcome	Effect	F)df(	F	P	Partial η²
EO_LT_SIT_CONT	Time	F (1, 46)	165.317	<0.001	0.782
	Group	F (1, 46)	9.002	0.004	0.164
	Time×Group	F (1, 46)	1.268	0.266	0.027
EO_LT_SIT_REST	Time	F (1, 46)	142.112	<0.001	0.755
	Group	F (1, 46)	2.386	0.129	0.049
	Time×Group	F (1, 46)	0.019	0.892	0.0
EO_LT_STAND_CONT	Time	F (1, 46)	162.69	<0.001	0.78
	Group	F (1, 46)	82.533	<0.001	0.642
	Time×Group	F (1, 46)	0.294	0.591	0.006
EO_LT_STAND_REST	Time	F (1, 46)	89.943	<0.001	0.662
	Group	F (1, 46)	10.403	0.002	0.184
	Time×Group	F (1, 46)	0.117	0.734	0.003
EO_LT_SUP_CONT	Time	F (1, 46)	413.935	<0.001	0.9
	Group	F (1, 46)	44.163	<0.001	0.49
	Time×Group	F (1, 46)	4.917	0.032	0.097
EO_LT_SUP_REST	Time	F (1, 46)	121.966	<0.001	0.726
	Group	F (1, 46)	18.544	<0.001	0.287
	Time×Group	F (1, 46)	0.144	0.706	0.003
EO_RT_SIT_CONT	Time	F (1, 46)	104.793	<0.001	0.695
	Group	F (1, 46)	1.993	0.165	0.042
	Time×Group	F (1, 46)	1.012	0.32	0.022
EO_RT_SIT_REST	Time	F (1, 46)	114.682	<0.001	0.714
	Group	F (1, 46)	22.079	<0.001	0.324
	Time×Group	F (1, 46)	0.661	0.42	0.014
EO_RT_STAND_CONT	Time	F (1, 46)	295.64	<0.001	0.865
	Group	F (1, 46)	9.177	0.004	0.166
	Time×Group	F (1, 46)	0.154	0.696	0.003
EO_RT_STAND_REST	Time	F (1, 46)	140.417	<0.001	0.753
	Group	F (1, 46)	34.21	<0.001	0.427
	Time×Group	F (1, 46)	0.336	0.565	0.007
EO_RT_SUP_CONT	Time	F (1, 46)	145.689	<0.001	0.76
	Group	F (1, 46)	0.031	0.86	0.001
	Time×Group	F (1, 46)	0.174	0.679	0.004
EO_RT_SUP_REST	Time	F (1, 46)	110.692	<0.001	0.706
	Group	F (1, 46)	0.491	0.487	0.011
	Time×Group	F (1, 46)	1.484	0.229	0.031
IO_LT_SIT_CONT	Time	F (1, 46)	112.357	<0.001	0.71
	Group	F (1, 46)	154.554	<0.001	0.771
	Time×Group	F (1, 46)	1.621	0.209	0.034
IO_LT_SIT_REST	Time	F (1, 46)	128.757	<0.001	0.737
	Group	F (1, 46)	252.294	<0.001	0.846
	Time×Group	F (1, 46)	0.146	0.704	0.003
IO_LT_STAND_CONT	Time	F (1, 46)	128.414	<0.001	0.736
	Group	F (1, 46)	103.519	<0.001	0.692
	Time×Group	F (1, 46)	1.878	0.177	0.039
IO_LT_STAND_REST	Time	F (1, 46)	128.101	<0.001	0.736
	Group	F (1, 46)	191.156	<0.001	0.806
	Time×Group	F (1, 46)	0.076	0.784	0.002
IO_LT_SUP_CONT	Time	F (1, 46)	49.191	<0.001	0.517
	Group	F (1, 46)	24.952	<0.001	0.352
	Time×Group	F (1, 46)	12.589	<0.001	0.215
IO_LT_SUP_REST	Time	F (1, 46)	187.383	<0.001	0.803
	Group	F (1, 46)	77.521	<0.001	0.628
	Time×Group	F (1, 46)	12.598	<0.001	0.215
IO_RT_SIT_CONT	Time	F (1, 46)	78.027	<0.001	0.629
	Group	F (1, 46)	44.357	<0.001	0.491
	Time×Group	F (1, 46)	0.098	0.756	0.002
IO_RT_SIT_REST	Time	F (1, 46)	273.025	<0.001	0.856
	Group	F (1, 46)	71.204	<0.001	0.608
	Time×Group	F (1, 46)	2.719	0.106	0.056
IO_RT_STAND_CONT	Time	F (1, 46)	109.644	<0.001	0.704
	Group	F (1, 46)	84.253	<0.001	0.647
	Time×Group	F (1, 46)	1.406	0.242	0.03
IO_RT_STAND_REST	Time	F (1, 46)	137.802	<0.001	0.75
	Group	F (1, 46)	14.421	<0.001	0.239
	Time×Group	F (1, 46)	4.47	0.04	0.089
IO_RT_SUP_CONT	Time	F (1, 46)	167.064	<0.001	0.784
	Group	F (1, 46)	27.637	<0.001	0.375
	Time×Group	F (1, 46)	2.531	0.118	0.052
IO_RT_SUP_REST	Time	F (1, 46)	160.364	<0.001	0.777
	Group	F (1, 46)	26.239	<0.001	0.363
	Time×Group	F (1, 46)	0.091	0.765	0.002
TA_LT_SIT_CONT	Time	F (1, 46)	64.752	<0.001	0.585
	Group	F (1, 46)	0.156	0.695	0.003
	Time×Group	F (1, 46)	1.785	0.188	0.037
TA_LT_SIT_REST	Time	F (1, 46)	55.682	<0.001	0.548
	Group	F (1, 46)	0.215	0.645	0.005
	Time×Group	F (1, 46)	3.087	0.086	0.063
TA_LT_STAND_CONT	Time	F (1, 46)	143.796	<0.001	0.758
	Group	F (1, 46)	0.381	0.54	0.008
	Time×Group	F (1, 46)	12.534	<0.001	0.214
TA_LT_STAND_REST	Time	F (1, 46)	130.018	<0.001	0.739
	Group	F (1, 46)	0.592	0.445	0.013
	Time×Group	F (1, 46)	11.942	0.001	0.206
TA_LT_SUP_CONT	Time	F (1, 46)	56.107	<0.001	0.549
	Group	F (1, 46)	0.653	0.423	0.014
	Time×Group	F (1, 46)	0.415	0.523	0.009
TA_LT_SUP_REST	Time	F (1, 46)	133.506	<0.001	0.744
	Group	F (1, 46)	0.485	0.49	0.01
	Time×Group	F (1, 46)	0.93	0.34	0.02
TA_RT_SIT_CONT	Time	F (1, 46)	83.815	<0.001	0.646
	Group	F (1, 46)	1.117	0.296	0.024
	Time×Group	F (1, 46)	3.393	0.072	0.069
TA_RT_SIT_REST	Time	F (1, 46)	82.458	<0.001	0.642
	Group	F (1, 46)	5.413	0.024	0.105
	Time×Group	F (1, 46)	8.951	0.004	0.163
TA_RT_STAND_CONT	Time	F (1, 46)	158.383	<0.001	0.775
	Group	F (1, 46)	25.874	<0.001	0.36
	Time×Group	F (1, 46)	11.014	0.002	0.193
TA_RT_STAND_REST	Time	F (1, 46)	156.045	<0.001	0.772
	Group	F (1, 46)	16.386	<0.001	0.263
	Time×Group	F (1, 46)	15.307	<0.001	0.25
TA_RT_SUP_CONT	Time	F (1, 46)	120.692	<0.001	0.724
	Group	F (1, 46)	6.919	0.012	0.131
	Time×Group	F (1, 46)	6.427	0.015	0.123
TA_RT_SUP_REST	Time	F (1, 46)	130.441	<0.001	0.739
	Group	F (1, 46)	0.571	0.454	0.012
	Time×Group	F (1, 46)	13.368	<0.001	0.225

TA: Transverse Abdominis, IO: Internal Oblique, EO: External Oblique, LT: left, RT: right, CONT: contract, SUP: supine, SIT: sitting,

All F and p-values are based on repeated-measures ANOVA. Bonferroni correction (α_adjusted = 0.017) was applied to pairwise comparisons of muscle geometry outcomes across time points. Raw p-values for Group effects are reported for clarity; values < 0.05 were considered significant after adjustment.

Partial η² interpreted as small (≤0.06), medium (0.06–0.14), large (≥0.14)

### Primary outcome

Detailed results of the repeated-measures ANOVA are presented in Table 2**.**

#### Disability.

**Fig 17:**
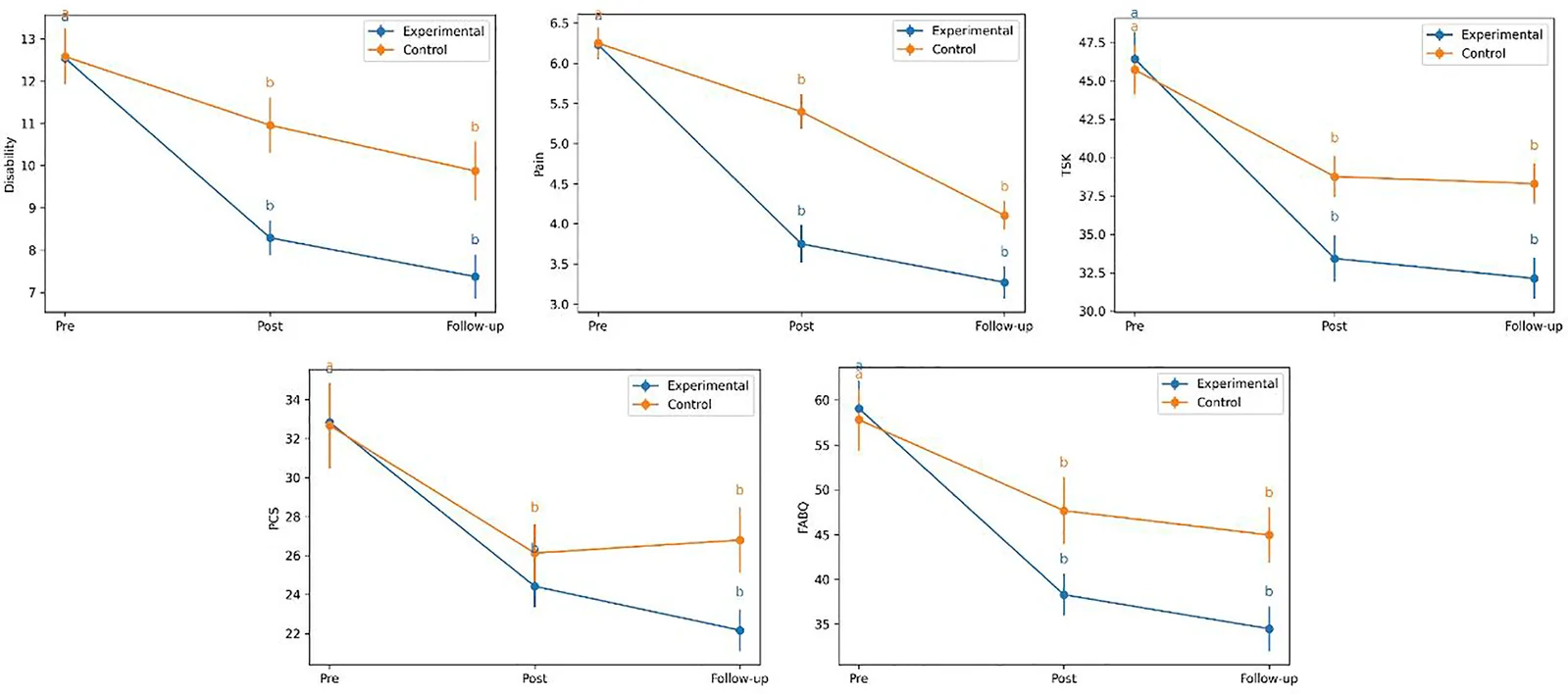
Profile plots of clinical outcomes across time points (Pre- intervention, Post- intervention, and 4-week follow-up) for the experimental and control groups: A) Functional disability (Roland-Morris Questionnaire, RMQ). **B)** Pain intensity (Visual Analogue Scale, VAS). **C)** Fear of movement (Tampa Scale for Kinesiophobia, TSK). **D)** Pain catastrophizing (Pain Catastrophizing Scale, PCS). **E)** Fear-avoidance beliefs (Fear-Avoidance Beliefs Questionnaire, FABQ). Different lowercase letters indicate significant differences between time points within each group based on Bonferroni-adjusted post-hoc comparisons. Time points sharing at least one letter are not significantly different.

A significant main effect of time was found for functional disability [(F (2, 92) = 78.371, p < 0.001 η² = 0.63)]. Between-group differences were also significant [(F (1, 46) = 5.167, p = 0.02, η² = 0.10)]. The time × group interaction was significant [(F (2, 92) = 10.105, p < 0.001, η² = 0.18)]. indicating a larger improvement in functional disability among participants in the experimental group. Bonferroni-adjusted post-hoc comparisons showed significant reductions in functional disability from baseline to follow up in both groups, as indicated by different letter annotation in Fig 17-A. No significant difference was observed between post-intervention and follow-up time points. Improvement was more pronounced in the experimental group (Table 2; Fig 17-A).

### Secondary outcomes

#### Pain.

A significant main effect of time was observed for pain intensity [(F (1.537, 70.690) = 158.471, p < 0.001, η² = 0.775]. The between-group difference was significant [(F (1, 46) = 12.970, p = 0.001, η² = 0.220)], with the experimental group showing greater reductions. The time × group interaction was also significant [(F (1.537, 70/690) = 15.576, p < 0.001, η² = 0.253)]. Effect sizes were large for both time and interaction.

Bonferroni-adjusted post-hoc comparisons showed significant decreases in pain intensity from baseline to post-intervention and follow-up in both groups, with greater reductions in the experimental group (Table 2; Fig 17-B).

### Fear of movement

A significant main effect of time was found for fear of movement [(F (2,92) = 221.303, p < 0.001, η² = 0.828)]. Between-group differences were non-significant [(F (1, 46) = 3.240, p = 0.07, η² = 0.066)]. The time × group interaction was significant [(F (2, 92) = 21.419, p < 0.001, η² = 0.318)]. indicating large reductions in fear of movement, particularly for the experimental group.

Bonferroni-adjusted post-hoc comparisons showed significant decreases in fear of movement from baseline to post-treatment and follow-up (p < 0.001) in both groups, with a sharper decline in the experimental group (Table 2; Fig 17-C).

#### Pain catastrophizing.

A significant main effect of time was observed for pain catastrophizing [(F (1.294, 59.510) = 77.869, p < 0.001, η² = 0.629)]. Between-group differences were not significant [(F (1, 46) = 0.942, p = 0.33, η² = 0.020)]. The time × group interaction was significant [(F (1.294, 59.510) = 5.449, p < 0.001, η² = 0.106)], suggesting large time effects and moderate interaction effects, indicative of greater improvement among participants receiving PNE.

Bonferroni-adjusted post-hoc comparisons revealed marked declines in pain catastrophizing from baseline to post-intervention and follow-up (p < 0.001) in both groups, with more pronounced improvement in the experimental group (Table 2; Fig 17-D).

#### Fear-avoidance beliefs.

A significant main effect of time was observed for fear-avoidance beliefs [(F (1,92) = 95.067, p < 0.001, η² = 0.508)]. Between-group differences were not significant [(F (1, 46) = 2.396/ p = 0.12, η² = 0.050)]. The time × group interaction was significant [(F (2, 92) = 9.980, p < 0.001, η² = 0.178)], reflecting large time and interaction effects.

Bonferroni-corrected post-hoc comparisons revealed a significant decline in FABQ scores from baseline to post-treatment and follow-up (p < 0.001), with larger reductions in the experimental group (Table 2; Fig 17-E).

### Ultrasound-based outcomes

Comprehensive mixed-design ANOVA analyses were conducted on muscle ultrasound measures (Time: pre-intervention vs. post-intervention × Group: experimental vs. control) for both the abdominal muscles and lumbar Multifidus.

All analyses revealed significant main effects of time for the TA, IO, EO, and lumbar Multifidus thicknesses, as well as for CSA changes (p < 0.05).

Detailed descriptive statistics, including means and standard deviations for ultrasound measurements, are provided in the Supplementary File (S1 Table).

Significant time × group interaction effects were observed across most conditions, indicating greater increases in muscle thickness and CSA in participants from the experimental group following the combined intervention. Post-hoc Bonferroni analyses demonstrated significant improvements from pre- to post-intervention in TA, IO, EO, and MF measurements, particularly within the experimental group across all three postural conditions (supine, sitting and standing).

Results are summarized in Table 3 (abdominal muscle outcomes) and Table 4 (multifidus outcomes). Descriptive mean ± SD values are presented in S1 Table.

**Table 4:** Two-way mixed ANOVA results for lumbar Multifidus thickness and CSA.

Outcome	Effect	F)df(	F	P	Partial η²
MF_AREA_LT_PRON_CONT	Time	F (1, 46)	290.815	<0.001	0.863
	Group	F (1, 46)	2.668	0.109	0.055
	Time×Group	F (1, 46)	12.369	<0.001	0.212
MF_AREA_LT_PRON_REST	Time	F (1, 46)	239.473	<0.001	0.839
	Group	F (1, 46)	2.303	0.136	0.048
	Time×Group	F (1, 46)	15.393	<0.001	0.251
MF_AREA_LT_SIT_CONT	Time	F (1, 46)	284.401	<0.001	0.861
	Group	F (1, 46)	6.25	0.016	0.12
	Time×Group	F (1, 46)	29.645	<0.001	0.392
MF_AREA_LT_SIT_REST	Time	F (1, 46)	182.859	<0.001	0.799
	Group	F (1, 46)	1.064	0.308	0.023
	Time×Group	F (1, 46)	8.41	0.006	0.155
MF_AREA_LT_STAND_CONT	Time	F (1, 46)	41.659	<0.001	0.475
	Group	F (1, 46)	131.132	<0.001	0.74
	Time×Group	F (1, 46)	18.182	<0.001	0.283
MF_AREA_LT_STAND_REST	Time	F (1, 46)	132.026	<0.001	0.742
	Group	F (1, 46)	218.523	<0.001	0.826
	Time×Group	F (1, 46)	22.964	<0.001	0.333
MF_AREA_RT_PRON_CONT	Time	F (1, 46)	274.805	<0.001	0.857
	Group	F (1, 46)	36.99	<0.001	0.446
	Time×Group	F (1, 46)	25.582	<0.001	0.357
MF_AREA_RT_PRON_REST	Time	F (1, 46)	200.977	<0.001	0.814
	Group	F (1, 46)	19.887	<0.001	0.302
	Time×Group	F (1, 46)	14.655	<0.001	0.242
MF_AREA_RT_SIT_CONT	Time	F (1, 46)	151.389	<0.001	0.767
	Group	F (1, 46)	10.437	0.002	0.185
	Time×Group	F (1, 46)	24.893	<0.001	0.351
MF_AREA_RT_SIT_REST	Time	F (1, 46)	122.524	<0.001	0.727
	Group	F (1, 46)	5.748	0.021	0.111
	Time×Group	F (1, 46)	17.498	<0.001	0.276
MF_AREA_RT_STAND_CONT	Time	F (1, 46)	237.111	<0.001	0.838
	Group	F (1, 46)	491.577	<0.001	0.914
	Time×Group	F (1, 46)	64.93	<0.001	0.585
MF_AREA_RT_STAND_REST	Time	F (1, 46)	229.588	<0.001	0.833
	Group	F (1, 46)	145.202	<0.001	0.759
	Time×Group	F (1, 46)	23.114	<0.001	0.334
MF_THICK_LT_PRON_CONT	Time	F (1, 46)	298.103	<0.001	0.866
	Group	F (1, 46)	38.733	<0.001	0.457
	Time×Group	F (1, 46)	130.828	<0.001	0.74
MF_THICK_LT_PRON_REST	Time	F (1, 46)	235.907	<0.001	0.837
	Group	F (1, 46)	112.986	<0.001	0.711
	Time×Group	F (1, 46)	74.824	<0.001	0.619
MF_THICK_LT_SIT_CONT	Time	F (1, 46)	218.853	<0.001	0.826
	Group	F (1, 46)	79.911	<0.001	0.635
	Time×Group	F (1, 46)	69.969	<0.001	0.603
MF_THICK_LT_SIT_REST	Time	F (1, 46)	117.095	<0.001	0.718
	Group	F (1, 46)	52.771	<0.001	0.534
	Time×Group	F (1, 46)	27.627	<0.001	0.375
MF_THICK_LT_STAND_CONT	Time	F (1, 46)	211.258	<0.001	0.821
	Group	F (1, 46)	32.301	<0.001	0.413
	Time×Group	F (1, 46)	78.518	<0.001	0.631
MF_THICK_LT_STAND_REST	Time	F (1, 46)	211.799	<0.001	0.822
	Group	F (1, 46)	50.174	<0.001	0.522
	Time×Group	F (1, 46)	80.796	<0.001	0.637
MF_THICK_RT_PRON_CONT	Time	F (1, 46)	194.698	<0.001	0.809
	Group	F (1, 46)	24.951	<0.001	0.352
	Time×Group	F (1, 46)	69.531	<0.001	0.602
MF_THICK_RT_PRON_REST	Time	F (1, 46)	110.586	<0.001	0.706
	Group	F (1, 46)	25.076	<0.001	0.353
	Time×Group	F (1, 46)	29.692	<0.001	0.392
MF_THICK_RT_SIT_CONT	Time	F (1, 46)	170.13	<0.001	0.787
	Group	F (1, 46)	73.29	<0.001	0.614
	Time×Group	F (1, 46)	32.693	<0.001	0.415
MF_THICK_RT_SIT_REST	Time	F (1, 46)	188.899	<0.001	0.804
	Group	F (1, 46)	56.894	<0.001	0.553
	Time×Group	F (1, 46)	33.321	<0.001	0.42
MF_THICK_RT_STAND_CONT	Time	F (1, 46)	208.253	<0.001	0.819
	Group	F (1, 46)	47.023	<0.001	0.505
	Time×Group	F (1, 46)	102.176	<0.001	0.69
MF_THICK_RT_STAND_REST	Time	F (1, 46)	118.098	<0.001	0.72
	Group	F (1, 46)	35.626	<0.001	0.436
	Time×Group	F (1, 46)	47.002	<0.001	0.505

Significant at P ≤ 0.05.

LT = left; RT = right, REST = resting state; CONT = contraction, SUP = supine; SIT = sitting; STAND = standing. Post-hoc pairwise comparisons were adjusted using Bonferroni correction Partial η² interpreted as small (≤0.06), medium (0.06–0.14), large (≥0.14).

No adverse events or exercise-related complications were observed or reported in either group throughout the study period.

## Discussion

The present study aimed to determine whether integrating PNE with Flexi-Bar–based stabilization training could produce superior clinical and morphological outcomes compared with stabilization exercises alone. These consistent patterns suggest that educational interventions may potentiate the effects of stabilization exercises by modulating maladaptive pain cognitions and promoting greater motor engagement. The results demonstrated significant improvements in functional disability, pain intensity, and fear-related beliefs across time in both groups, with larger changes observed in the integrated intervention arm [17,52,53]. In Kim et al. [17], disability did not improve significantly—likely attributable to the smaller sample size, all-male cohort, and older participants. Rabiei et al. [54] likewise reported consistent reductions in pain, functional disability, and fear-avoidance following motor-control exercise combined with PNE. In contrast, Kohns’s single-session PNE trial in chronic musculoskeletal pain patients found no immediate or follow-up changes in catastrophizing or fear of movement, underscoring the importance of intervention duration [55].

Although most outcomes reached statistical significance, the relatively small sample size limits the generalizability and precision of effect estimates. Consequently, interpretations should emphasize effect sizes and clinical relevance rather than isolated p-values.

At the four-week follow-up of the present trial, both groups maintained reductions in pain intensity compared to baseline, indicating sustained short-term benefits after the six-week program. However, only the PNE-integrated group preserved lower scores in pain catastrophizing, suggesting greater durability of the educational component. These findings align with previous work by Vibe et al*.* and Gallagher et al*.*, who reported similar maintenance of cognitive improvements following education-based interventions.[56,57]. Unlike Vibe et al.’s longer PNE protocol-which produced lasting decreases in fear-avoidance and disability- our six-week program did not yield enduring functional gains, suggesting that the dose of PNE may be critical for fully shifting avoidance behaviors. Previous research has shown that PNE alone can reduce maladaptive pain cognitions and improve movement behaviors in chronic low back pain [7]. However, the current study did not include a PNE-only group, limiting the ability to isolate the unique contribution of educational versus physical components. This limitation should be considered when interpreting the observed differences between-group.

Ultrasound assessment revealed significant increases in resting TA and MF thickness in the supine position for both groups, with more pronounced gains in the PNE group. These findings are consistent with Leonard et al., Akbari et al., and Kliziene et al [58–60]. In the sitting position, Finta et al. reported increases in resting TA and MF thickness but no changes during contraction [61], comparable to our resting-state findings but differing under contraction maneuvers. In standing, Ehsani *et al*. reported that stabilization exercises alone increased TA thickness [62]. No prior studies have examined MF geometry changes in standing after following combined PNE and exercise; nevertheless, given the thoracolumbar fascia’s role in force transmission, similar adaptations are plausible [58,60].

Our data indicated more pronounced increases in TA and multifidus thickness in the experimental group indicating that combining PNE with Flexi‑Bar stabilization may promote deep trunk muscle activation. The reduction in fear‑related beliefs likely helped participants engage these stabilizers more efficiently during oscillatory vibration exercises.

Consistent with our quantitative analysis, muscle-thickness changes were greatest in the standing position, which demands higher postural control compared to supine or sitting. This pattern is consistent with previous ultrasound studies reporting posture-dependent recruitment of trunk muscles [35].

Regarding oblique muscles in supine, both IO and EO showed significant thickening in both groups post-intervention. Between groups, significant changes favored PNE for left IO at rest and during contraction, and left EO during contraction. Unilateral Flexi-bar loading likely contributed to these side-specific adaptations [50]. Jae-Young’s six-week stabilization protocol increased resting TA, IO, and EO thickness [63], whereas Shamsi et al.’s shorter program and Vasseljen et al.’s older cohort showed no significant hypertrophy [21,64]. Bagheri et al. found that adding cognitive-behavioral therapy to stabilization exercises further enhanced TA thickness by reducing kinesiophobia and promoting deep-muscle activation [51].

In the sitting position, bilateral IO thickness increased significantly during contraction only in the PNE group. Although the ADIM primarily targets TA while down-regulating oblique activation [65], IO’s stabilizing role may explain its selective hypertrophy [66]. In standing, the PNE group exhibited increased left IO thickness during contraction; whereas the control group showed right-side EO thickening at rest, and the PNE group showed right EO changes during contraction. These findings align with Ehsani et al. [62]. Flexi-bar–induced rotational torque in predominantly right-handed individuals likely accentuated ipsilateral EO and contralateral IO recruitment [50], while standing’s narrower base of support imposes greater load demands on the EO [13].

Although strengthening is expected to increase muscle thickness at both rest and contraction, the present study primarily found greater changes in the contracted state. A critical consideration in interpreting our ultrasound findings is whether the observed increases in muscle thickness reflect true structural hypertrophy or enhanced neuromuscular activation. Given the relatively short 6‑week intervention period, true muscle hypertrophy—which typically requires 8–12 weeks of progressive resistance training—is unlikely to fully account for the observed changes. Instead, the more pronounced increases in muscle thickness during contraction compared with rest, particularly in the experimental group, suggest improved neuromuscular activation, motor unit recruitment, and volitional control of the deep stabilizing muscles. This interpretation is consistent with previous studies that similarly attributed short‑term ultrasound changes to enhanced activation rather than structural adaptations [51]. The reduction in fear‑related beliefs and kinesiophobia through PNE likely facilitated more efficient engagement of the transversus abdominis and multifidus during oscillatory vibration exercises, enabling participants to overcome protective inhibition and achieve better motor control. Furthermore, the posture‑dependent nature of our findings—with the greatest changes observed in standing, which demands higher postural control—supports a neuromuscular rather than purely structural interpretation [21].

Strengths of the present study include its randomized design, integrating of multimodal clinical and ultrasonographic outcomes, and comprehensive comparisons with previous literature. However, several limitations should be noted. The lack of a PNE-only group limits the ability to isolate the independent effects of PNE; however, the study aimed to assess the additional benefit of combining PNE with stabilization exercises. Moreover, the short four-week follow-up period and lack of ultrasound measurements at follow-up restricted the evaluation of sustained morphological adaptations.

Furthermore, while our sample size was determined a priori based on power calculations for the primary outcome, the relatively modest number of participants (n = 24 per group) may limit the precision of effect estimates and reduce generalizability to the broader chronic LBP population. Smaller samples are particularly susceptible to effect size overestimation and may not fully capture population heterogeneity in terms of age, symptom duration, pain severity, or treatment responsiveness. Accordingly, our interpretations emphasize effect sizes alongside statistical significance. Future large-scale, multi-center trials with more diverse samples are warranted to confirm and extend these preliminary findings.

## Conclusion

The combined intervention consisting of PNE and Flexi-Bar stabilization exercises resulted in significantly greater improvements in pain, functional disability, and thickness of the transversus abdominis and lumbar multifidus muscles in patients with chronic non-specific LBP compared with stabilization exercises alone. However, these findings are specific to the combined approach, and the absence of a PNE-only group precludes determination of whether the educational component independently contributes to these effects or whether the observed differences are influenced by non-specific factors such as increased therapeutic attention. Well‑designed three‑arm trials (PNE‑only, exercise‑only, and combined) with larger sample sizes and extended follow‑up are suggested to confirm these preliminary findings, disentangle the mechanisms underlying the observed improvements, and assess the durability of morphological and behavioral adaptations.

## Supporting information

S1 File Dataset. Raw dataset of the study, including demographic variables, clinical outcomes, and ultrasound measurements collected at baseline, post-intervention, and follow-up.
(CSV)

S2 File Checklist. Completed CONSORT 2010 checklist for reporting randomized clinical trials.
(DOCX)

S3 File Protocol (English). Final version of the study protocol in English, provided under the Creative Commons Attribution (CC BY 4.0) license.
(DOCX)

S4 File Protocol (Original). Original version of the study protocol in Persian, as approved by the Ethics Committee.
(DOCX)

S1 Table Main ultrasound muscle data presented as Mean ± SD, with Cohen’s *d* values and effect-size estimates for between-group comparisons. (DOCX)
