# Supplementary material for: The effect of integrating pain neuroscience education with stabilization exercises using a flexi-bar on functional disability, pain, and geometry of abdominal and multifidus muscles in patients with chronic non-specific low back pain
Source: PLoS One. 2026 Aug 27;21(8):e0355692. doi: 10.1371/journal.pone.0355692 (PMC13521579; doi:10.1371/journal.pone.0355692)
Supplement: Supplementary file 1 [file pone.0355692.s002.docx]

|  | Section/topic | No | CONSORT 2025 checklist item description | Reported on page no. |
| --- | --- | --- | --- | --- |
|  | **Title and abstract** | | |  |
|  | Title and structured abstract | 1a | Identification as a randomised trial | Identified as a randomized trial in the title |
|  |  | 1b | Structured summary of the trial design, methods, results, and conclusions | Structured abstract with trial design, methods, results, and conclusions (Abstract) page 2 line 30 |
|  | **Open science** | | |  |
|  | Trial registration | 2 | Name of trial registry, identifying number (with URL) and date of registration | Iranian Registry of Clinical Trials/ IRCT registration number: **IRCT20230505058095N1**  Registration date: **2023-06-02** |
|  | Protocol and statistical analysis plan | 3 | Where the trial protocol and statistical analysis plan can be accessed | The trial protocol and statistical analysis plan are included in the main manuscript file, which has been uploaded as part of the submission |
|  | Data sharing | 4 | Where and how the individual de-identified participant data (including data dictionary), statistical code and any other materials can be accessed | de-identified participant data and the statistical code used for analyses are available from the corresponding author upon reasonable request |
|  | Funding and conflicts of interest | 5a | Sources of funding and other support (eg, supply of drugs), and role of funders in the design, conduct, analysis and reporting of the trial | This study was financially supported by the Research Committee of Shiraz University of Medical Sciences. The funding body had no role in the design, conduct, analysis, or reporting of the trial. Page 8 line 130 |
|  |  | 5b | Financial and other conflicts of interest of the manuscript authors | None of the authors have any conflicts of interest to declare |
|  | **Introduction** | | |  |
|  | Background and rationale | 6 | Scientific background and rationale | On the pages 4-6 of the manuscript in the introduction section. |
|  | Objectives | 7 | Specific objectives related to benefits and harms | Specific objectives, including the hypotheses and expected benefits and harms are on the pages 4-6 Of the manuscript. |
|  | **Methods** | | |  |
|  | Patient and public involvement | 8 | Details of patient or public involvement in the design, conduct and reporting of the trial | No patient/public involvement |
|  | Trial design | 9 | Description of trial design including type of trial (eg, parallel group, crossover), allocation ratio, and framework (eg, superiority, equivalence, non-inferiority, exploratory) | This Study was a randomized, parallel-group clinical trial with an allocation ratio of 1:1 and a superiority framework. Further details are provided in the Method section page 7 |
|  | Changes to trial protocol | 10 | Important changes to the trial after it commenced including any outcomes or analyses that were not prespecified, with reason | No changes were made to the trial protocol after commencement of the study |
|  | Trial setting | 11 | Settings (eg, community, hospital) and locations (eg, countries, sites) where the trial was conducted | at the School of Rehabilitation Sciences, Shiraz University of Medical Sciences, Shiraz, Iran, page 7 |
|  | Eligibility criteria | 12a | Eligibility criteria for participants | Inclusion and exclusion criteria are described in the method section page 7 |
|  |  | 12b | If applicable, eligibility criteria for sites and for individuals delivering the interventions (eg, surgeons, physiotherapists) | Nothing |
|  | Intervention and comparator | 13 | Intervention and comparator with sufficient details to allow replication. If relevant, where additional materials describing the intervention and comparator (eg, intervention manual) can be accessed | Details of the interventions for each group, including type, duration, frequency, and provider characteristics, are described in the method section pages 14-15 |
|  | Outcomes | 14 | Prespecified primary and secondary outcomes, including the specific measurement variable (eg, systolic blood pressure), analysis metric (eg, change from baseline, final value, time to event), method of aggregation (eg, median, proportion), and time point for each outcome | Outcome measures included pain disability, TSK, PCS and FABQ assessed at baseline, immediately post-intervention, and at a 4-week follow-up. Additionally, geometry of abdominal and lumbar Multifidus muscles in supine, seated, and standing positions at baseline and immediately post-intervention in method section pages 9-13 |
|  | Harms | 15 | How harms were defined and assessed (eg, systematically, non-systematically) | Any adverse events were monitored and recorded throughout the study period. No adverse events were reported |
|  | Sample size | 16a | How sample size was determined, including all assumptions supporting the sample size calculation | Sample size determination considered a significance level of 0.05 and a power of 0.9 method section page7 |
|  |  | 16b | Explanation of any interim analyses and stopping guidelines | Nothing |
|  | Randomisation: |  |  |  |
|  | Sequence generation | 17a | Who generated the random allocation sequence and the method used | The allocation sequence was generated before the study using the “www.Randomization.com” website page 8 line 140 |
|  |  | 17b | Type of randomisation and details of any restriction (eg, stratification, blocking and block size) | A balanced block randomization method was used to randomly allocate participants into two groups. Method section page 8 |
|  |  |  |  | **Reported on page no.** |
|  | Allocation concealment mechanism | 18 | Mechanism used to implement the random allocation sequence (eg, central computer/telephone; sequentially numbered, opaque, sealed containers), describing any steps to conceal the sequence until interventions were assigned | Method section, Randomization page 8 |
|  | Implementation | 19 | Whether the personnel who enrolled and those who assigned participants to the interventions had access to the random allocation sequence | The random allocation sequence was generated through an online randomization system. According to this sequence, participant enrollment and group assignment were performed by the treating physiotherapist |
|  | Blinding | 20a | Who was blinded after assignment to interventions (eg, participants, care providers, outcome assessors, data analysts) | The assessor was blinded to group allocation during data collection page 8 line 150 |
|  |  | 20b | If blinded, how blinding was achieved and description of the similarity of interventions | The patients were requested not to disclose any information regarding group allocation to the assessor who was performing the assessments page 8 line 151-152 |
|  | Statistical methods | 21a | Statistical methods used to compare groups for primary and secondary outcomes, including harms | Statistical analyses were performed with SPSS v26. Normality was checked (Kolmogorov–Smirnov). Repeated-measures ANOVA with Bonferroni post-hoc was used to assess group × time effects. Independent t-tests compared muscle thickness/CSA between groups. Significance was set at p < 0.05. page 16 line 300 |
|  |  | 21b | Definition of who is included in each analysis (eg, all randomised participants), and in which group | Analyses were conducted on all participants who completed the intervention and follow-up assessments (n = 48; 24 per group). Participants were analyzed in the groups to which they were originally randomized (per-protocol analysis). |
|  |  | 21c | How missing data were handled in the analysis | Participants who withdrew (n = 4) were excluded from the final analysis. No imputation methods were used; only complete-case data were analyzed. |
|  |  | 21d | Methods for any additional analyses (eg, subgroup and sensitivity analyses), distinguishing prespecified from post hoc | No subgroup or adjusted analyses were performed.  All reported analyses (VAS, RMQ, TSK, PCS, FABQ, and ultrasound muscle measures) were prespecified. post hoc pairwise comparisons with bonferroni correction were conducted after significant group*time interactions. |
|  | **Results** | | |  |
|  | Participant flow, including flow diagram | 22a | For each group, the numbers of participants who were randomly assigned, received intended intervention, and were analysed for the primary outcome | 70 patients screened, 52 randomized (26 per group). |
|  |  | 22b | For each group, losses and exclusions after randomisation, together with reasons | Four participants in each group dropped out, leaving 48 (24 per group) for final analysis. Reasons: non-compliance, illness, travel, lack of interest. (See Figure 1 flow diagram). |
|  | Recruitment | 23a | Dates defining the periods of recruitment and follow-up for outcomes of benefits and harms | Recruitment: July 2023 – March 2024, Shiraz University of Medical Sciences. Follow-up duration: 4 weeks post-intervention |
|  |  | 23b | If relevant, why the trial ended or was stopped | - |
|  | Intervention and comparator delivery | 24a | Intervention and comparator as they were actually administered (eg, where appropriate, who delivered the intervention/comparator, how participants adhered, whether they were delivered as intended (fidelity)) | Intervention: The experimental group received PNE (six 30-min sessions) combined with Flexi-Bar stabilization exercises (18 sessions, three times per week).  Comparator: The control group received Flexi-Bar stabilization exercises only, with the same frequency and duration.  Both groups completed the sessions as planned, with no major deviations from the intervention protocol. |
|  |  | 24b | Concomitant care received during the trial for each group | Participants in both groups were instructed not to start new physiotherapy or pharmacological treatments during the trial.; reported use was minimal and balanced between groups. No additional concomitant interventions were provided. |
|  | Baseline data | 25 | A table showing baseline demographic and clinical characteristics for each group | Table 1 presents demographic and clinical characteristics at baseline; no significant between-group differences were observed. |
|  | Numbers analysed,  outcomes and estimation | 26 | For each primary and secondary outcome, by group:  ● the number of participants included in the analysis  ● the number of participants with available data at the outcome time point  ● result for each group, and the estimated effect size and its precision (such as 95% confidence interval)  ● for binary outcomes, presentation of both absolute and relative effect size | All 48 completers (24 per group) were analyzed; no imputation for missing data. Result section page 16 |
|  | Harms | 27 | All harms or unintended events in each group | No adverse events were reported. |
|  | Ancillary analyses | 28 | Any other analyses performed, including subgroup and sensitivity analyses, distinguishing pre-specified from post hoc | No subgroup or adjusted analyses were performed. |
|  | **Discussion** | | | **Page 30 line 513** |
|  | Interpretation | 29 | Interpretation consistent with results, balancing benefits and harms, and considering other relevant evidence | Adding PNE to stabilization exercises was superior to stabilization alone in reducing pain and disability and enhancing deep trunk muscle geometry. Effects are consistent with prior studies; sustained benefits may require longer PNE duration |
|  | Limitations | 30 | Trial limitations, addressing sources of potential bias, imprecision, generalisability, and, if relevant, multiplicity of analyses | Only patients with non-specific low back pain, young and middle-aged participants. Findings are applicable to young and middle-aged adults with chronic non-specific low back pain; caution in applying to older or more diverse populations. Page 31 line 558 |

Citation: Hopewell S, Chan AW, Collins GS, Hróbjartsson A, Moher D, Schulz KF, et al. CONSORT 2025 Statement: updated guideline for reporting randomised trials. BMJ. 2025; 388:e081123. <https://dx.doi.org/10.1136/bmj-2024-081123>
© 2025 Hopewell et al. This is an Open Access article distributed under the terms of the Creative Commons Attribution License (<https://creativecommons.org/licenses/by/4.0/>), which permits unrestricted use, distribution, and reproduction in any medium, provided the original work is properly cited.

*We strongly recommend reading this statement in conjunction with the CONSORT 2025 Explanation and Elaboration and/or the CONSORT 2025 Expanded Checklist for important clarifications on all the items. We also recommend reading relevant CONSORT extensions. See [www.consort-spirit.org](http://www.consort-spirit.org).
