# Supplementary material for: The effect of integrating pain neuroscience education with stabilization exercises using a flexi-bar on functional disability, pain, and geometry of abdominal and multifidus muscles in patients with chronic non-specific low back pain
Source: PLoS One. 2026 Aug 27;21(8):e0355692. doi: 10.1371/journal.pone.0355692 (PMC13521579; doi:10.1371/journal.pone.0355692)
Supplement: Supplementary file 2 [file pone.0355692.s003.docx]

**Type of Project:**

Fundamental  Applied  Fundamental–Applied

**10 – Introduction and Project Description:**

(*A: Introduction of the subject; B: Review of previous research; C: Necessity of carrying out the project and the applications of its results must be explained in detail in this section.*)

**Introduction**

Among musculoskeletal disorders, low back pain is the most common condition worldwide (1), defined as pain between the lower rib margins and the upper gluteal crease, with or without referral to the lower limbs (2). Despite pharmacological and surgical treatments, the prevalence of low back pain remains high (3), estimated at 60–80% among adults in the USA (4) and 25.2% annually among Iranians (5). Most adults (80–85%) experience low back pain at least once in their lifetime (6). Moreover, low back pain has been ranked as the fourth leading cause of disability globally (7). This condition is highly costly; in the United States, the annual healthcare costs related to low back pain are estimated at $100 billion (8).

Most episodes of low back pain are short-term and self-limiting. Nevertheless, more than 50% of all patients experience recurrence of symptoms within 22 months after receiving medical care (9), and between 4% and 25% progress to chronicity (10). Low back pain may be associated with heavy labor, obesity, smoking, and a sedentary lifestyle. In addition, psychosocial factors such as stress and anxiety play a major role in the development of low back pain (11).

Low back pain is mainly classified into three groups: mechanical, non-mechanical, and psychological. Most cases fall within the mechanical category, which is further divided into specific and non-specific low back pain. In the specific type, seen in 5% of the population, the cause and source of pain are identifiable, whereas in the non-specific type, accounting for 95% of cases, no clear cause or source for symptom onset is observed (12). In specific low back pain, causes may include disc herniation, spinal canal stenosis, lumbar spondylolisthesis, facet joint injury, malignancy, inflammatory diseases, and infection (13). Based on the duration of symptoms, patients are classified into acute (<4 weeks), subacute (4–12 weeks), and chronic (>12 weeks) groups (14).

**Disorders in Patients with Non-specific Low Back Pain**

Evidence shows that multiple disorders have been observed in patients with low back pain. These include reduced thickness and size of the transversus abdominis and multifidus muscles (15, 16), or increased rectus abdominis activity during sitting on an unstable surface compared to the transversus abdominis (18). Another disorder is uncontrolled movement in the lumbopelvic region (19). Patients with low back pain may have a greater range of motion in the lumbopelvic region compared to healthy individuals, which can damage the soft tissues. If this excessive movement repeatedly occurs in one direction, it may cause microtrauma and exacerbate low back pain (20).

Deficits in the range of motion of lower limb joints are also among lumbar region disorders (21). Studies have shown that in individuals with low back pain, the angle of internal rotation of the hip joint is less than in healthy individuals. Such hip movement restriction, in activities requiring full range in a specific trunk and hip direction, can worsen low back pain. Furthermore, patients with low back pain exhibit deep trunk muscle motor control impairment (22), balance control disorders such as increased postural sway (23), decreased postural alignment control due to proprioceptive processing disturbances (24), and asymmetrical weight-bearing between the lower limbs (25).

Studies have reported changes in deep abdominal muscles, especially the transversus abdominis, in patients with low back pain, including impaired anticipatory function (26), as well as functional changes (e.g., inability to contract) and morphological changes (e.g., altered size, asymmetry) in the multifidus muscle. Several studies have shown reduced automatic activation of the transversus abdominis during perturbation in patients with low back pain, potentially affecting predictive protective spinal control mechanisms (27).

**Disorders Related to the Size and Thickness of Abdominal Muscles and Multifidus in Patients with Chronic Non-specific Low Back Pain**

Abdominal muscles, including the transversus abdominis, internal oblique, and external oblique, contribute to spinal stability, alignment, and the balance between internal and external forces during functional activities (28). Deep muscles, such as the transversus abdominis and the posterior portion of the internal oblique, connect to the thoracolumbar fascia along with pelvic floor muscles and the diaphragm, enhancing spinal stability (29). Another important spinal muscle is the multifidus, which plays a major role in spinal stability, dynamic stability of the lumbopelvic region, and proprioception of the lumbar spine (30).

Ultrasound studies indicate that the thickness of the transversus abdominis, a spinal stabilizer, is reduced in patients with low back pain compared to healthy controls, with increased rectus abdominis activity observed in these patients (31). In a study examining symmetry of the lumbar multifidus cross-sectional area, patients with unilateral low back pain, compared to bilateral cases, showed greater thickness reduction on the more painful side at the L4 and L5 vertebral levels, suggesting that greater asymmetry may be associated with higher pain intensity (32).

Various methods, such as electromyography, MRI, and ultrasonography, are used to assess characteristics of abdominal and lumbar muscles and surrounding tissues, especially fat, in both healthy individuals and patients with low back pain. Among these, ultrasonography is suitable for observing contraction patterns of muscles because it permits visualization of deep contractile tissues, is less costly than MRI, entirely harmless, and non-invasive; evidence reports its reliability at ICC = 0.859–0.958 (33). Measurement of abdominal and multifidus muscle thickness in different positions—supine, sitting, and standing—has been investigated (34), with evidence showing highest reliability for the transversus abdominis in the supine position (ICC = 0.90). Acceptable reliability for the multifidus in supine and sitting positions has also been documented (34).

**Disorders Related to Changes in the Central Nervous System in Patients with Chronic Non-specific Low Back Pain**

Recent studies in patients with chronic low back pain have shown increased excitability of the central nervous system and brain tissue atrophy. Brain atrophy has been observed as reduced gray matter density in regions related to pain intensity and duration, including the prefrontal and somatosensory cortex, thalamus, and brainstem (35).

Additionally, in individuals with chronic non-specific low back pain, the pain experienced in response to stimulation of peripheral tissues away from the initial symptom site was markedly higher than stimulus-induced intensity. Although these individuals may exhibit changes in muscles, ligaments, and joints, the severity of these structural disorders shows weak correlation with clinical manifestations (36).

A mechanism explaining this phenomenon is central sensitization, seen in chronic low back pain patients, meaning amplification of neural signals within the central nervous system that increases pain sensitivity. One descending inhibitory mechanism regulating pain is conditioned pain modulation (CPM) (37). In animals, the activity of nociceptive dorsal horn neurons in response to unpleasant stimuli decreases. In humans, this condition is termed CPM, which increases pain thresholds outside the site of the unpleasant stimulus. CPM activity leads to reduced dorsal horn neural activity, decreased pain, and hypoalgesia.

A systematic review comparing CPM between individuals with chronic pain and healthy people concluded that CPM is impaired in chronic pain patients (38). CPM decreases with aging, and impairment is greater in women with chronic pain than in men (38). CPM is assessed using pain pressure threshold tests as a stimulus protocol. In chronic pain patients, inappropriate pain perception has been observed, linked to factors such as fear of movement and pain catastrophizing (39).

**Interventions Related to Disorders in Patients with Chronic Non-specific Low Back Pain**

Various non-pharmacological interventions, such as manual therapies (mobilization and manipulation), exercise therapy (stabilization, strength, and balance exercises), electrotherapy, massage, and cognitive-behavioral therapies, have been utilized for treating patients with low back pain. Regarding the treatment of chronic low back pain, exercise therapy can be effective in reducing pain, improving disability, enhancing back extension strength, mobility, and endurance (40). Forms of exercise therapy in these patients include aerobic exercises, strengthening, flexibility, stretching, stabilization exercises, motor control exercises, balance training, core exercises, and lumbar flexion exercises. Although previous studies have not definitively demonstrated the superiority of one type of exercise therapy over others, a systematic review observed that in patients with non-specific low back pain the combination of stabilization exercises with general exercises led to disability reduction over a shorter period compared to general exercises alone (41).

**Stabilization Exercises**

One form of exercise proposed for patients with chronic non-specific low back pain is stabilization training. Given that deep spinal muscle function, such as the transversus abdominis and multifidus, is impaired in these patients, such exercises, by influencing muscle movement control, can reduce pain and disability and improve function (42). The aim of trunk stabilization exercises is to enhance coordination between synergist and antagonist muscles and minimize unnecessary lumbopelvic movement to protect the trunk from microtrauma, pain, and degenerative changes (43). Previous studies have shown that stabilization exercises improve abdominal muscle thickness (e.g., transversus abdominis) (44). Stabilization training is widely based on using the abdominal drawing-in maneuver for contracting the transversus abdominis (45). This method employs isometric contractions to engage deep trunk muscles (46), minimizes global muscle contractions, and selectively contracts the transversus abdominis (47). Moreover, these exercises increase tension in the thoracolumbar fascia, thereby enhancing lumbopelvic stability (48).

Some recent studies on low back pain have incorporated vibration in patient exercises. Among the methods used to create vibration is the Flexi-Bar, a tool consisting of weights at each end of an elastic bar approximately 152 cm in length, which produces vibration at 5 Hz (49). Vibrations generated by the Flexi-Bar improve movement perception through proprioceptive stimulation (50). Exercises with the Flexi-Bar increase trunk muscle activity and the activity of abdominal and spinal muscles, hence recent studies using the Flexi-Bar have focused on trunk stabilizing muscles such as the transversus abdominis and multifidus (51). Furthermore, according to articles, such vibratory stimulation can, in addition to stimulating vibration receptors and reducing pain, activate muscle spindles, increase alpha motor neuron activity, and strengthen neuromuscular control in trunk muscles (52). This tool is used in various treatment centers in different positions to improve strength, coordination, and balance (51).

**Pain Neuroscience Education (PNE)**

Various strategies in treating patients with low back pain appear to have neglected aspects of pain perception, behavioral components, and pain physiology knowledge. Pain mechanisms are broadly categorized into three groups:

1. **Input mechanisms** including nociceptive pain and peripheral neuropathic pain.
2. **Processing mechanisms** including central pain, central sensitization, and cognitive-affective pain mechanisms.
3. **Output mechanisms** including autonomic, motor, neuro-hormonal, and immune systems (53).

Most recent interventions have focused on input and output mechanisms, with less attention to central mechanisms (54). One treatment approach recently applied to patients with chronic low back pain is PNE. This approach focuses on educating patients about mechanisms related to chronic pain, such as central pain or central sensitization and cognitive-affective pain mechanisms, and may facilitate the gradual return of patients to activities they fear performing (54).

PNE differs from traditional pain education in that its aim is to reduce nervous system sensitivity by focusing on delivering information about the neurophysiology, neurobiology, and meaning of pain, whereas traditional education emphasizes anatomical and biomedical models (55). Recent studies have shown that PNE can alter pain intensity, pain knowledge, disability, psychological functions, and pain-related behaviors in patients with musculoskeletal pain (56). However, it should be noted that PNE alone does not affect pain scores.

In PNE we encounter a **time-contingent** approach (performing a set number of repetitions of an exercise regardless of pain) versus a **symptom-contingent** approach (stopping exercise therapy upon onset of pain) (57). According to a case report study, cognitive-behavioral therapy in individuals with low back pain led to improvement in pain and disability, with changes observed in trunk muscle endurance, trunk, hip, and pelvic kinematics, and lumbar spine flexibility; it also altered movement strategies, improved load and pressure distribution, and reduced fear of applying load to the patient’s lumbar region (59).

**Review of Previous Research**

This section reviews studies conducted in three parts:

**A) Studies on the effect of stabilization exercises in patients with chronic non-specific low back pain**

- **Noor Mohammadpour et al. (2018)** conducted a study aimed at examining the effect of core stabilization exercises in treating nurses with chronic non-specific low back pain (60). Thirty-six female nurses with chronic low back pain were divided into intervention and control groups. Variables examined were pain, disability, quality of life, and thickness of abdominal muscles (transversus abdominis, internal oblique, external oblique). The intervention protocol consisted of 8 weeks of exercise therapy, with two ball-based and two floor-based exercises per week. Exercises included the ADIM maneuver in supine, quadruped, and sitting positions on the floor and Swiss ball. The control group received no intervention. Results showed significant improvement in pain, disability, quality of life, and abdominal muscle thickness in the intervention group compared to control.
- **Kwang-Jun Ko et al. (2018)** conducted a study to examine the effect of 12 weeks of lumbar stabilization exercises and sling exercises on lumbosacral angle, lumbar muscle strength, and pain in patients with chronic non-specific low back pain (61). Twenty-nine female patients with low back pain were divided into stabilization, sling, and control groups. Both therapy groups exercised for 60 minutes three times a week for 12 weeks. Variables examined were lumbosacral angle, lumbar muscle strength, and pain. Main exercises in both groups included:
  1. sit-up,
  2. superman exercise,
  3. quadruped arm and leg raise,
  4. squat,
  5. lower body fixation plank (surface or sling),
  6. upper body fixation plank (surface or sling),
  7. side plank (surface or sling),
  8. hip bridge (surface or sling).

Both groups performed exercises 1–4 on the floor. The stabilization group performed exercises 5–8 on the floor, while the sling group performed these with a sling. Results showed neither group affected lumbosacral angle, but flexor and extensor strength and pain severity improved significantly in both intervention groups compared to control.

- **Cho-Rong Bae et al. (2018)** compared sit-up exercises and core stabilization exercises in patients with chronic non-specific low back pain (62). Thirty-six patients were divided into two groups. Variables examined were pain, disability, abdominal muscle thickness using ultrasonography, and abdominal muscle activity using electromyography. In the stability group, participants performed ADIM, SLR in supine and prone positions, and opposite arm and leg raises in quadruped for 12 sessions. In the sit-up group, assisted sit-ups were done using HubEX-LEX (a sit-up machine). Results showed a significant difference pre- and post-treatment in the contraction/rest thickness ratio of rectus abdominis and external oblique in the sit-up group and transversus abdominis in the stability group. The ratio of internal oblique to transversus abdominis activity and pain/disability improved significantly in both groups.
- **Gheitasi et al. (2020)** investigated the effect of combining core stabilization exercises with sensorimotor training on pain, function, and fear of movement in athletes with chronic non-specific low back pain (63). Thirty male athletes with low back pain were divided into intervention and control groups. Pain, function, and fear of movement were assessed. The intervention group performed 8 weeks of stability training combined with sensorimotor exercises, including:
  1. abdominal drawing-in in prone position,
  2. quadruped with opposite arm and leg raise,
  3. modified side bridge for each side,
  4. supine on Swiss ball with static abdominal contraction,
  5. opposite arm and leg raise on Swiss ball,
  6. standing on one leg with knee flexed,
  7. standing on two legs in anatomical position with eyes closed,
  8. two-leg stance on balance board,
  9. standing on balance sandals with both legs,
  10. stepping on balance sandals,
  11. standing with knee flexed on balance sandals with both legs.

The control group received no therapeutic intervention. Results indicated significant improvement in pain, function, and fear of movement in the intervention group compared to control.

- **Sin Ho Chung et al. (2018)** examined the effect of lumbar stabilization exercises using a Flexi-Bar on pain, disability, activity capacity, and transversus abdominis thickness in patients with chronic non-specific low back pain (64). Twenty-seven patients were divided into experimental and control groups. Variables included pain (VAS), disability (ODI), transversus abdominis activity capacity (using pressure biofeedback units), and transversus abdominis thickness (ultrasonography). Both groups exercised for 30 minutes a day, three times a week, for six weeks. In the experimental group, ADIM was performed in standing, supine, prone, and quadruped positions for 10 seconds. In these positions, individuals held the Flexi-Bar with both hands (alternating hands in quadruped). Each movement was repeated 10 times in sets of 3 repetitions. The Flexi-Bar produced vibration at 4.6 Hz. The control group performed the same exercises without the Flexi-Bar. Results showed all variables improved in both groups after six weeks, with the experimental group showing greater improvement in all variables except transversus abdominis thickness.
- **Yue-mei Sun et al. (2021)** conducted a study aimed at investigating the effect of Flexi-Bar in patients with chronic non-specific low back pain (65). Thirty patients with low back pain participated in the study. Variables examined included pain (VAS), disability (ODI), transversus abdominis muscle thickness, multifidus cross‑sectional area, and differences in RMS values of electromyography of the multifidus and transversus abdominis muscles. In the experimental group, patients performed core stabilization exercises (supine bridge, curl‑up, and four‑point support) together with Flexi-Bar, while the control group performed the exercises without Flexi-Bar. Both groups exercised three times per week for six weeks. Results showed a significant difference in RMS values of multifidus and transversus abdominis muscles between the two groups. After exercise therapy, pain and disability values significantly decreased in both groups, and thickness and cross‑sectional area of the multifidus significantly increased; the increase in all variables except transversus abdominis thickness was greater in the experimental group compared to control.
- **General Conclusion:** Stabilization exercises in patients with non‑specific low back pain reduce pain, disability, improve abdominal muscle thickness, and reduce fear of movement. Moreover, Flexi-Bar is beneficial for improving pain and disability when combined with stabilization exercises in patients with non‑specific low back pain. However, its effect on trunk muscle activity and thickness requires further investigation.
- **B) Studies examining the effect of PNE in patients with chronic non‑specific low back pain**
- **Adriaan Louw et al. (2016)** conducted a systematic review to examine the effect of PNE in patients with chronic musculoskeletal pain (66). A systematic search was performed in 11 databases. All RCTs evaluating PNE in chronic musculoskeletal pain (low back pain, neck pain, and fibromyalgia) from 2002 to 2016 were included. Participants in included studies were aged 18 years and older, and variables assessed were pain, function, psychosocial factors, movement, and healthcare utilization. PNE topics covered in the studies included: pain neurophysiology, nociception and nociceptive pathways, synapses, action potentials, spinal facilitation and inhibition, peripheral and central sensitization, neural plasticity, physiological factors, and pain‑related beliefs. The longest education session lasted 4 hours, and the shortest was 30 minutes. Interventions provided alongside education included mobilization and manipulation, soft tissue massage, neural and muscle mobilization, trunk stabilization exercises, circuit aerobic training, dry needling, neck stabilization exercises, and hydrotherapy—or in some cases, neuroscience education alone. The review concluded that PNE improved pain, disability, fear of movement, trunk flexion, straight leg raise, and physical function in patients with low back pain.
- **Anneleen Malfliet et al. (2018)** conducted a study comparing the effect of PNE combined with cognitively targeted motor control training versus traditional pain education combined with general exercises in reducing pain, improving function, methodological characteristics of brain gray matter, and pain cognition in patients with chronic spinal pain (67). A total of 120 patients aged 18–65 years with chronic non‑specific spinal pain participated and were assigned to two groups: an experimental group (PNE plus cognitively targeted motor control training) and a control group (traditional low back and neck pain education plus general exercises). Variables evaluated included pressure pain threshold, numerical pain scale, central sensitization, gray matter methodological characteristics (using MRI), pain cognition (pain catastrophizing, fear of movement), disability, mental and physical health. Both intervention programs included three education sessions (group education, online home education, and individual education) and 15 one‑on‑one exercise sessions. In the experimental group, education addressed pain‑related beliefs, increased pain cognition, and reduced perceived threat. Content included neurons, synapses, nociceptive pathways, descending facilitation and inhibition, peripheral and central sensitization. Exercise programs focused on altering maladaptive beliefs and perceptions toward corrected understanding, introducing feared and avoided movements with gradually increasing complexity. The control group received traditional education on mechanical causes of neck and back pain, spinal anatomy, physiology and biomechanics, self‑care and ergonomics, joint forces and intervertebral disc pressure, lifting techniques, and endurance, stretching, and strengthening durations. Their exercise program targeted biomedical spinal dysfunctions (mobility, muscle strength and endurance, general fitness) progressing toward functional activities and physical tasks with the spine in a neutral position. Results showed the experimental group had greater improvement than control in pain pressure threshold, central sensitization symptoms, mental and physical functioning, fear of movement, and reduced disability.
- **Ismail Saracoglu et al. (2020)** investigated the effect of PNE combined with manual therapy and home exercises on pain, back performance, disability, and fear of movement in patients with chronic low back pain (68). Pain was assessed using NRS, back performance using Back Performance Scale, disability using Oswestry Disability Index, and fear of movement using Tampa Scale. Sixty‑nine patients aged 18–65 years were divided into three groups: (1) PNE plus manual therapy and home exercise; (2) manual therapy plus home exercise; (3) home exercise only (control group). All interventions lasted four weeks. In group 1, patients received posterior–anterior mobilization in prone position, mobilization with movement for lower lumbar vertebrae, and rotational forces in right and left side‑lying positions. They also received one PNE session weekly for four weeks (40–45 minutes each). The home exercise program for all groups included strengthening and stretching abdominal muscles, erector spinae, gluteals, quadriceps, and hamstrings. Results indicated that the PNE plus manual therapy group had greater improvement in pain and fear of movement compared to other groups. Disability improved significantly in groups 1 and 2 compared to control.
- **Ki‑Sang Kim et al. (2022)** examined the effect of PNE combined with lumbar stabilization exercises on strength and pain in patients with chronic non‑specific low back pain (69). Variables included pain, strength, flexibility, and activity disorder index. Thirty‑five women with chronic non‑specific low back pain were allocated to PNE plus stabilization exercise and stabilization exercise only groups. In the first group, participants received 10 minutes of PNE at the start of each session, followed by warm‑up and 20 minutes of stabilization exercises (hamstring, abdominal, and quadriceps stretches / curl‑up / dead bug / bridge / superman). The control group performed only 30 minutes of stabilization exercises. Results showed the PNE group had significantly greater improvement in pain, abdominal and back muscle strength, fear of movement, finger‑to‑floor test, and pain catastrophizing compared to the control group. However, no significant difference was observed between groups for disability or the modified Schober test.
- **General Conclusion:** Education related to pain physiology and neurophysiology in patients with chronic non‑specific low back pain, when combined with various physiotherapy interventions such as manual therapy and exercise therapy, has improved pain, disability, self‑efficacy, pain pressure threshold, central sensitization, pain cognition (pain catastrophizing, fear of movement), and mental and physical health. Most variables assessed in these studies were psychological, with less attention given to physical variables.
- **Necessity of the Study**
- Recent studies have shown changes in brain gray matter regions associated with pain duration and intensity in patients with chronic low back pain. Review of previous literature indicates that most common physiotherapy interventions have focused more on input and output mechanisms of pain and less on central mechanisms. Therefore, applying interventions that effectively target central mechanisms appears necessary. Considering this, the importance of addressing PNE becomes clear. PNE aims to provide patients with understandable explanations about mechanisms related to chronic pain, central and peripheral sensitization, and pain neurophysiology, contributing to reducing fear of movement, pain, and disability (66), enhancing self‑efficacy (69), increasing pain knowledge, reducing pain catastrophizing, and decreasing anxiety and depressive symptoms (66). However, PNE alone has limited impact on symptom improvement; evidence suggests combining it with other interventions such as manual therapy and various exercises has greater effect on improving symptoms in individuals with chronic pain. PNE also increases motivation to participate in exercise therapy and helps patients feel less disabled by returning to normal activities. By improving fear of movement and awareness that pain is not solely due to tissue damage but also results from altered central pain processing mechanisms, patients engage more actively in exercise programs (69).
- Furthermore, it has been observed that individuals with chronic low back pain tend to position their spine to experience minimal pain, leading to persistent maladaptive movement patterns. These patients suffer from kinesiophobia, and cognitive‑behavioral therapies can improve fear of movement. Studies have shown that changes in abdominal muscle thickness are explained 18% by pain variation and 82% by other factors such as psychological variables. Therefore, it is plausible that changes in fear‑avoidance beliefs can alter deep abdominal muscle activity in chronic non‑specific low back pain patients (70). Additionally, intramuscular fat changes in paraspinal muscles have been noted in low back pain patients. One possible mechanism for this is altered individual‑specific movement behaviors of the paraspinal muscles. Avoidance behaviors related to pain‑associated factors can contribute to muscular inhibition and changes in individual‑specific paraspinal muscle movement patterns, since individuals with negative pain perceptions avoid movements that provoke pain. Given that intramuscular fat is related to muscle cross‑sectional area in patients with chronic low back pain, interventions such as PNE that positively influence pain‑avoidance behaviors may have beneficial effects on reducing fat infiltration, improving intramuscular fat composition, and increasing muscle cross‑sectional area (71).

On the other hand, according to the results of recent studies, multiple musculoskeletal disorders have been observed in individuals with low back pain. Among these disorders are a reduction in the geometry of trunk muscles such as the transversus abdominis and multifidus muscles (18) and changes in the recruitment patterns of spinal stabilizing muscles, which lead to reduced stability of the spine (16). Evidence indicates that the effects of various exercises such as stability, motor control, and strengthening exercises in improving the thickness of abdominal and multifidus muscles have been established (72). Moreover, according to existing reports, stabilization exercises can reduce pain and disability and improve function in patients with low back pain (42). The aim of these exercises is to improve coordination of the muscles involved in spinal stability and to minimize unnecessary movements in the lumbopelvic region to protect the trunk against microtrauma, pain, and degenerative changes (73). Some recent studies on patients with low back pain have incorporated vibration into exercise programs and have shown that adding such vibrations to the therapeutic exercise protocol for patients with low back pain can result in greater improvement in pain and disability (74). The use of the Flexi-Bar is one method of producing vibration during exercises. Given that Flexi-Bar is a safe, low‑intensity stimulus that increases trunk muscle activity and can be used in various positions along with stabilization exercises (64), it appears to be a suitable tool for improving muscle activity.

Considering the above, since no study has yet examined the effect of stabilization exercises using Flexi-Bar combined with PNE on the geometry of abdominal and multifidus muscles in various positions in patients with non‑specific low back pain, in the present study, PNE education combined with stabilization exercises using Flexi-Bar will be applied in patients with low back pain to investigate whether adding PNE to stabilization exercises using Flexi-Bar can improve the variables under investigation, including pain, disability, and trunk muscle geometry.

**Objectives and Hypotheses**

**A) Objectives of the project:**

1. **General Goal:** To compare the effect of stabilization exercises using Flexi-Bar combined with PNE with stabilization exercises using Flexi-Bar alone on pain, disability, and trunk muscle geometry in patients with chronic non‑specific low back pain.
2. **Specific Objectives:**
   1. To determine and compare pain intensity before, immediately after, and one month after treatment using the VAS scale in the stabilization exercise with Flexi-Bar alone group and the stabilization exercise with Flexi-Bar + PNE group.
   2. To compare changes in pain intensity using the VAS scale immediately and one month after treatment between the stabilization exercise with Flexi-Bar + PNE group and the stabilization exercise with Flexi-Bar alone group.
   3. To determine and compare disability level before, immediately after, and one month after treatment using the Roland‑Morris Disability Questionnaire in the stabilization exercise with Flexi-Bar alone group and the stabilization exercise with Flexi-Bar + PNE group.
   4. To compare changes in disability using the Roland‑Morris questionnaire immediately and one month after treatment between the stabilization exercise with Flexi-Bar + PNE group and the stabilization exercise with Flexi-Bar alone group.
   5. To determine and compare the thickness of the transversus abdominis, internal oblique, external oblique, and multifidus muscles on both sides before and after treatment in the stabilization exercise with Flexi-Bar alone group and the stabilization exercise with Flexi-Bar + PNE group.
   6. To compare changes in the thickness of the transversus abdominis, internal oblique, external oblique, and multifidus muscles on both sides before and after treatment between the stabilization exercise with Flexi-Bar + PNE group and the stabilization exercise with Flexi-Bar alone group.
   7. To determine and compare the cross‑sectional area of the multifidus muscle on both sides before and after treatment in the stabilization exercise with Flexi-Bar alone group and the stabilization exercise with Flexi-Bar + PNE group.
   8. To compare changes in the cross‑sectional area of the multifidus muscle on both sides after treatment between the stabilization exercise with Flexi-Bar + PNE group and the stabilization exercise with Flexi-Bar alone group.
   9. To determine and compare fear of movement intensity before, immediately after, and one month after treatment using the Tampa Scale for Kinesiophobia in the stabilization exercise with Flexi-Bar alone group and the stabilization exercise with Flexi-Bar + PNE group.
   10. To compare changes in fear of movement intensity using the Tampa Scale for Kinesiophobia immediately and one month after treatment between the stabilization exercise with Flexi-Bar + PNE group and the stabilization exercise with Flexi-Bar alone group.
   11. To determine and compare pain catastrophizing intensity before, immediately after, and one month after treatment using the Pain Catastrophizing Scale in the stabilization exercise with Flexi-Bar alone group and the stabilization exercise with Flexi-Bar + PNE group.
   12. To compare changes in pain catastrophizing intensity using the Pain Catastrophizing Scale immediately and one month after treatment between the stabilization exercise with Flexi-Bar + PNE group and the stabilization exercise with Flexi-Bar alone group.
   13. To determine and compare fear‑avoidance belief intensity before, immediately after, and one month after treatment using the Fear‑Avoidance Beliefs Questionnaire in the stabilization exercise with Flexi-Bar alone group and the stabilization exercise with Flexi-Bar + PNE group.
   14. To compare changes in fear‑avoidance belief intensity using the Fear‑Avoidance Beliefs Questionnaire immediately and one month after treatment between the stabilization exercise with Flexi-Bar + PNE group and the stabilization exercise with Flexi-Bar alone group.
3. **Applied Purpose:**

Considering that PNE is a safe, simple, and low‑cost method, it can divert the patient’s attention away from focusing on damaged tissues, correct their mental beliefs, and create a new understanding of previous pain experiences. If combining this education with stabilization exercises results in improvement in pain, disability, and trunk muscle geometry, its application could significantly reduce treatment costs, minimize healthcare demands, enhance patient mobility, improve quality of life, and expedite their return to regular activities and participation in society.

**B) Research Hypotheses / Questions:**

1. Pain intensity measured by VAS before, immediately after, and one month after treatment differs between the stabilization exercise with Flexi-Bar alone group and the stabilization exercise with Flexi-Bar + PNE group.
2. Mean changes in pain intensity measured by VAS immediately and one month after treatment differ between the stabilization exercise with Flexi-Bar + PNE group and the stabilization exercise with Flexi-Bar alone group.
3. Disability level measured by the Roland‑Morris questionnaire before, immediately after, and one month after treatment differs between the stabilization exercise with Flexi-Bar alone group and the stabilization exercise with Flexi-Bar + PNE group.
4. Mean changes in disability measured by the Roland‑Morris questionnaire immediately and one month after treatment differ between the stabilization exercise with Flexi-Bar + PNE group and the stabilization exercise with Flexi-Bar alone group.
5. Changes in the thickness of the transversus abdominis, internal oblique, external oblique, and multifidus muscles on both sides before and after treatment differ between the stabilization exercise with Flexi-Bar alone group and the stabilization exercise with Flexi-Bar + PNE group.
6. Changes in the thickness of the transversus abdominis, internal oblique, external oblique, and multifidus muscles on both sides before and after treatment differ between the stabilization exercise with Flexi-Bar + PNE group and the stabilization exercise with Flexi-Bar alone group.
7. Changes in the cross‑sectional area of the multifidus muscle on both sides before and after treatment differ between the stabilization exercise with Flexi-Bar alone group and the stabilization exercise with Flexi-Bar + PNE group.
8. Changes in the cross‑sectional area of the multifidus muscle on both sides before and after treatment differ between the stabilization exercise with Flexi-Bar + PNE group and the stabilization exercise with Flexi-Bar alone group.
9. Fear of movement intensity measured by the Tampa Scale for Kinesiophobia before, immediately after, and one month after treatment differs between the stabilization exercise with Flexi-Bar alone group and the stabilization exercise with Flexi-Bar + PNE group.
10. Mean changes in fear of movement intensity measured by the Tampa Scale for Kinesiophobia immediately and one month after treatment differ between the stabilization exercise with Flexi-Bar + PNE group and the stabilization exercise with Flexi-Bar alone group.
11. Pain catastrophizing intensity measured by the Pain Catastrophizing Scale before, immediately after, and one month after treatment differs between the stabilization exercise with Flexi-Bar alone group and the stabilization exercise with Flexi-Bar + PNE group.
12. Mean changes in pain catastrophizing intensity measured by the Pain Catastrophizing Scale immediately and one month after treatment differ between the stabilization exercise with Flexi-Bar + PNE group and the stabilization exercise with Flexi-Bar alone group.
13. Fear‑avoidance beliefs intensity measured by the Fear‑Avoidance Beliefs Questionnaire before, immediately after, and one month after treatment differs between the stabilization exercise with Flexi-Bar alone group and the stabilization exercise with Flexi-Bar + PNE group.
14. Mean changes in fear‑avoidance beliefs intensity measured by the Fear‑Avoidance Beliefs Questionnaire immediately and one month after treatment differ between the stabilization exercise with Flexi-Bar + PNE group and the stabilization exercise with Flexi-Bar alone group.

**Study Implementation Method**

**A) Type of Study (please refer to the attached table):**

The design of this study is a randomized clinical trial that examines the effect of stabilization exercises using Flexi-Bar combined with PNE compared to stabilization exercises using Flexi-Bar alone in patients with chronic non‑specific low back pain.

**B) Sampling (sample size, method of selection, definition of sample with inclusion and exclusion criteria):**

Based on a similar study (75), with α = 0.05 (type I error) and 1−β = 0.99 (test power), the sample size was determined to be 21 individuals for each group. Considering a 10% dropout rate, the minimum number was adjusted to 23 participants for each group.

n=(Z1−α/2+Z1−β)2(σ12+σ22)(μ1−μ2)2 n = \frac{{(Z_{1-\alpha/2} + Z_{1-\beta})^2 (\sigma_1^2 + \sigma_2^2)}}{{(\mu_1 - \mu_2)^2}} n=(μ1−μ2)2(Z1−α/2+Z1−β)2(σ12+σ22)

μ1=26.5,σ12=16.07 \mu_1 = 26.5, \quad \sigma_1^2 = 16.07 μ1=26.5,σ12=16.07

μ2=46.7,σ22=13.4 \mu_2 = 46.7, \quad \sigma_2^2 = 13.4 μ2=46.7,σ22=13.4

The randomization method applied is double‑blind block randomization. One group is labeled with letter “A” and the other with letter “B,” and all possible arrangements of the two letters are written:

1. AB, 2) BA, 3) AA, 4) BB — numbered from 1 to 4. For each allocation, a random number between 1 and 4 is selected, and the chosen number determines the allocation pattern. For example, if number 2 is chosen, the first person will be assigned to group B and the next to group A.

Sampling will be conducted by posting advertisements in clinics and hospitals affiliated with Shiraz University of Medical Sciences, using a simple random method from among individuals with chronic non‑specific low back pain who attend these centers and physiotherapy clinics across Shiraz.

**Inclusion Criteria:**

- aged between 20 and 50 years
- reported LBP for at least three months
- scored 3-7 on the Visual Analogue Scale (VAS)
- held at least a high school diploma, and scored over 8 on the Roland-Morris Questionnaire (RMQ)(15)

**Exclusion Criteria:**

- history of lumbar spine malignancy or metastasis(16)
- acute infections(16)
- inflammatory or systemic diseases(17)
- structural spinal disorders(18)
- neurological conditions or brain injury(17)
- prior lower limb, abdominal or lumbar surgeries(19)
- pregnancy(20)
- recent physiotherapy or sports participation within the last three months(21)
- musculoskeletal or orthopedics disorders of the limbs(22)

**C) Instruments and Data Collection Process (a sample of the data collection form is attached):**

1. **VAS Scale**

**Pain:** Pain intensity was measured using a VAS from 0 (no pain) to 10 (highest pain level), a valid and reliable tool with an intra-class correlation coefficient (ICC) of 0.91 (23). The minimum clinically important change (MCID) for the VAS is 2 points (24)

2. **Roland‑Morris Questionnaire**

The RMQ is most sensitive for patients with mild to moderate disability due to low back pain, with a 24-item scale (0-24, where 0 = no disability and 24 = maximum disability). RMQ omitted describing the various levels of disability (for example, 40%–60% of disability is severe).The Persian RMQ version has an ICC of 0.83 (25). The MCID for the RMQ is 2.5 points (26).

3. **Tampa Scale of Kinesiophobia**

Tampa Scale of Kinesiophobia (TSK) is a self-reported questionnaire that quantifies fear of movement, or (re)injury, a 17-item tool that uses a 4-point Likert scale with Activity Avoidance and Somatic Focus subscales. Scores range from 17 (no fear) to 68 (highest fear). The Persian version of TSK demonstrated good reliability, with an ICC of 0.86 (27). Additionally, the MCID for this Questionnaire is 6 points (28).

4. **Pain Catastrophizing Scale**

The Pain Catastrophizing Scale (PCS) assessed participants' perceptions and feelings regarding pain (13 items, scored 0-4). A total score is yielded (ranging from 0-52), along with three subscale scores assessing rumination, magnification and helplessness. Higher score indicates higher level of catastrophizing. The Persian version has an ICC of 0.71 to 0.81 (29). Additionally, the MCID for this Questionnaire is 9.1 points (30).

5. **Fear‑Avoidance Beliefs Questionnaire**

Fear-avoidance beliefs were evaluated with the FABQ, which comprises 16 items across two domains (physical activity and work). Participants rate their level of agreement with each statement on a 7-point Likert scale (0= completely disagree to 6=completely agree), producing a total score from 0 to 96. A higher score indicates more strongly held fear avoidance beliefs. The Persian FABQ demonstrated good test-retest reliability (ICC=0.80) and the MCID for this questionnaire is 11 points (31, 32).

6. **Ultrasonography Device to Measure the Geometry of the Transversus Abdominis, Internal Oblique, External Oblique, and Lumbar Multifidus Muscles**

**Work Procedure:**

Volunteers meeting inclusion and exclusion criteria will enter the study after completing informed consent and being informed of the procedure. Demographic information such as age, sex, height, weight, BMI, medical history, date of referral, and medications will be recorded. After completing pain, disability, fear of movement, and pain catastrophizing questionnaires, muscle thickness measurements and multifidus cross‑sectional area will be obtained using an HS‑2600 ultrasound device (Hyundai, Japan).

Images will be captured with a 7.5‑MHz linear transducer using B‑mode ultrasound, with resolution and gain adjusted for clarity. For abdominal muscle thickness, participants will lie supine with knees bent, and the examiner will place the transducer transversely at the midpoint of the anterior axillary line between the ASIS and the last rib (28). Images will be taken at rest and during the abdominal drawing‑in maneuver at end‑expiration, in hook‑lying position, with two measurements on each side; each contraction held for 5–10 seconds (75).

For multifidus thickness and cross‑sectional area, the participant will lie prone, arms alongside the body, head positioned centrally, with a pillow under the abdomen to reduce lumbar lordosis (88). The probe center will first be placed longitudinally over the L4 spinous process, then moved 2 cm laterally and angled ~10° to visualize the L5–L4 zygapophyseal joint for a parasagittal image of the multifidus at rest (89). Thickness is measured from the muscle fascia beneath subcutaneous tissue to the echogenic lamine shadow. Cross‑sectional imaging is done transversely at L4 to capture both sides; if both cannot be captured simultaneously, each side is imaged separately. Muscle borders are defined using anatomical landmarks including the thoracolumbar fascia (superior), lamine shadow (deep), spinous process acoustic shadow (medial), and separating fascia from longissimus (lateral).

Area calculation uses two orthogonal lines: one along the maximum anterior–posterior depth, and another perpendicular at the maximum lateral width, giving the area (88). Thickness is measured in millimeters between inner fascia borders (79). Images are obtained at L4/L5 bilaterally in rest and contraction. For contraction, a weight corresponding to ~30% of maximal voluntary isometric contraction is held for 3 seconds at end‑expiration, repeated three times each side, with shoulder at 120° abduction and elbow at 90° flexion. Weight is chosen by body mass (<68.2 kg = 0.68 kg; 68.2–90.9 kg = 0.9 kg; >90.9 kg = 1.36 kg).

Subsequently, measurements are repeated in sitting (chair height 43 cm, neutral spine, arms crossed over opposite shoulders) and standing (neutral posture after walking in place). Contraction in sitting and standing uses ADIM for abdominal muscles and the weighted arm lift for multifidus. Standing multifidus contraction: weight held with shoulder at 90° flexion and elbow fully extended, held for 3 seconds before imaging (16). Thickness values for sitting and standing are normalized to resting supine thickness.

Participants will be randomly assigned into two groups: the experimental group (therapeutic exercise with stability training using flexibar combined with PNE) and the control group (therapeutic exercise with stability training using flexibar alone). The exercise program consists of three sessions per week for six weeks (64). In both groups, at the beginning of each treatment session, electrotherapy will be performed, including 20 minutes of low‑frequency TENS (91) (frequency 1–4 Hz, duration 100–400 microseconds, and amplitude tolerated by the patient), a hot pack for 20 minutes, and continuous ultrasound at a frequency of 1 MHz and intensity of 1.5 W/cm² for 5 minutes. At the start and end of each session, participants will perform warm‑up and cool‑down exercises for 5 minutes.

Before performing the exercises, both groups will be instructed to draw the abdomen upward and inward to create an abdominal concavity, then, while maintaining a neutral spinal position, perform the ADIM maneuver for 10 seconds. Participants in both groups will then carry out stabilization exercises using the flexibar. For this purpose, individuals, in standing, hook‑lying, quadruped, and prone positions, will perform the ADIM maneuver for 10 seconds, as well as exercises such as supine bridge, curl‑up, and four‑point support (65). To hold the flexibar, participants will use one hand, moving it vertically in an anterior–posterior direction while the shoulder is in 90° flexion (92). Each movement will be repeated 10 times in three sets, with a 3‑minute rest between sets (64).

After completing the stabilization exercises using the flexibar, the experimental group will receive PNE training once a week (68) for six weeks. These sessions will be conducted individually for 30–60 minutes (69) using prepared slides, and will generally cover topics such as the neurophysiology of pain, nociception and nociceptive pathways, synapses, action potentials, spinal facilitations and inhibitions, peripheral and central sensitization, neuroplasticity, physiological factors, and beliefs related to pain.

**Data Analysis:**

Descriptive statistics will include mean, standard deviation, frequency, and percentage.

Inferential statistics will include independent sample t‑test or Mann–Whitney test, paired sample t‑test or its non‑parametric equivalent the Wilcoxon test, repeated‑measures test or its non‑parametric equivalent the Friedman test.

SPSS software version 26 will be used for data analysis, with the significance level set at 0.05.
