# Supplementary material for: The effect of integrating pain neuroscience education with stabilization exercises using a flexi-bar on functional disability, pain, and geometry of abdominal and multifidus muscles in patients with chronic non-specific low back pain
Source: PLoS One. 2026 Aug 27;21(8):e0355692. doi: 10.1371/journal.pone.0355692 (PMC13521579; doi:10.1371/journal.pone.0355692)
Supplement: Supplementary file 3 [file pone.0355692.s004.docx]

**با نام خدا**

## **دانشگاه علوم پزشكي و خدمات بهداشتي درماني فارس**

### معاونت پژوهشي وفناوری

# پرسشنامه طرح پژوهشي

**نام و نام خانوادگي مجري اول طرح : دکتر سارا ابوالاحراری شیرازی**

**عنوان طرح : بررسی تاثیر افزودن PNE به تمرینات ثباتی با استفاده از فلکسی بار بر درد ، ناتوانی و ژئومتری عضلات تنه در بیماران مبتلا به کمر درد مزمن غیر اختصاصی**

**تاريخ ارائه طرح به معاونت پژوهشي :**

**نشاني :شيراز خيابان زند – ساختمان مركزي دانشگاه علوم پزشكي**

#### **صندوق پستي 1978-71345**

[**vcrdep@sums.ac.ir**](mailto:vcrdep@sums.ac.ir)

**پرسشنامه طرح تحقيقاتي**

قسمت اول - اطلاعات مربوط به مجري طرح و همكاران

1- **نام ونام خانوادگي مجري اول** : دکتر سارا ابوالاحراری شیرازی
2- **نشاني محل كار**: دانشکده ی علوم توانبخشی

**تلفن محل كار :** 00987132122600

تلفن همراه :00989173111044

**نشاني پست الكترونيك** : SAhrari@Sums.ac.ir

3- **شغل و سمت فعلي و مرتبه علمي مجري اول :** عضو هیئت علمی دانشکده ی توانبخشی-استادیار
4- **سازمان متبوع** : دانشگاه علوم پزشکی شیراز
5- **محل اجراي طرح** : دانشکده ی علوم توانبخشی
6- **درجات علمي و سوابق تحصيلي مجري اول ( به ترتيب از ليسانس به بالا ) :**

| **درجه تحصيلي** | **رشته تحصيلي و تخصصي** | **دانشگاه** | **كشور** | **سال دريافت** |
| --- | --- | --- | --- | --- |
| کارشناسی | فیزیوتراپی | علوم پزشکی شیراز | ایران | 1375 |
| کارشناسی ارشد | فیزیوتراپی | علوم پزشکی شیراز | ایران | 1384 |
| دکتری تخصصی | فیزیوتراپی | علوم پزشکی شیراز | ایران | 1396 |

7- **فهرست پژوهشهاي قبلي و مقالات خود را مرقوم فرماييد .**

**( در صورت طولاني بودن فهرست لطفاً فقط پژوهشها و مقالات مرتبط با طرح فعلي و يا 7 مورد پژوهشهای اخيررا درج نماييد . )**

| **ردیف** | **عنوان پژوهش های قبلی** | **محل چاپ نام نشريه ، شماره و تاریخ انتشار** | **چاپ نگردیده** | **درحال اجرا** |
| --- | --- | --- | --- | --- |
|  |  |  |  |  |

**8-مشخصات همكاران اصلي :**

| **نام ونام خانوادگي** | **آخرین مدرک تحصیلی** | **رشته تخصصي** | **درجه علمي** | **نوع همكاري** | **امضاي همكار** |
| --- | --- | --- | --- | --- | --- |
| **دکتر فرحناز امامی** | **دکتری تخصصی** | **فیزیوتراپی** | **استادیار** | **مشاوره ی تخصصی در اجرای طرح** |  |
| **زهرا دهقانی** | **کارشناسی** | **فیزیوتراپی** | **دانشجوی کارشناسی ارشد** | **جمع آوری داده ها و انجام تحقیق** |  |
| **غزل رشدی** | **کارشناسی ارشد** | **فیزیوتراپی** | **دانشجوی دکترا تخصصی** | **ارزیابی بیماران** |  |

**قسمت دوم-اطلاعات مربوط به طرح پژوهشي**

**9- عنوان طرح پژوهشي و نوع طرح**

**الف-عنوان فارسی**

**بررسی تاثیر افزودن PNE به تمرینات ثباتی با استفاده از فلکسی بار بر درد ، ناتوانی و ژئومتری عضلات تنه در بیماران مبتلا به کمر درد مزمن غیر اختصاصی**

**ب-عنوان انگلیسی**

The effect of integrating pain neuroscience education with stabilization exercises using a Flexi-Bar on pain, disability, and geometry of abdominal and Multifidus muscles in patients with chronic non-specific low back pain, Randomized clinical trial.

**ج- نوع طرح :**

بنیادی	کاربردی	بنیادی-کاربردی

**10-مقدمه و معرفي طرح :**

( الف :معرفي موضوع ، ب-مروری برپژوهشهاي قبلي ج- ضرورت انجام طرح و موارد كاربـــرد نتايج آن می بايست در اين قسمت به تفصیل شرح دهيد )‌

**مقدمه**

از میان اختلالات عضلانی- اسکلتی، کمردرد شایع ترین عارضه در سرتاسر جهان بوده (1) و بصورت درد بین حاشیه دنده های تحتانی و بالای چین گلوتئال با یا بدون ارجاع به اندامهای تحتانی تعریف می شود (2). علی رغم درمان های دارویی و جراحی شیوع کمر درد همچنان بالا است (3)به طوری که شیوع آن 60 تا 80 % در جمعیت بزرگسالان آمریکا (4) و 25.2% به صورت سالانه در جمعیت ایرانیان (5)تخمین زده شده است. اغلب جمعیت بزرگسالان (80 تا 85 %) در طول زندگی خود حداقل یک بار کمر درد را تجربه کرده اند (6). به علاوه کمر درد به عنوان چهارمین علت ایجاد ناتوانی در سطح جهانی رتبه بندی شده است(7). این عارضه بسیار هزینه بر می باشد به طوری که در جمعیت ایالت متحده ی آمریکا هزینه های درمانی مرتبط با کمر درد سالانه 100 بیلیون تخمین زده شده است(8) . بیشتر دوره های کمر درد کوتاه مدت و خود محدود شونده است ، با این وجود بیشتر از 50 درصد همه ی بیماران در 22ماه بعد از دریافت مراقبت های درمانی دچار بازگشت علائم کمر درد می شوند(9) و بین 4 تا25 % بیماران به سمت مزمن شدن بیماری می روند (10). کمر درد می تواند با کار سنگین ، چاقی ، سیگار و سبک زندگی بی تحرک ارتباط داشته باشد. به علاوه ، عوامل روانی- اجتماعی مانند استرس و اضطراب در ایجاد کمر درد نقش عمده ای ایفا می کنند (11).

کمر درد عمدتا به سه گروه مکانیکال ، غیر مکانیکال و سایکولوژیکال تقسیم می شود. عمده ی کمر دردها در گروه مکانیکال جای می گیرند و خودشان به دو گروه کمر درد های اختصاصی و غیر اختصاصی تقسیم می شوند. در نوع اختصاصی که در 5% افراد جامعه دیده می شود علت و منشا درد مشخص بوده در حالیکه در نوع غیر اختصاصی که 95% موارد کمر درد را به خود اختصاص می دهد علت و منشا مشخصی برای بروز علائم مشاهده نمی شود(12). در گروه کمر درد های اختصاصی علت درد میتواند فتق دیسک ، تنگی کانال نخاعی، سرخوردگی مهره های کمری ، آسیب مفاصل فاست ، بدخیمی ، بیماری های التهابی و عفونت باشد(13). همچنین از نظر تقسیم بندی بر اساس مدت زمان شروع علائم بیماران مبتلا به کمر درد به سه گروه حاد ( کمتر از 4هفته) ، تحت حاد (4 تا 12 هفته) و مزمن (بیشتر از 12 هفته) تقسیم می شوند (14).

## **اختلالات ایجاد شده در بیماران مبتلا به کمر درد غیر اختصاصی**

با توجه به شواهد، اختلالات متعددی در بیماران مبتلا به کمر درد مشاهده شده است . از جمله ی این اختلالات می توان به کاهش ضخامت وسایز عضله عرضی شکمی و مولتی فیدوس(15) (16) یا فعالیت بیشتر عضله ی راست شکمی حین نشستن روی یک سطح بی ثبات نسبت به عضله ی عرضی شکمی اشاره کرد (18) . اختلال دیگر، حرکات کنترل نشده ی ناحیه ی کمری- لگنی می باشد(19). بیماران مبتلا به کمر درد ، در ناحیه ی کمری-لگنی ممکن است دامنه ی حرکتی بیشتری نسبت به افراد سالم داشته باشند که این باعث تخریب بافت نرم ناحیه می شود . اگر این حرکت بیش از حد در یک جهت مکررا رخ دهد باعث میکروتروما و تشدید کمر درد می شود (20) . نقص دامنه حرکتی مفاصل اندام تحتانی نیز از جمله اختلالات ناحیه کمر می باشد(21). تحقیقات نشان داده است که در افراد مبتلا به کمر درد زاویه چرخش داخلی مفصل هیپ نسبت به افراد سالم کمتر است.این محدودیت در حرکت هیپ در حرکاتی که نیاز به دامنه ی کامل در جهت خاص تنه و هیپ دارد ، می تواند باعث تشدید کمر درد شود. همچنین در افراد مبتلا به کمر درد اختلال در موتورکنترل عضلات عمقی تنه (22) ، اختلالات کنترل تعادل مانند افزایش نوسانات وضعیتی(23) ، کاهش کنترل راستای وضعیتی^[1]^ به علت اختلال وتغییر در پردازش حس عمقی (24)و عدم تقارن تحمل وزن بین اندام های تحتانی (25) دیده شده است. با توجه به مطالعات مختلف ، در بیماران مبتلا به کمر درد تغییراتی در عضلات عمقی شکمی مخصوصا عرضی شکمی اختلال در عملکرد پیش بینی کننده ی عضله ی عرضی شکمی(26)) و نیز تغییراتی در فانکشن ( مثل ناتوانی در انقباض ) و مورفولوژی مولتی فیدوس (مثل سایز ، عدم تقارن ) یافت شده است. تعدادی از مطالعات نشان داده اند که فعالیت اتوماتیک عضله ی عرضی شکمی حین ایجاد اغتشاش در بیماران مبتلا به کمر درد کاهش یافته است و این می تواند روی مکانیسم های محافظتی پیش بینی کننده کنترل ستون فقرات اثر بگذارد(27) .

## **اختلالات مرتبط با اندازه و ضخامت عضلات شکمی و مولتی فیدوس در بیماران مبتلا به کمر درد مزمن غیر اختصاصی**

عضلات شکمی شامل عضله عرضی شکمی^[2]^، مایل داخلی ^[3]^،ومایل خارجی^[4]^ در ثبات و جهت گیری^[5]^ ستون فقرات ، و برقراری تعادل بین نیروهای داخلی و خارجی حین فعالیت های فانکشنال مشارکت دارند(28). عضلات عمقی نظیر عرضی شکمی و بخش خلفی مایل داخلی با اتصال به فاشیای سینه ای –کمری ^[6]^ همراه با عضلات کف لگن و دیافراگم باعث افزایش ثبات در ستون فقرات می شوند (29) . عضله ی مهم دیگر در ستون فقرات، عضله مولتی فیدوس می باشد که نقش مهمی در ایجاد ثبات ستون فقرات ، ثبات داینامیک ناحیه ی کمری-لگنی و حس عمقی ستون فقرات کمری دارد(30). مطالعات اولتراسونوگرافی نشان می دهد که ضخامت عضله ی عرضی شکمی که یک عضله ی ثبات دهنده ستون فقرات می باشد در بیماران مبتلا به کمردرد نسبت به افراد سالم کاهش یافته است و افزایش فعالیت رکتوس شکمی در بیماران مبتلا به کمر درد مشاهده است(31). همچنین در یک مطالعه که تقارن سطح مقطع عضله ی مولتی فیدوس کمری بررسی شده است ؛ بیماران با کمر درد یک طرفه نسبت به افراد با کمر درد دو طرفه در سمتی که درد بیشتری داشته اند کاهش ضخامت شدیدتری از عضله ی مولتی فیدوس را در سطح مهره های چهارم و پنجم کمری نشان داده اند ؛ یعنی بیشتر بودن عدم تقارن ممکن است با سطح بالاتری از شدت درد همراه باشد(32).

متد های مختلف مثل الکترومیوگرافی ، MRI و اولتراسونوگرافی برای ارزیابی ویژگی های مختلف عضلات شکمی و کمری و بافت های اطراف به ویژه چربی در افراد سالم و کمر درد استفاده می شود. از بین همه ی این ها اولتراسونوگرافی ابزار مناسبی برای مشاهده ی الگوی انقباض عضلات است چون به شناسایی بافت های انقباضی عمقی اجازه می دهد و در عین حال نسبت به MRI کم هزینه تر و در کل بدون ضرر و غیر تهاجمی است و مطابق شواهد پایایی^[7]^ آن 0.958 ICC=0.859 - گزارش شده است(33). محاسبه ی ضخامت عضلات شکمی و مولتی فیدوس در پوزیشن های مختلف خوابیده روی تخت ، نشسته و ایستاده بررسی شده است(34) . مطابق این شواهد بیشترین پایایی برای عضله ی عرضی شکمی در وضعیت خوابیده بوده است (ICC=0.90 ). هم چنین برای مولتی فیدوس در وضعیت خوابیده و نشسته پایایی قابل قبول گزارش شده است(34)

## **اختلالات مرتبط با تغییرات ایجاد شده در سیستم عصبی مرکزی بیماران مبتلا به کمردرد مزمن غیر اختصاصی**

مطالعات اخیر در بیماران مبتلا به کمر درد مزمن افزایش تحریک پذیری سیستم اعصاب مرکزی و آتروفی بافت مغز را نشان داده است. آتروفی بافت مغز به صورت کاهش تراکم ماده ی خاکستری در مناطقی که با شدت درد و مدت زمان آن ارتباط دارد شامل کورتکس پره فرونتال و سوماتوسنسوری ، تالاموس و ساقه ی مغز (35) مشاهده شده است. همچنین دیده شده است که در افراد با کمر درد مزمن غیر اختصاصی میزان دردی که در پاسخ به تحریک بافت های محیطی دور از محل اولیه ی علائم داشته اند بسیار بیشتر از شدت متحرک بوده است . هرچند ممکن است در این افراد تغییراتی در عضلات ، لیگامنت ها و مفاصل رخ داده باشد اما شدت این اختلالات ساختاری ارتباط ضعیفی با تظاهرات بالینی داشته است(36). یک مکانیسم برای توجیه این پدیده، حساس شدگی مرکزی ^[8]^ است که در بیماران با کمر درد مزمن دیده شده است و به معنی تقویت سیگنال های عصبی درون سیستم عصبی مرکزی است که حساسیت به درد را افزایش می دهد. یکی از مکانیسم های نزولی مهاری که درد را تنظیم می کند کنترل مهاری مضطرب منتشر ^[9]^است(37). در حیوانات فعالیت نورون های nociceptive شاخ خلفی در پاسخ به محرک های ناخوشایند^[10]^ اعمال شده کاهش یافته است. در انسان این شرایط تحت عنوان شرایط تنظیم درد(CPM)^[11]^ نامیده می شود که باعث افزایش آستانه ی درد خارج از محل محرک ناخوشایند می شود. فعالیت CPM باعث کاهش فعالیت نورونی شاخ خلفی نخاع ، کاهش درد و hypoalgesia می شود. در یک مطالعه ی مروری سیستماتیک اثر CPMبین افراد با درد مزمن وافراد سالم مقایسه شده است و به این نتیجه رسیده اند که CPM در افراد با درد مزمن مختل شده است(38). همچنین با افزایش سن CPM کاهش یافته و این اختلال در زنان با درد مزمن بیشتر از مردان است(38). CPM با استفاده از آستانه ی فشاری درد ^[12]^ به عنوان تست تحریکی ارزیابی می شود. دیده شده است که افراد با درد مزمن شناخت نامناسبی از درد دارند که این مرتبط با عواملی مثل ترس از حرکت و فاجعه انگاری درد ^[13]^ می باشد(39).

## **مداخلات مرتبط با اختلالات موجود در بیماران مبتلا به کمر درد مزمن غیر اختصاصی**

مداخلات غیر دارویی مختلف مثل درمان های دستی(موبیلیزیشن و منیپولیشن) ، تمرین درمانی(تمرینات ثباتی ، قدرتی ، تعادلی) ، الکتروتراپی ، ماساژ و درمان های شناختی رفتاری ^[14]^ برای درمان بیماران مبتلا به کمر درد به کار گرفته شده است. در رابطه با درمان کمر درد مزمن تمرین درمانی می تواند در کاهش درد ، بهبود ناتوانی ، بهبود قدرت اکستنشن پشت ، موبیلیتی و استقامت موثر باشد(40). انواع تمرین درمانی در این بیماران شامل تمرینات هوازی، تمرینات تقویتی ،انعطاف پذیری ،تمرینات کششی ، تمرینات ثباتی ، تمرینات موتور کنترل ، تمرینات تعادلی ، تمرینات مرکزی و تمرینات فلکشن کمر می باشد. هر چند که مطالعات قبلی به طور قطعی برتری یک نوع از تمرین درمانی را نسبت به انواع دیگر نشان نداده است ، در یک مطالعه ی مروری دیده شده است که در بیماران مبتلا به کمر درد غیر اختصاصی ترکیب تمرینات ثباتی با تمرینات جنرال باعث کاهش ناتوانی در مدت کوتاه تری نسبت به تمرینات جنرال به تنهایی شده است(41).

## **تمرینات ثباتی**

یکی از انواع تمرینات مطرح شده برای بیماران مبتلا به کمر درد مزمن غیر اختصاصی تمرینات ثباتی می باشد. با توجه به اینکه در بیماران مبتلا به کمر درد عملکرد عضلات عمقی ستون فقرات مثل عرضی شکمی و مولتی فیدوس دچار اختلال شده اند این تمرینات با اثر گذاری بر کنترل حرکت عضلات می تواند باعث کاهش درد و ناتوانی و بهبود فانکشن در بیماران مبتلا به کمر درد شود(42). هدف تمرینات ثباتی تنه بهبود هماهنگی عضلات سینرژیست و آنتاگونیست و به حداقل رساندن حرکات غیر ضروری در ناحیه ی کمری- لگنی برای حفاظت از تنه از میکروتروما ، درد و تغییرات تخریبی می باشد(43) . مطالعات قبلی نشان داده اند که تمرینات ثباتی باعث بهبود ضخامت عضلات شکمی( مثل عضله ی عرضی شکمی ) شده اند (44). تمرینات ثباتی بر مبنای استفاده از مانور abdominal drawing-in به طور گسترده برای انقباض عضله ی عرضی شکمی به کار گرفته می شود(45). این متد با استفاده از انقباضات ایزومتریک ، عضلات عمقی تنه را به کار میگیرد (46)، انقباضات عضلات گلوبال را به حداقل می رساند و به صورت انتخابی باعث انقباض عضله ی عرضی شکمی می شود(47). به علاوه این تمرینات تنشن را در فاسیای سینه ای – شکمی افزایش می دهد و باعث افزایش ثبات در ناحیه ی کمری – لگنی می شود(48). برخی از مطالعات اخیر در بیماران مبتلا به کمر درد تمرینات را همراه با ویبریشن برای بیماران در نظر گرفته اند. از میان متد های مختلفی که برای ایجاد ویبریشن استفاده میشود، می توان به فلکسی بار اشاره کرد. این ابزار شامل یک سری وزنه در انتهای یک نوار الاستیک به طول تقریبی 152 سانتی متر می باشد و با فرکانس 5 هرتز باعث ایجاد ویبریشن می شود(49). ویبریشن هایی که توسط فلکسی بار ایجاد می شود به وسیله ی تحریک حس عمقی باعث بهبود درک حرکت می شود(50). تمرینات با فلکسی بار باعث افزایش فعالیت عضلات تنه و افزایش فعالیت عضلات شکمی و عضلات اطراف ستون فقرات می شود بنابراین مطالعات اخیر که تمرینات را با استفاده از فلکسی بار داده اند بر روی فعالیت عضلات ثبات دهنده ی تنه مثل عرضی شکمی و مولتی فیدوس تمرکز داشته اند(51). همچنین  بر اساس مقالات این تحریکات ارتعاشی علاوه بر تحریک رسپتورهای ارتعاشی و کاهش درد می تواند باعث فعال شدن دوک های عضلانی، افزایش فعالیت آلفا موتور نورون ها و تقویت کنترل عصبی-عضلانی در عضلات تنه شود (52). از این ابزار در مراکز مختلف درمانی در پوزیشن های مختلف برای بهبود قدرت ، هماهنگی و تعادل استفاده می شود(51).

## **Pain Neuroscience Education( PNE)**

استراتژی های مختلفی که برای درمان بیماران مبتلا به کمر درد به کار گرفته شده است به نظر می رسد که شناخت درد ، جنبه های رفتاری و دانش فیزیولوژی درد را نادیده گرفته است. مکانیسم های درد به طور گسترده به سه دسته تقسیم بندی می شوند: 1) مکانیسم های ورودی شامل درد مضطرب ^[15]^و درد های محیطی با منشا عصبی 2) مکانیسم های پردازشی ^[16]^شامل درد مرکزی و حساس شدگی مرکزی و مکانیسم های شناختی-عاطفی درد 3) مکانیسم های خروجی شامل سیستم های اتونومیک، حرکتی ، هورمونی-عصبی و ایمنی می باشد(53). عمده ی مداخلات اخیر روی مکانیسم های ورودی و مکانیسم های خروجی تمرکز دارند و توجه کمتری به مکانیسم های مرکزی داشته اند(54). یکی از رویکرد های درمانی که اخیرادر بیماران با کمر درد مزمن به کار گرفته می شود PNE می باشد. این رویکرد بر آموزش بیماران درباره ی مکانیسم های مرتبط با درد مزمن مثل درد مرکزی یا حساس شدگی مرکزی و مکانیسم های شناختی-عاطفی درد ؛ تمرکز دارد و ممکن است به بازگشت تدریجی بیماران به فعالیت هایی که برای انجام آن ترس دارند کمک کند(54). PNE با آموزش های سنتی درباره ی درد تفاوت دارد و هدف آن کاهش حساسیت سیستم عصبی با تمرکز بر ارائه ی نوروفیزیولوژی ، نوروبیولوژی درد و معنای آن می باشد در حالیکه آموزش های سنتی بیشتر روی مدل های آناتومیکی و بیومدیکال تمرکز دارند(55). مطالعات اخیر نشان داده اند که PNE باعث تغییر در شدت درد، دانش درد، ناتوانی ، عملکرد های روانشناختی و رفتارهای مرتبط با درد در بیماران با درد های عضلانی اسکلتی شده است(56). البته لازم به ذکر است که PNE به تنهایی روی کاهش نمره ی درد اثری ندارد. در PNE با رویکردی مواجه هستیم تحت عنوان time-contigent ( انجام دادن چند تکرار از یک تمرین بدون توجه به درد) در مقابل رویکرد symptom-contigent (توقف تمرین درمانی با شروع درد)(57) . بر اساس یک مطالعه ی گزارش موردی دیده شده است که درمان های شناختی رفتاری در افراد مبتلا به کمر درد باعث بهبودی درد و ناتوانی با مشاهده ی تغییرات در استقامت عضلات تنه ، کینماتیک تنه و هیپ و لگن و انعطاف پذیری ستون فقرات کمری شده است و نیز باعث تغییر در استراتژی حرکت و بهبود توزیع لود و فشار ، کاهش ترس از اعمال لود در ناحیه ی کمری فرد شده است(59).

# **مروری بر پژوهش های قبلی**

در این قسمت به مروری بر مطالعات انجام شده در سه بخش پرداخته شده است

**الف) مطالعاتی که در زمینه ی اثر تمرینات ثباتی در بیماران مبتلا به کمر درد مزمن غیر اختصاصی انجام شده اند.**

نور محمد پور و همکاران در سال 2018 مطالعه ای با هدف بررسی اثر تمرینات ثباتی مرکزی در درمان پرستاران مبتلا به کمر درد مزمن غیر اختصاصی انجام داده اند(60). 36 پرستار زن مبتلا به کمر درد مزمن در دو گروه مداخله و درمان تقسیم شدند. متغیر های درد، ناتوانی ، کیفیت زندگی ، و ضخامت عضلات شکمی(عرضی شکمی مایل داخلی ، مایل خارجی ) مورد بررسی قرار گرفت. پروتکل تمرین درمانی در گروه مداخله 8 هفته تمرین درمانی که هر هفته دو تمرین با توپ و دو تمرین روی زمین بوده است. تمرینات شامل مانور ADIM در حالت های خوابیده ، چهار دست و پا و نشسته روی زمین و توپ سوئیسی بوده است . گروه کنترل هیچ مداخله ای دریافت نکرده اند. نتایج این مطالعه بهبودی معناداری در درد ، ناتوانی ، کیفیت زندگی و ضخامت عضلات شکمی در گروه مداخله نسبت به کنترل نشان داده است

Kwang-Jun Ko و همکاران در سال 2018 مطالعه ای با هدف بررسی اثر 12 هفته تمرین ثباتی کمر و تمرین اسلینگ بر روی زاویه ی لومبوساکرال ، قدرت عضلات ناحیه ی کمری و درد در بیماران مبتلا به کمر درد مزمن غیر اختصاصی انجام داده اند(61). 29 زن مبتلا به کمر درد در سه گروه تمرینات ثباتی ، اسلینگ و کنترل تقسیم شده اند. هر دو گروه تمرین درمانی تمرینات را به مدت 60 دقیقه سه بار در هفته در 12 هفته انجام داده اند. متغیر های زاویه ی لومبوساکرال ، قدرت عضلات کمر و درد بررسی شده اند. تمرینات اصلی در هر دو گروه شامل sit up, 2) superman exercise, 3) quadruped arm & leg raise, 4) squat, 5) lower body fixation plank (surface or sling), 6) upper body fixation plank (surface or sling), 7) side plank (surface or sling), and 8) hip bridge (surface or sling). بوده است. هر دو گروه تمرینات ثباتی و اسلینگ موارد 1 تا 4 را روی زمین انجام داده اند. گروه تمرینات ثباتی مورد 5 تا 8 را روی زمین انجام داده و گروه اسلینگ 5 تا 8 را با اسلینگ انجام داده است. نتایج این مطالعه نشان داد که گروه های تمرینات ثباتی و اسلینگ هیچ یک روی زاویه ی لومبوساکرال اثری نداشته اند در حالیکه قدرت عضلات فلکسور و اکستنسور کمر و شدت درد در هر دو گروه در مقایسه با گروه کنترل بهبودی معناداری داشته است.

Cho-Rong Bae و همکاران در سال 2018 مطالعه ای با هدف مقایسه ی تمرین sit-up و تمرین ثباتی مرکزی در بیماران مبتلا به کمر درد مزمن غیر اختصاصی انجام داده اند(62). 36 بیمار مبتلا به کمر درد در 2گروه تقسیم شدند. متغیر های درد ، ناتوانی ، ضخامت عضلات شکمی با استفاده از اولتراسونوگرافی و فعالیت عضلات شکمی با استفاده از الکترومیوگرافی در مطالعه مورد بررسی قرار گرفته است. در گروه تمرین ثباتی افراد تمرینات ADIM ، SLR در حالت های سوپاین و پرون ، بلند کردن دست و پای مخالف در حالت چهار دست و پا را به مدت 12 جلسه انجام داده اند. در گروه sit-up	تمرین کمکی sit-up	با استفاده از HubEX-LEX(نوعی ماشین برای انجام تمرین sit-up ) انجام شده است. نتایج این مطالعه نشان داد که نسبت ضخامت ( انقباض/استراحت) عضلات راست شکمی و مایل خارجی در گروه تمرین sit-up	و عرضی شکمی در گروه تمرینات ثباتی مرکزی قبل و بعد از درمان تفاوت معناداری داشته است. نسبت فعالیت عضله ی مایل داخلی به عرضی شکمی و درد و ناتوانی در هر دو گروه بهبودی معناداری نشان داده است.

قیطاسی و همکاران در سال 2020 مطالعه ای با هدف بررسی اثر تمرینات ثباتی مرکزی در ترکیب با تمرینات حسی حرکتی بر روی درد ، عملکرد و ترس از حرکت در ورزشکاران مبتلا به کمردرد مزمن غیر اختصاصی انجام داده اند(63). 30 ورزشکار مرد مبتلا به کمر درد در دو گروه مداخله و کنترل تقسیم بندی شده اند. درد ، عملکرد و ترس از حرکت بررسی شده اند. گروه مداخله 8 هفته تمرین ثباتی(1 داخل بردن شکم در وضعیت دمر ، ۲ وضعیت چهار دست و پا همراه با بالا آوردن دست و پای مخالف ۳ پل یک طرفه ی تعدیل شده به پهلو برای هر سمت بدن ۴ خوابیدن طاقباز بر روی توپ سوئیسی همراه با انقباض استاتیک عضلات شکم ۵ بالا آوردن دست و پای مخالف بر روی توپ سوئیسی ۶ ایستادن روی یک پا با زانوی خم ۷ ایستادن روی دو پا در وضعیت آناتومیکی با چشمان بسته ۸ قرار گرفتن با دوپا روی تخته تعادل ۹ استفاده از صندلهای تعادل روی دو پا ۱۰ گام برداشتن با استفاده از صندلهای تعادل ۱۱ استفاده از صندلهای تعادل با زانوی خم روی دو پا) در ترکیب با تمرینات حسی حرکتی انجام داده اند و در گروه کنترل هیچ مداخله ی درمانی انجام نشده است. نتایج این مطالعه نشان داد که گروه مداخله در مقایسه با گروه کنترل بهبودی معناداری در درد ، عملکرد و ترس از حرکت داشته اند.

Sin Ho Chung و همکاران در سال 2018 مطالعه ای با هدف تعیین اثر تمرینات ثباتی کمر با استفاده از فلکسی بار روی درد ، ناتوانی ، ظرفیت فعالیت و ضخامت عضله ی عرضی شکمی در بیماران مبتلا به کمر درد مزمن غیر اختصاصی انجام دادند(64).27 بیمار در دو گروه آزمایش و کنترل تقسیم شدند. متغیر های مطالعه شامل درد (VAS) ، ناتوانی (ODI) ، ظرفیت فعالیت عضله ی عرضی شکمی( با استفاده از واحد فشاری بیوفیدبک) و ضخامت عضله ی عرضی شکمی ( اولتراسونوگرافی) بوده است. در هر دو گروه تمرینات 30 دقیقه در روز ، سه بار در هفته به مدت 6 هفته انجام شده است. در گروه آزمایش مانور ADIM در حالات ایستاده ، خوابیده به پشت و دمر و چهار دست و پا به مدت 10 ثانیه انجام شده است. در این وضعیت افراد از هر دو دست برای نگه داشتن فلکسی بار استفاده کرده اند( در وضعیت چهار دست و پا متناوبا هر دست فلکس بار را نگه داشته است). هر حرکت 10 بار در ست هایی با 3 تکرار انجام شده است. فلکسی بار با فرکانس 4.6 هرتز ویبریشن ایجاد کرده است. در گروه کنترل تمرین های مشابه ی گروه آزمایش بدون استفاده از فلکسی بار به بیماران داده شده است. نتایج این مطالعه نشان داد که تمامی متغیر ها در هر دو گروه بعد از 6 هفته تمرین درمانی بهبودی داشته اند و این بهبودی در گروه آزمایش برای تمامی متغیر ها به جز ضخامت عضله ی عرضی شکمی بیشتر بوده است.

Yue-mei SUN و همکاران در سال 2021 مطالعه ای با هدف بررسی اثر فلکسی بار در بیماران با کمر درد مزمن غیر اختصاصی انجام دادند(65). 30 بیمار مبتلا به کمر درد وارد مطالعه شدند. متغیر های مورد بررسی در مطالعه درد(VAS) ، ناتوانی (ODI) ، ضخامت عضله ی عرضی شکمی و مساحت مولتی فیدوس و تفاوت در مقادیر RMS الکترومیوگرافی عضله ی مولتی فیدوس و عرضی شکمی بوده است. در گروه آزمایش، تمرینات ثباتی مرکزی(supine bridge, curl-up and four-point support) همراه با فلکسی بار به بیماران داده شده است و گروه کنترل تمرینات را بدون فلکسی بار انجام داده اند. هر دو گروه تمرینات را 3 بار در هفته به مدت 6 هفته انجام داده اند. نتایج این مطالعه نشان داد که تفاوت معناداری در RMS عضله ی مولتی فیدوس و عرضی شکمی بین دو گروه بوده است . بعد از تمرین درمانی در هر دو گروه مقادیر درد و ناتوانی به طور معناداری کاهش یافت و ضخامت و مساحت مولتی فیدوس به طور معناداری افزایش یافته است و این افزایش مقادیر در همه ی متغیر ها به جز ضخامت عضله ی عرضی شکمی در گروه آزمایش از گروه کنترل بیشتر بوده است.

**نتیجه گیری کلی:** تمرینات ثباتی در بیماران مبتلا به کمر درد غیر اختصاصی موجب کاهش درد ، ناتوانی ، بهبود ضخامت عضلات شکمی و ترس از حرکت شده است . همچنین فلکسی بار در بهبود درد و ناتوانی همراه با تمرینات ثباتی در بیماران مبتلا به کمر درد غیر اختصاصی مفید بوده است اما اثر آن روی فعالیت و ضخامت عضلات تنه نیاز به بررسی های بیشتری دارد.

**ب)مطالعاتی که در زمینه ی بررسی اثر PNE در بیماران مبتلا به کمر درد مزمن غیر اختصاصی انجام شده اند.**

Adriaan Louw و همکاران در سال 2016 مطالعه ی مروری سیستماتیک به هدف بررسی اثر PNE در بیماران با درد عضلانی اسکلتی مزمن انجام دادند(66). سرچ سیستماتیک در 11 پایگاه داده انجام شده است. همه ی RCT هایی که اثر PNE را در درد عضلانی اسکلتی مزمن( کمر درد ، گردن درد و فیبرومیالژیا) بررسی کرده اند از سال 2002 تا 2016وارد مطالعه ی مروری شده اند.. افرادی که در مطالعات وارد شده اند 18 سال به بالا بوده اند و متغیر های درد ، فانکشن ، فاکتورهای روانی اجتماعی، حرکت و استفاده از مراقبت های بهداشتی^[17]^ بررسی شده اند. موارد مرتبط با PNE که در مطالعات بررسی شده اند شامل : نوروفیزیولوژی درد ، مسیر های Nociception and nociceptive ، سیناپس ها، پتانسیل عمل ها، تسهیل ها و مهار های نخاعی، حساس شدگی محیطی و مرکزی، شکل پذیری^[18]^ سیستم عصبی ، فاکتور های فیزیولوژیکال و باور های مرتبط با درد بوده است . طولانی ترین دوره ی آموزش 4 ساعت در یک جلسه و کوتاه ترین 30 دقیقه بوده است. مداخلاتی که در مطالعات همراه آموزش ها به بیماران داده شده است شامل: موبیلیزیشن و منیپولیشن ، ماساژ بافت نرم ، متحرک سازی بافت عصبی و عضله ، تمرینات ثباتی تنه ، تمرینات هوازی مداری، سوزن خشک ، تمرینات ثباتی گردن وآب درمانی بوده ویا اموزش علوم اعصاب به تنهایی به کار برده شده است . نتایج این مطالعه مروری نشان داد که PNE باعث بهبود درد ، ناتوانی ، ترس از حرکت ، بهبودی در فلکشن تنه و SLR و عملکرد فیزیکی در بیماران مبتلا به کمر درد شده است.

Anneleen Malfliet و همکاران در سال 2018 مطالعه ای با هدف مقایسه ی اثر PNE در ترکیب با تمرینات کنترل حرکتی به هدف شناختی^[19]^ برای کاهش درد ، بهبود فانکشن و مشخصات متدلوژیک ماده ی خاکستری مغز و شناخت درد در بیماران با درد مزمن ستون فقرات انجام دادند(67). 120 نفر (18 تا 65 سال)بیمار مبتلا به درد مزمن غیر اختصاصی ستون فقرات وارد مطالعه شدند و سپس در دو گروه آزمایش( ترکیب PNE با تمرینات کنترل حرکتی به هدف شناختی ) و گروه کنترل( ترکیب آموزش های سنتی درباره درد کمر و گردن و تمرینات جنرال) قرار گرفتند. متغیر هایی که در این مطالعه بررسی شدند شامل : آستانه ی فشاری درد، مقیاس عددی درد^[20]^ ، حساس شدگی مرکزی، مشخصات متدلوژیک ماده ی خاکستری(با استفاده از MRI) شناخت درد ( فاجعه سازی درد ، ترس از حرکت) و نیز ناتوانی ، سلامت ذهنی و فیزیکی بودند. هردو گروه مداخله شامل سه جلسه ی آموزشی (آموزش گروهی ، اموزش آنلاین در خانه و آموزش فردی) و 15 جلسه تمرین یک به یک بوده است. در گروه آزمایش به افراد آموزش هایی درباره ی باورهای مرتبط با درد ، افزایش شناخت درد و کاهش تهدید های آن داده شده است. محتوای این آموزش ها درباره ی نورون، سیناپس ، مسیرهای nociceptive تسهیلی و مهاری نزولی و حساس شدگی های محیطی و مرکزی بوده است. برنامه ی تمرینی به همراه تمرینات کنترل حسی حرکتی به هدف تغییر عقاید و درک نامناسب به سمت تصحیح شده ی آن شروع شده است. همزمان حرکاتی که افراد برای انجام آن ترس داشته اند و از انجامش اجتناب می کردند با استفاده از افزایش تدریجی پیچیدگی آن حرکت به فرد معرفی شده است. گروه کنترل شامل آموزش های سنتی مثل آموزش علت های مکانیکال درد گردن و کمر، آناتومی ستون فقرات ، فیزیولوژی وبیومکانیک، اهمیت مراقبت از خود و ارگونومی ؛ نیروهای مفصلی و فشار بین دیسک ها ، تکنیک های بلند کردن اجسام و مدت تمرینات استقامتی ، کششی و تقویتی بوده است. برنامه ی تمرینی این گروه نیز روی درمان دیسفانکشن های بیومدیکال ستون فقرات ( مثل موبیلیتی، قدرت و استقامت عضله و تناسب عمومی بدن) با تکمیل به سمت فعالیت های فانکشنال و فعالیت های فیزیکی در حالی که ستون فقرات در وضعیت خنثی^[21]^ باشد ، تمرکز داشته است. نتیجه ی این مطالعه نشان داد که گروه آزمایش نسبت به کنترل بهبودی بیشتری در آستانه ی درد فشاری ، علائم حساس شدگی مرکزی، عملکرد های ذهنی و فیزیکی ،ترس از حرکت و کاهش ناتوانی داشته اند.

Ismail Saracoglu و همکاران در سال 2020 مطالعه ای با هدف بررسی اثر PNE در ترکیب با درمان های دستی^[22]^ و تمرینات در خانه بر درد ، عملکرد پشت ، ناتوانی و ترس از حرکت در بیماران با کمر درد مزمن انجام دادند(68). برای بررسی درد از NRS ، عملکرد پشت از Back Performance Scale ، ناتوانی از Oswestry Disability Index و ترس از حرکت از Tampa Scale استفاده شده است. 69 بیمار( 18 تا 65 سال) در سه گروه 1) PNEهمراه با درمان های دستی و تمرین در خانه 2) درمان های دستی همراه تمرینات در خانه 3) تمرینات در خانه(گروه کنترل) تقسیم بندی شدند. همه ی مداخلات چهار هفته زمان برده اند. در گروه اول برای بیماران ، تکنیک موبیلیزیشن خلفی-قدامی در وضعیت دمر، موبیلیزیشن همراه با حرکت برای مهره های تحتانی ستون فقرات و نیروهای چرخشی در حالت خوابیده به پهلوی راست و چپ انجام شده است. سپس هر هفته یک جلسه PNE به مدت 40 تا 45 دقیقه و در مجموع 4 جلسه انجام شده است. برنامه ی تمرین در خانه برای همه ی گروه ها شامل افزایش قدرت و انعطاف پذیری عضلات شکمی ، ارکتور اسپاین، گلوتئال ، کوادریسپس و همسترینگ بوده است. نتایج این مطالعه نشان داد که افراد در گروه درمان های دستی همراه PNE و تمرین خانه بهبودی بیشتری در درد و ترس از حرکت در مقایسه با گروه های دیگر نشان دادند. سطح ناتوانی در هر دو گروه یک و دو نسبت به کنترل بهبودی معناداری داشته است.

Ki-Sang Kim و همکاران در سال 2022 مطالعه ای با هدف بررسی اثر PNE در ترکیب با تمرینات ثباتی کمر بر روی قدرت و درد در بیماران با کمر درد مزمن غیر اختصاصی انجام دادند(69). متغیر های مطالعه درد ، قدرت ، انعطاف پذیری و شاخص اختلال فعالیت بوده است. 35 زن مبتلا به کمردرد مزمن غیر اختصاصی در دو گروه PNEهمراه تمرینات ثباتی و تمرینات ثباتی به تنهایی قرار گرفته اند. در گروه اول افراد در ابتدای جلسه 10 دقیقه PNE را دریافت کرده اند و سپس گرم کردن و تمرینات ثباتی به مدت 20 دقیقه ( کشش همسترینگ ، عضلات شکمی و کوادریسپس / curl up/ dead bug/ bridge/ superman) انجام شده است. گروه کنترل فقط 30 دقیقه تمرینات ثباتی را انجام داده اند.. نتایج این مطالعه نشان داد که گروه اول بهبودی معناداری در درد ، قدرت عضلات شکمی و کمر ، ترس از حرکت ، تست finger to floor و فاجعه سازی درد نسبت به گروه دوم نشان داده اند اما در مورد متغیرهای ناتوانی و تست تغیر یافته ی modified Schober test بین دو گروه تفاوت معناداری مشاهده نشده است.

**نتیجه گیری کلی** : آموزش های مرتبط با فیزیولوژی و نوروفیزیولوژی درد در بیماران با کمر درد مزمن غیر اختصاصی در ترکیب با مداخلات مختلف فیزیوتراپی مثل درمان های دستی و تمرین درمانی باعث بهبود درد ، ناتوانی ، خود کار آمدی ، آستانه ی فشاری درد ، حساس شدگی مرکزی، شناخت درد ( فاجعه سازی درد ، ترس از حرکت) و نیز سلامت ذهنی و فیزیکی شده اند. عمده ی متغیر های بررسی شده در این مطالعات ، متغیر های سایکولوژیک بوده اند و اثر این آموزش ها بر متغیرهای فیزیکال کمتر مورد توجه قرار گرفته است.

# **ضرورت انجام مطالعه**

در مطالعات اخیر تغییراتی در نواحی مرتبط ماده خاکستری بافت مغز بسته به مدت و شدت درد در بیماران با کمر درد مزمن دیده شده است. بررسی مطالعات گذشته نشان می دهد عمده ی مداخلات رایج فیزیوتراپی بیشتر بر روی مکانیسم های ورودی و خروجی درد تاکید داشته و کمتر به مکانیسم های مرکزی آن پرداخته است، لذا اعمال مداخلات مؤثر بر مكانيسم هاي مركزي ضروري بنظر مي رسد.با در نظر گرفتن اين موضوع، اهمیت پرداختن به آموزش های PNE نمایان می شود. این آموزش ها با هدف ارائه توضیحاتی قابل فهم برای بیماران در خصوص مکانیسم های مرتبط با درد مزمن ، حساس شدگی های مرکزی و محیطی و نوروفیزیولوژی درد در کاهش ترس از حرکت ، کاهش درد و ناتوانی (66)، افزایش خود کار آمدی (69) و دانش درد ، کاهش فاجعه انگاری درد و کاهش اضطراب و علائم افسردگی موثر بوده اند(66) با این حال PNE به تنهایی در بهبود علائم تاثیر چندانی نداشته و مطابق شواهد ترکیب آن با انواع دیگر مداخلات نظیر درمان های دستی و تمرینات مختلف، اثر بیشتری بر بهبود علائم افراد مبتلا به درد مزمن داشته است. PNE باعث افزایش انگیزه برای شرکت در تمرین درمانی می شود و با بازگشت افراد به فعالیت های معمول باعث می شود تا احساس ناتوانی کمتری داشته باشند. در واقع با بهبود ترس از حرکت و اگاهی به این امر که درد آن ها صرفا به علت آسیب بافتی نیست بلکه ناشی از تغییراتی در مکانیسم های مرکزی پردازش درد نیز می باشد ، افراد مشارکت فعال تری در برنامه ی تمرین درمانی دارند(69). همچنین دیده شده است که افراد مبتلا به کمر درد مزمن ستون فقرات را در وضعیتی قرار میدهند که کمترین میزان درد را داشته باشند. این الگوی حرکتی مداوم می تواند منجر به الگوی حرکتی ناهنجارشود. بنابرین این بیماران از کینزیوفوبیا رنج می برند .درمان های شناختی رفتاری می تواند باعث بهبود ترس از حرکت در این بیماران شود. نشان داده است که تغییر در ضخامت عضلات شکمی 18% توسط تغییر در میزان درد افراد و 82% توسط تغییر در دیگر فاکتورها مثل فاکتورهای سایکولوژیکال توضیح داده می شود. بنابراین محتمل است که تغییر در باورهای ترس اجتنابی می تواند باعث تغییر درفعالیت عضلات عمقی شکمی در بیماران مبتلا به کمر درد مزمن غیر اختصاصی شود.(70) در بیماران مبتلا به کمردرد تغییراتی در بافت چربی درون ماهیچه ای ^[23]^در عضلات پارا اسپاینال ایجاده شده است. یک مکانیسم احتمالی در این مورد در بیماران مبتلا به کمر درد مزمن تغییر در رفتارهای حرکتی خاص فردی ^[24]^عضلات پارا اسپاینال می باشد. رفتار اجتنابی^[25]^ مرتبط با فاکتورهای مرتبط با درد که در مهار عضلات مشارکت دارند و تغییر در رفتارهای حرکتی خاص فردی عضلات پاراسپاینال می باشد ، چون افرادی که شناخت منفی نسبت به درد دارند از انجام حرکاتی که باعث ایجاد درد در آنان می شود اجتناب می کنند . با توجه به نکات مطرح شده از آن جایی که بافت چربی درون ماهیچه ای با سطح مقطع عضلات دربیماران مبتلا به کمر درد مزمن در ارتباط است، پس مداخلاتی مانند PNE که اثر بخشی مثبتی بر روی رفتارهای اجتناب از درد دارند می تواند اثرات مثبتی روی کاهش نفوذ چربی ^[26]^و بهبود بافت چربی درون ماهیچه ای و سطح مقطع عضلات داشته باشند.(71)

از سوی دیگر با توجه به نتایج مطالعات اخیر ، در افراد مبتلا به کمر درد اختلالات متعدد عضلانی اسکلتی مشاهده شده است. از جمله ی این اختلالات،کاهش ژئومتری عضلات تنه نظیر عضلات عرضی شکم و مولتی فیدوس ، (18) و تغییر در الگوی به کار گیری عضلات ثبات دهنده ستون فقرات بوده که باعث کاهش ثبات این ستون می شود(16) . مطابق شواهد تاثير تمرینات مختلف نظیر تمرینات ثباتی ، موتور کنترل و تقویتی در بهبود ضخامت عضلات شکمی و مولتی فیدوس تاكنون مشخص شده است (72)همچنين براساس گزارشهای موجود، تمرینات ثباتی می تواند باعث کاهش درد و ناتوانی و بهبود فانکشن در بیماران مبتلا به کمر درد شود(42). هدف از این تمرینات، بهبود هماهنگی عضلات دخیل در ثبات ستون فقرات و به حداقل رساندن حرکات غیر ضروری در ناحیه ی کمری- لگنی برای حفاظت تنه در برابر میکروتروما ، درد و تغیرات تخریبی می باشد(73). برخی از مطالعات اخیر در بیماران مبتلا به کمر درد تمرینات را همراه با ویبریشن برای بیماران در نظر گرفته اند و نشان داده اند که افزودن این ارتعاشات به پروتکل تمرین درمانی بیماران مبتلا به کمر درد می تواند باعث بهبودی بیشتر در درد و ناتوانی شود(74) . استفاده از فلکسی بار یک روش ايجاد ويبريشن حين تمرينات می باشد. با توجه به اینکه فلکسی بار یک تحریک ایمن با شدت کم بوده و باعث افزایش فعالیت عضلات تنه شده و می تواند در پوزیشن های مختلف همراه با تمرینات ثباتی استفاده شود(64) ابزاری مناسب برای بهبود فعالیت عضلات بنظر می رسد.

با در نظر گرفتن موارد فوق از آنجا كه تا به حال مطالعه ای به بررسی اثرتمرینات ثباتی با استفاده از فلکسی بار همراه با PNE بر ژئومتری عضلات شکمی و مولتی فیدوس در وضعیت های مختلف در بیماران مبتلا به کمر درد غیر اختصاصی نپرداخته است ، بنابراین در مطالعه حاضر آموزش های PNE همراه با تمرینات ثباتی با استفاده از فلکسی بار در بیماران مبتلا به کمر درد به کار گرفته خواهد شد تا بررسی شود که آیا افزودن PNE به تمرینات ثباتی با استفاده از فلکسی بار می تواند باعث بهبودی متغیر های موردبررسی شامل درد ، ناتوانی و ژئومتری عضلات تنه شود

# **اهداف و فرضیات**

**الف) اهداف طرح:**

**1-هدف کلی(goal) :** مقایسه ی تاثیر تمرینات ثباتی با استفاده از فلکسی بار همراه با PNE با تمرینات ثباتی همراه با فلکسی بار به تنهایی بر روی درد ، ناتوانی و ژئومتری عضلات تنه در بیماران با کمر درد مزمن غیر اختصاصی

**2-اهداف اختصاصی(objective)**

1)تعیین و مقایسه ی شدت درد قبل ، بلافاصله و یک ماه بعد از درمان با استفاده از مقیاس NRS در گروه تمرینات ثباتی با استفاده از فلکسی بار به تنهایی و تمرینات ثباتی با استفاده از فلکسی بار همراه با PNE.

2) مقایسه تغييرات شدت درد با استفاده از مقياس NRS بلافاصله و یک ماه بعد از درمان درگروه های تمرینات ثباتی با استفاده از فلکسی بار همراه با PNE و تمرینات ثباتی با استفاده از فلکسی بار به تنهایی.

3)تعیین و مقایسه ی سطح ناتوانی قبل ، بلافاصله و یک ماه بعد از درمان با استفاده از پرسشنامه ی رولاند موریس در گروه تمرینات ثباتی با استفاده از فلکسی بار به تنهایی و تمرینات ثباتی با استفاده از فلکسی بار همراه با PNE .

4)مقایسه ی تغییرات سطح ناتوانی با استفاده از پرسشنامه ی رولاند موریس بلافاصله و یک ماه بعد از درمان در گروه های تمرینات ثباتی با استفاده از فلکسی بار همراه با PNE و تمرینات ثباتی با استفاده از فلکسی بار به تنهایی.

5) تعیین و مقایسه ضخامت عضلات عرضی شکمی، مایل شكمي داخلي، مایل شكمي خارجي و مولتي فيدوس هردوسمت، قبل و بعد از درمان درگروه تمرینات ثباتی با استفاده از فلکسی بار به تنهایی و تمرینات ثباتی با استفاده از فلکسی بار همراه با PNE .

6) مقایسه تغييرات ضخامت عضلات عرضی شکمی ، مایل شكمي داخلي، مایل شكمي خارجي و مولتي فيدوس هردوسمت، قبل و بعد از درمان درگروه های تمرینات ثباتی با استفاده از فلکسی بار همراه با PNE و تمرینات ثباتی با استفاده از فلکسی بار به تنهایی.

7) تعیین و مقایسه سطح مقطع عضله مولتي فيدوس هردوسمت، قبل و بعد از درمان درگروه تمرینات ثباتی با استفاده از فلکسی بار به تنهایی و تمرینات ثباتی با استفاده از فلکسی بار همراه با PNE.

8) مقایسه تغييرات سطح مقطع عضله مولتي فيدوس هردوسمت، بعد از درمان درگروه های تمرینات ثباتی با استفاده از فلکسی بار همراه با PNE و تمرینات ثباتی با استفاده از فلکسی بار به تنهایی.

9) تعیین و مقایسه ی شدت ترس از حرکت قبل ، بلافاصله و یک ماه بعد از درمان با استفاده از مقیاس ترس از حرکت تامپا در گروه تمرینات ثباتی با استفاده از فلکسی بار به تنهایی و تمرینات ثباتی با استفاده از فلکسی بار همراه PNE.

10) مقایسه تغييرات شدت ترس از حرکت با استفاده از مقیاس ترس از حرکت تامپا بلافاصله و یک ماه بعد از درمان درگروه های تمرینات ثباتی با استفاده از فلکسی بار همراه با PNE و تمرینات ثباتی با استفاده از فلکسی بار به تنهایی.

11) تعیین و مقایسه ی شدت فاجعه انگاری درد قبل ، بلافاصله و یک ماه بعد از درمان با استفاده از مقیاس فاجعه آمیزی درد در گروه تمرینات ثباتی با استفاده از فلکسی بار به تنهایی و تمرینات ثباتی با استفاده از فلکسی بار همراه PNE.

12) مقایسه تغييرات شدت فاجعه انگاری درد با استفاده از مقیاس فاجعه آمیزی درد بلافاصله و یک ماه بعد از درمان درگروه های تمرینات ثباتی با استفاده از فلکسی بار همراه با PNE و تمرینات ثباتی با استفاده از فلکسی بار به تنهایی.

13) تعیین و مقایسه ی شدت باورهای ترس اجتنابی قبل ، بلافاصله و یک ماه بعد از درمان با استفاده از پرسشنامه ی باورهای ترس اجتنابی در گروه تمرینات ثباتی با استفاده از فلکسی بار به تنهایی و تمرینات ثباتی با استفاده از فلکسی بار همراه با PNE.

14) مقایسه تغييرات شدت باورهای اجتنابی ترس با استفاده از پرسشنامه ی باورهای ترس اجتنابی بلافاصله و یک ماه بعد از درمان درگروه های تمرینات ثباتی با استفاده از فلکسی بار همراه با PNE و تمرینات ثباتی با استفاده از فلکسی بار به تنهایی.

**3)هدف کاربردی(Purpose):**

با توجه به اینکه PNE روشی ایمن، آسان و کم هزینه می باشد می تواند باعث انحراف فکری بیمار از تمرکز بر بافتهای آسیب دیده و اصلاح باورهای ذهنی او شود و درک جدیدی نسبت به تجربه قبلی درد در فرد ایجاد کند. در صورتی که همراه کردن این آموزشها با تمرینات ثباتی باعث بهبودی در درد ، ناتوانی و ژئومتری عضلات تنه شود، کاربرد آن می تواند بر کاهش هزینه های درمانی و به حداقل رساندن سطح مراقبت های بهداشتی ، ارتقاء سطح حرکتی بیمار ، بهبود کیفیت زندگی و بازگشت سریعتر وی به فعالیت های معمول و اجتماع تاثیر بسزایی داشته باشد.

**ب)فرضیات یا سوال های پژوهشی:**

1)شدت درد با استفاده از مقیاس NRS قبل ، بلافاصله و یک ماه بعد از درمان در گروه تمرینات ثباتی با استفاده از فلکسی بار به تنهایی و تمرینات ثباتی با استفاده از فلکسی بار همراه با PNE متفاوت است.

2)تغییرات میانگین شدت درد با استفاده از مقیاس NRS بلافاصله و یک ماه بعد از درمان در گروه های تمرینات ثباتی با استفاده از فلکسی بار همراه با PNE و تمرینات ثباتی با استفاده از فلکسی بار به تنهایی متفاوت است.

3)سطح ناتوانی با استفاده از پرسشنامه رولاند موریس قبل ، بلافاصله و یک ماه بعد از درمان در گروه تمرینات ثباتی با استفاده از فلکسی بار به تنهایی و تمرینات ثباتی با استفاده از فلکسی بار همراه با PNE متفاوت است.

4)تغییرات میانگین سطح ناتوانی با استفاده از پرسشنامه ی رولاند موریس بلافاصله و یک ماه بعد از درمان در گروه های تمرینات ثباتی با استفاده از فلکسی بار همراه با PNE و تمرینات ثباتی با استفاده از فلکسی بار به تنهایی متفاوت است.

5) تغييرات ضخامت عضلات عرضی شکمی، مایل شكمي داخلي، مایل شكمي خارجي و مولتي فيدوس هردوسمت، قبل و بعد از درمان درگروه تمرینات ثباتی با استفاده از فلکسی بار به تنهایی و تمرینات ثباتی با استفاده از فلکسی بار همراه با PNE متفاوت است.

6) تغييرات ضخامت عضلات عرضی شکمی، مایل شكمي داخلي، مایل شكمي خارجي و مولتي فيدوس هردوسمت، قبل و بعد از درمان درگروه های تمرینات ثباتی با استفاده از فلکسی بار همراه با PNE و تمرینات ثباتی با استفاده از فلکسی بار به تنهایی متفاوت است.

7) تغييرات سطح مقطع عضله مولتي فيدوس هردوسمت، قبل و بعد از درمان درگروه تمرینات ثباتی با استفاده از فلکسی بار به تنهایی و تمرینات ثباتی با استفاده از فلکسی بار همراه با PNE متفاوت است.

8)تغييرات سطح مقطع عضله ی مولتي فيدوس هردوسمت، قبل و بعد از درمان درگروههای تمرینات ثباتی با استفاده از فلکسی بار همراه با PNE و تمرینات ثباتی با استفاده از فلکسی بار به تنهایی متفاوت است.

9) شدت ترس از حرکت با استفاده از مقیاس ترس از حرکت تامپا قبل ، بلافاصله و یک ماه بعد از درمان در گروه تمرینات ثباتی با استفاده از فلکسی بار به تنهایی و تمرینات ثباتی با استفاده از فلکسی بار همراه با PNEمتفاوت است .

10) تغييرات ميانگين شدت ترس از حرکت با استفاده از مقیاس ترس از حرکت تامپا بلافاصله و یک ماه بعد از درمان درگروه های تمرینات ثباتی با استفاده از فلکسی بار همراه با PNE و تمرینات ثباتی با استفاده از فلکسی بار به تنهایی متفاوت است .

11) شدت فاجعه انگاری درد با استفاده از مقیاس فاجعه آمیزی درد قبل ، بلافاصله و یک ماه بعد از درمان در گروه تمرینات ثباتی با استفاده از فلکسی بار به تنهایی و تمرینات ثباتی با استفاده از فلکسی بار همراه با PNE متفاوت است .

12) تغييرات ميانگين شدت فاجعه انگاری درد با استفاده از مقیاس فاجعه آمیزی درد بلافلصه و یک ماه بعد از درمان درگروه های تمرینات ثباتی با استفاده از فلکسی بار همراه با PNE و تمرینات ثباتی با استفاده از فلکسی بار به تنهایی متفاوت است.

13) شدت باورهای ترس اجتنابی قبل ، بلافاصله و یک ماه بعد از درمان با استفاده از پرسشنامه باورهای ترس اجتنابی در گروه تمرینات ثباتی با استفاده از فلکسی بار به تنهایی و تمرینات ثباتی با استفاده از فلکسی بار همراه PNE متفاوت است.

14) تغييرات ميانگين شدت باورهای ترس اجتنابی با استفاده از پرسشنامه ی باورهای ترس اجتنابی بلافاصله و یک ماه بعد از درمان درگروه های تمرینات ثباتی با استفاده از فلکسی بار همراه با PNE و تمرینات ثباتی با استفاده از فلکسی بار به تنهایی متفاوت است .

# **روش اجرای طرح**

**الف) نوع مطالعه( لطفا بر اساس جدول پیوست اقدام گردد)**:

طراحی این مطالعه از نوع کار آزمایی بالینی تصادفی شده می باشد و اثر تمرینات ثباتی با استفاده از فلکسی بار همراه با PNE در مقایسه با تمرینات ثباتی با استفاده از فلکسی بار به تنهایی را در بیماران با کمر درد مزمن غیر اختصاصی مورد بررسی قرار میدهد.

**ب) نمونه گیری( حجم نمونه ، نحوه انتخاب نمونه ، تعریف نمونه با ذکر شرایط ورود یا حذف موارد نمونه از مطالعه)**

با توجه به مطالعه مشابه (75) با در نظر گرفتن 05/0= α (خطای نوع اول) و 99/0=β- 1(توان آزمون) تعداد 21 نفر برای هر گروه به عنوان حجم نمونه تعیین شد. که در نهایت با در نظر گرفتن ریزش 10 درصدی حداقل تعداد 23 نفر برای هر گروه در نظر گرفته شد.

$$\boldsymbol{n=}\frac{{\boldsymbol{(}\boldsymbol{Z}_{\boldsymbol{1-}\frac{\boldsymbol{\alpha}}{\boldsymbol{2}}}\boldsymbol{+}\boldsymbol{Z}_{\boldsymbol{1-\beta}}\boldsymbol{)}}^{\boldsymbol{2}}\boldsymbol{(}\boldsymbol{\sigma}_{\boldsymbol{1}}^{\boldsymbol{2}}\boldsymbol{+}\boldsymbol{\sigma}_{\boldsymbol{2}}^{\boldsymbol{2}}\boldsymbol{)}}{{\boldsymbol{(}\boldsymbol{\mu}_{\boldsymbol{1}}\boldsymbol{-}\boldsymbol{\mu}_{\boldsymbol{2}}\boldsymbol{)}}^{\boldsymbol{2}}}$$

$$\boldsymbol{\mu}_{\boldsymbol{1}}\boldsymbol{=26.5}$$

$$\boldsymbol{\sigma}_{\boldsymbol{1}}\boldsymbol{=16.07}$$

$\boldsymbol{\mu}_{\mathbf{2}}$**=46.7**

$\boldsymbol{\sigma}_{\boldsymbol{2}}$ **=13.4**

روش تصادفي سازی استفاده شده روش تصادفي بلوکي دو سویه کور بوده به این ترتيب که یكي از گروه ها را با حرف A و گروه بعد را با حرف B نشان داده و جایگشت های مختلف 2 حرف انگليسي AB را مي نویسيم و به آنها شماره 4-1 اختصاص مي دهيم 1) AB 2) BA 3) AA 4)BB درهربار تخصيص افراد به گروه ها یک عدد به تصادف بين 4-1 انتخاب شده و عدد انتخابي الگوی تخصيص افراد به گروه ها را مشخص مي کند. برای مثال اگر عدد 2 انتخاب شد یعني نفر اول به گروه B و نفر بعدی به گروه A تعلق دارند.

نمونه گیری از طریق نصب آگهی در درمانگاه ها و بیمارستان های تحت پوشش دانشگاه علوم پزشکی شیراز وبه روش ساده از میان افراد مبتلا به کمردرد مزمن غیراختصاصی که به این مراکز وکلینیک های فیزیوتراپی در سطح شهر شیرازمراجعه می کنند ،انجام خواهد شد.

**معیار های ورود**:

- گروه سنی بین 20 تا 50 سال در صورتی که حداقل 3 ماه منتهی به شرکت در مطالعه درد داشته باشند
- میانگین شدت درد بر اساس مقیاس NRS بین 7-3
- سطح ناتواني عملكردی بيماران براساس پرسشنامه ی ناتوانی رولاند موریس در صورتی که بیشتر از8 باشد وارد مطالعه مي شوند(76)

**معیار های خروج**:

- سابقه ی بدخیمی در ناحیه ی ستون فقرات کمری و متاستاز آن به هر ناحیه ای(69)
- عفونت های سیستمی یا موضعی که باعث ایجاد درد حاد و شدید در ناحیه ی کمر شده باشد (69)
- سابقه ی بیماری های التهابی و سیستمیک مثل دیابت ، روماتوئید آرتریت(77)
- وجود دفورمیتی های ساختاری ستون فقرات (78)
- سابقه ی بیماری های نورولوژی و اسیب مغزی(77)
- سابقه ی جراحی های اندام های تحتانی ، شکمی و کمری (68)
- حاملگی (79)
- سابقه درمان های فيزیوتراپي و شرکت در تمرینات ورزشي منظم در یک ماه گذشته(80)
- اختلالات عضلانی اسکلتی و ارتوپدی اندام های فوقانی و تحتانی(81)

**ج) ابزار و فرآیند جمع آوری اطلاعات( یک نمونه از فرم جمع آوری اطلاعات ضمیمه گردد):**

1- مقیاس NRS

NRS (82)محتوی مجموعه ای از اعداد است که نشان دهنده ی کل دامنه ی محتمل برای شدت درد می باشد. عموما بیماران درد خود را از 0 تا 10 رتبه بندی میکنند. صفر نشانه ی نبود درد و 10 نشانه ی بیشترین شدت درد می باشد. در این مطالعه ، افراد با شدت درد حداقل 3 و حداکثر7 وارد مطالعه شده و برای هریک میانگین درد در هفته ی اخیر ثبت خواهد شد.

2- پرسشنامه ی Roland-Morris Questionnaire

پرسشنامه ی رولاند موریس برای بررسی سطح ناتوانی در بیماران مبتلا به کمر درد استفاده می شود. این پرسشنامه شامل 24 سوال بوده که به هر سوال از صفر تا 24 نمره دهی می شود. نمره ی صفر به معنی عدم ناتوانی و 24 به معنی حداکثر ناتوانی می باشد. مطابق شواهد پایایی ورژن فارسی این پرسشنامه(ICC^[27]^=0.86 ) گزارش شده است(83)

3 پرسشنامه ی Tampa scale of kinesiophobia

پرسشنامه ی ترس از حرکت محتوی 17 آیتم بر مبنای ارزیابی ترس از حرکت ،ترس از فعالیت فیزیکی و ترس اجتنابی طراحی شده است. این پرسشنامه برای اولین بار برای تمایز بین ترس بیش از حد و فوبیا در بیماران مبتلا به درد مزمن عضلانی اسکلتی ایجاد شده است. این پرسشنامه بر مبنای مدل fear-avoidance ، ترس از فعالیت های مرتبط با کار ، ترس از حرکت و ترس از آسیب را ارزیابی میکند. پرسشنامه دارای دو زیرمجموعه می باشد : 1) Activity avoidance= منعکس کننده ی فعالیت هایی که ممکن است باعث افزایش درد یا آسیب شود. 2) Somatic focus (SF)= منعکس کننده باورها و شرایط اساسی جدی. به هر آیتم پرسشنامه از 1 تا 4 امتیاز داده می شود. حداقل نمره ی کل 17 به معنی نبود ترس از حرکت وحداکثر 68 نشانه ی بیشترین مقدار ترس از حرکت می باشد و نمره ی ± 37 نشانه ی وجود ترس از حرکت می باشد. بر اساس شواهد ICC ورژن فارسی این پرسشنامه 0.86 می باشد(84)

4- پرسشنامه ی pain catastrophizing scale

فاجعه انگاری درد به معنی تمایل برای بزرگ سازی شدت درد و همچنین ناتوانی نسبی در مهار افکار مرتبط با درد می باشد. مقیاس فاجعه آمیزی درد با پرسیدن درباره ی اینکه افراد چگونه درد را حس میکنند ، افکاری که درباره ی درد خود دارند و زمانی که درد دارند، به افراد برای کمی سازی شدت درد کمک میکند. این پرسشنامه دارای 13 سوال می باشد و برای پاسخ به هر سوال از 0 تا 4 توسط فرد نمره داده می شود. مطابق شواهد پایایی ورژن فارسی این پرسشنامه مقدار Cronbach’s alpha=0.93گزارش شده است(85)

5- پرسشنامه ی Fear avoidance beliefs questionnaire

ترس از درد و اسیب دوباره و باورهای اجتنابی اثر عمده ای بر احساس ناتوانی ناشی از کمر درد دارد. پرسشنامه ی باور های ترس اجتنابی برای ارزیابی فاکتورهای سایکولوژیکال استفاده می شود. این پرسشنامه شامل 16 آیتم در دو بخش می باشد. بخش اول درد مرتبط با باورهای ترس اجتنابی مرتبط با کار افراد و بخش دوم مرتبط با فعالیت فیزیکی آن ها می باشد. به هر ایتم از 0 تا 6 نمره دهی می شود. مطابق شواهد ICC ورژن فارسی این پرسشنامه 0.80 می باشد (86)

6- دستگاه اولتراسونوگرافی برای محاسبه ی ژئومتری عضلات عرضی شکمی ، مایل داخلی شکمی ، مایل خارجی شکمی و مولتی فیدوس کمری

**روش انجام کار**:

افراد داوطلب پس از آگاهی از شرایط شرکت در مطالعه با تکمیل فرم رضایت آگاهانه ، آگاهی از نحوه ی انجام کار و دارا بودن معیار های ورود و خروج وارد مطالعه خواهند شد و مشخصات دموگرافیک شامل سن ، جنس ، قد ، وزن ، شاخص توده ی بدنی و تاریخچه ی بیماری فرد ، تاریخ مراجعه و داروهای مصرفی در فرم جمع آوری اطلاعات ثبت خواهد شد. پس از پر کردن پرسشنامه های مرتبط با درد ، ناتوانی، ترس از حرکت و فاجعه انگاری درد ، ضخامت عضلات شکمی و مولتی فیدوس و سطح مقطع عضله ی مولتی فیدوس با استفاده از دستگاه اولتراسونوگرافی مدل HS-2600 (ساخت شرکت هوندای ژاپن) محاسبه خواهد شد. تصاویر سونوگرافی توسط ترنسدیوسر 5/7 مگاهرتز خطی و اولتراسوند B-mode ثبت خواهد شد و رزولوشن و gain دستگاه طوری تنظیم می شود که تصاویری واضح را نشان دهد. برای محاسبه ی ضخامت عضلات عرضی شکمی ، مایل داخلی و خارجی ، فرد در وضعیت سوپاین با زانوهای خم قرار میگیرد و درمانگر در کنار او قرار می گیرد. پس از تعیین نقطه مياني خط anterior axillary lineدر ناحيه بين ASIS و آخرین دنده(28) آزمونگر پروب دستگاه را آغشته به ژل نموده و در نقطه ی تعيين شده بصورت عرضي قرار ميدهد . این مکان شفاف ترین تصویر را از عضلات عرضی شکمی ، مایل داخلی و خارجی به صورت همزمان نشان می دهد . تصاویر در حالت استراحت عضله و در حالت انقباض حين انجام مانور abdominal drawing in در انتهای بازدم ثبت مي گردد . برای ثبت تصاویر عضلات شکمی در حالت انقباض فرد سوپاین در پوزیشن hook-lying میخوابد سپس از وی خواسته می شود که قسمت تحتانی شکم را در انتهای بازدم به سمت ستون فقرات بکشد. دو ثبت در سمت راست و دو ثبت در سمت چپ انجام می شود و هر انقباض به مدت 5 تا 10 ثانیه حفظ می شود(75). برای محاسبه ی ضخامت و سطح مقطع عضله ی مولتی فیدوس فرد به حالت دمر می خوابد ، دست ها کنار بدن و سر در سوراخ بالای تخت در خط وسط قرار میگیرند و برای به حداقل رساندن لوردوز کمری یک بالشت زیر شکم فرد گذاشته می شود (88).سپس نقطه ی وسط پروب به صورت طولی در خط وسط روی زائده ی خاری مهره ی چهارم کمری گذاشته می شود( زائده خاری مهره ی چهارم به صورت ساژیتالی برجسته است و با لمس از زائده ی خاری مهره ی پنجم کمری به سمت بالا مشخص می شود. زائده ی خاری مهره ی پنجم یک برجستگی استخوانی کوچک و عمقی است که در مرکز فرورفتگی لومبوساکرال قرار دارد) تا یک اسکن از زائده خاری را ایجاد کند . سپس پروب حدود 2 سانتی متر به سمت خارج حرکت داده می شود و کمي آن را به صورت سفالو کادال و مدیولترال چرخانده بطوریكه زاویه کوچكي(حدود 10 درجه) با خط وسط بدن پيدا کند تا مفصل zygapophyseal L5-L4 مشخص شود و تصویر پاراساژیتال عضله ی مولتی فیدوس در حالت استراحت به دست آید(89). برای محاسبه ضخامت عضله مولتي فيدوس از فاسيای عضله در زیر بافت زیر پوستي تا سایه اکوژنيک لامينا محاسبه مي گردد و برای اندازه گيری سطح مقطع عضله مولتي فيدوس ترنسدیوسر بصورت عرضي روی زائده ی خاری مهره چهارم کمری گذاشته مي شود تا تصویر مولتی فیدوس هر دوسمت همزمان ثبت شود . در مواردی که این امر امکان پذیر نباشد ثبت از هر طرف به صورت جداگانه انجام می شود. در محاسبه سطح مقطع حاشيه های عضله مشخص مي شود بدین ترتيب که سایه اکوژنيک لامينای مهره چهارم کمری بعنوان عمقي ترین border عضله(88)، از فاسيای توراکولومبار بعنوان حاشيه بالایي،از سایه اکوستيک زائده ی خاری مهره به عنوان حاشيه داخلي استفاده می شود. همچنين برای مشخص نمودن حاشيه خارجي مهره از فاسيای جدا کننده این عضله و عضله لانجيسموس استفاده می شود. در صورتی که تشخیص لبه ی خارجی عضله ی مولتی فیدوس از لانجیسیموس دشوار باشد از فرد خواسته می شود که پای همان سمت را کمی بلند کند و سپس قبل از ثبت تصویر پایین بیاورد(88). محاسبه سطح مقطع عضله ی مولتی فیدوس توسط رسم دو خط انجام مي شود. ابتدا خطي درمحلي که بيشترین عمق قدامي-خلفي عضله را مشخص کرده رسم مي کنيم، سپس خطي از بيشترین عرض جانبي عضله بر خط اول عمود کرده و مساحت عضله بدست مي آید(88) و ضخامت عضلات توسط کشيدن خط بين کناره های داخلي فاسيای عضلات برحسب ميلي متر مشخص مي شود(79). تصاویر در پوزیشن خوابیده در سطح L4/L5 هر دو سمت در حالت استراحت و انقباض به دست می آید(16). در حالت انقباض به دست سمت مخالف فرد یک وزنه داده می شود. وزن وزنه ی مدنظر بر مبنای وزن بدن فرد در نظر گرفته می شود به طوری که اگر وزن فرد کمتر از 2/68 باشد مقدار وزنه 68/0 کیلوگرم ، بین 2/68 تا 9/90 مقدار وزنه 9/0 کیلوگرم و بیشتر از 9/90 مقدار وزنه 36/1 خواهد بود. از فرد خواسته می شود تا دستی که وزنه را گرفته است 5 سانتی متر از تخت بلند کند در حالیکه شانه در 120 درجه ابداکشن و آرنج در 90 درجه ی فلکشن قرار دارد . میزان لود وارد شده توسط وزنه به عضله ی مولتی فیدوس حدود 30 درصد حداکثر انقباض ارادی ایزومتریک می باشد. فرد هر انقباض را در انتهای بازدم نرمال 3 ثانیه نگه میدارد و در هر سمت بلند کردن دست 3 بار انجام خواهد شد(16). پس از ثبت تصاویر در حالت خوابیده ، در حالت نشسته و ایستاده مجددا تصاویر مد نظر ثبت خواهد شد. برای حالت نشسته فرد روی یک صندلی به ارتفاع 43 سانتی متر بدون دسته با پشتی می نشیند در حالیکه ستون فقرات در وضعیت نوترال می باشد ، دست ها روی شانه ی مقابل ریلکس قرار گرفته اند و پاها روی زمین هستند(31).سپس در دو حالت استراحت و انقباض(با انجام مانور ADIM برای عضلات شکمی و استفاده از وزنه برای عضله ی مولتی فیدوس) مشابه وضعیت خوابیده ثبت تصاویر برای عضلات شکمی و مولتی فیدوس انجام خواهد شد(90). برای محاسبه ی ژئومتری عضلات در حالت ایستاده ابتدا فرد می ایستد و دست ها را ریلکس کنار بدن قرار میدهد . برای اینکه فرد در حالت ایستادن معمول خود قرار بگیرد از وی خواسته می شود تا برای چند ثانیه روی یک نقطه چند گام بردارد و سپس در همان نقطه بایستد و به یک نقطه در مقابل خود مستقیما نگاه کند. ثبت تصاویر برای عضلات مطابق حالت خوابیده در حالت استراحت در انتهای بازدم انجام می شود. سپس برای منقبض کردن عضلات شکمی از مانور ADIM استفاده می شود و برای منقبض کردن مولتی فیدوس یک وزنه به دست مقابل فرد می دهند درحالیکه شانه ی فرد در 90 درجه فلکشن و آرنج در اکستنشن کامل است .فرد سه ثانیه وزنه را نگه میدارد و تصاویر برای حالت انقباض به دست می آید(16) . تغییرات ضخامت در وضعیت نشسته و ایستاده به ضخامت واقعی عضلات در وضعیت سوپاین نرمالیزه می شود .

سپس شرکت کنندگان به صورت تصادفی به دو گروه تمرین درمانی با تمرینات ثباتی با استفاده از فلکسی بار همراه با PNE و گروه کنترل( تمرین درمانی با تمرینات ثباتی با استفاده از فلکسی بار به تنهایی ) تقسیم خواهند شد. تعداد جلسات تمرینات 3جلسه در هفته به مدت 6 هفته (64) بوده است. در هر دو گروه ابتدای هر جلسه ی درمانی الکتروتراپی شامل 20 دقیقه TENS فرکانس پایین (91)( فرکانس1-4 هرتز، duration 100-400 میکروثانیه و آمپلی تیود در حد تحمل بیمار) ، hot pack به مدت 20 دقیقه و اولتراسوند مداوم با فرکانس 1 MHz و شدت 5/1 W/cm^2^ به مدت 5 دقیقه برای بیمار استفاده می شود. ابتدا و انتهای هر جلسه به مدت 5 دقیقه افراد گرم کردن و سرد کردن انجام می دهند. قبل از انجام تمرینات به هر دو گروه آموزش داده می شود که شکم را به سمت بالا و داخل بکشند تا یک تقعر در ناحیه ی شکمی ایجاد شود و سپس به مدت 10 ثانیه با حفظ وضعیت نوترال ستون فقرات مانور ADIM را انجام دهند. سپس افراد هر دو گروه تمرینات ثباتی را با استفاده از فلکسی بار انجام خواهند داد. به این منظور افراد در وضعیت ایستاده ، hook-lying ، چهار دست و پا و دمر مانور ADIM به مدت 10 ثانیه و نیز تمرینات supine bridge, curl-up four-point support)(65) راانجام می دهند. برای نگه داشتن فلکسی بار افراد از یک دست استفاده کرده و فلکسی بار را در جهت عمودی به صورت قدامی خلفی در حالیکه شانه در 90 درجه ی فلکشن قرار گرفته ، حرکت میدهند(92). هر حرکت 10 بار در سه ست تکرار می شود.بین هر ست افراد به مدت 3 دقیقه استراحت خواهند داشت(64).

پس از اتمام تمرینات ثباتی با استفاده از فلکسی بار ، در گروه آزمایش آموزش های PNE یک بار در هفته(68) به مدت 6 هفته ارائه خواهد شد. این آموزش ها به صورت فرد به فرد به مدت 30تا 60 دقیقه(69) توسط اسلاید های تهیه شده داده خواهد شد که به طور کلی شامل نوروفیزیولوژی درد ، مسیر های Nociception and nociceptive ، سیناپس ها، پتانسیل عمل ها، تسهیل ها و مهار های نخاعی، حساس شدگی محیطی و مرکزی، شکل پذیری^[28]^ سیستم عصبی ، فاکتور های فیزیولوژیکال و باور های مرتبط با درد می باشد.

# **تجزیه و تحلیل داده ها:**

آمار توصیفی شامل میانگین (Mean)، انحراف معیار (Standard Deviation)، فراوانی (Frequency) و درصد می باشد.

آمارهای استنباطی شامل آزمون تی مستقل(Independent sample t t-test) یا آزمون من ویتنی(Mann Whitney test)، آزمون تی زوجی (Paired sample T-test) یا معادل ناپارامتری آن آزمون ویلکاکسون (Wilcoxon Test)، آزمون اندازه گیری تکراری (Repeated Measures test) یا معادل ناپارامتری آن آزمون فریدمن (Freidman Test)، می باشد.

برای انجام تجزیه و تحلیل اطلاعات از نرم­افزار SPSS نسخه 26 استفاده می شود، همچنین سطح معناداری 05/0 در نظر گرفته شد.

**الف نوع مطالعه :**

**نوع مطالعه را مشخص فرمایید ودر قسمت نحوه انجام کار موارد مقابل بند مورد ا شاره راتوضیح دهید .**

| محل علامت | نوع مطالعه | مواردی که باید الزاماً در روش اجرای طرح توضیح داده شود |
| --- | --- | --- |
|  | بررسی بیماران (Case Series) | تعریف بیماری – جمعیت مورد مطالعه – محلهای مورد مطالعه |
|  | بررسی مقطعی (Cross Sectional) | جمعیت مورد مطالعه – نام متغیرها – روشهای نمونه گیری |
|  | مطالعه مورد / شاهد/Control) (Case | تعریف گروه مورد ( مثلاً بیماران ) و چگونگی انتخاب آنان – تعریف گروه کنترل و چگونگی انتخاب آنان – نسبت شاهد به مورد – نام متغیر مستقل اصلی که مورد بررسی قرار می گیرد . |
|  | مطالعه هم گروهی (Cohort) | به صورت اینده نگر (Prospective)یا گذشته نگر(Retrospective) تعریف جمعیت مورد مطالعه – تعریف دقیق مواجهه – تعریف دقیق Outcome– نحوه مقابله با Loss |
| * | مطالعه مداخله ای (interventional)و یا کارآزمایی بالینی(Clinical trial) | نوع مطالعه – نوع نمونه انسانی یا حیوانی – تعریف نحوه مداخله و میزان دقیق آن 0 طول مدت دوز مورد مصرف و ... ) وجود گروه کنترل – نحوه تقسیم در گروه های مختلف (Allocation)– نحوه کورکردن مطالعه – نحوه مقابله با خروج نمونه ها از مطالعه(Withdrawal) – تعریف دقیق پیامد (Outcome) |
|  | مطالعه علوم پایه (Experimental) | تعریف دقیق دارو یا لوازم – آیا مشابه خارجی دارد درصورت وجود کاتولوگ آن پیوست می باشد – موارد مصرف – نحوه تایید دستگاه یا دارو |
|  | راه اندازی یک روش یا سیستم علمی – اجرایی |  |
|  | بررسی تستها | تعریف دقیق انجام تست – تعریف دقیق تست Gold Standard– نحوه پذیرش بیماران و افراد سالم |
|  | بررسی روشها | مشخصات دقیق روش مورد نظر – مشخصات دقیق روش مرسوم (routine)– تعریف دقیق تفاوتها – نحوه پذیرش بیماران و افراد سالم |
|  | مطالعات کیفی | تعریف دقیق گروه های مورد نظر – نحوه اجرای جلسات و هدایت بحثها – معرفی گردانندگان جلسات و تخصص آنها – نحوه نتیجه گیری |
|  | مطالعات مدیریت سیستم بهداشتی |  |

3-فهرست منابع References :

الف – کليه منابع مورد استفاده در مقدمه و روش پژوهش را در این قسمت با ذکر مشخصات منبع معرفي فرمایيد . (پيوست نمودن دو مقاله از منابع اصلي مورد استفاده الزامي است (

1.	Khandhar RY, Paul J. Comparative Effect of Trunk Balance Exercise over Conventional Back Care Exercise in Patients with Chronic Mechanical Low Back Pain. Indian Journal of Public Health Research & Development. 2020;11(6).

2.	Bansal D AM, Ghai B, Pushpendra D. Prevalence and Impact of Low Back Pain in a Community-Based Population in Northern India. 2020;23.

3.	Freburger JK, Holmes GM, Agans RP, Jackman AM, Darter JD, Wallace AS, et al. The rising prevalence of chronic low back pain. Archives of internal medicine. 2009;169(3):251-8.

4.	Kaur S, Koley S. Effectiveness of trunk balance exercises and wii fit TM balance exercises in managing disability and pain in patients with chronic low back pain. Int J Health Sci. 2019;6:5.

5.	Ghafouri M TA, Nakhostin-Ansari A, Sepanlou SG, Dalvand S, Moradpour F, Bavarsad AH, Boogar SS, Dehghan M, Ostadrahimi A, Aghazadeh-Attari J. revalence and predictors of low back pain among the Iranian population: Results from the Persian cohort study. 2022;10.

6.	Karpuz Ş BU, Kutlutürk S, ÇOLAK TK. Effectiveness of Balance Exercises on Postural Control and Quality of Life in Patients with Lumbar Discopathy. 2021;11.

7.	Hurwitz EL, Randhawa K, Yu H, Côté P, Haldeman S. The Global Spine Care Initiative: a summary of the global burden of low back and neck pain studies. European Spine Journal. 2018;27(6):796-801.

8.	Dieleman JL, Baral R, Birger M, Bui AL, Bulchis A, Chapin A, et al. spending on personal health care and public health, . 2016;316.

9.	Frieder Krausea DN, Winfried Banzer. Medical exercise and physiotherapy modes and frequency as predictors for a recurrence of chronic non-specific low back pain. 2021;34(665).

10.	Meucci RD FA, Faria NM. Prevalence of chronic low back pain:systematic review. . 2015;49(1).

11.	backache KSNcf. biopsychosocial pain syndrome. . 2008;17(54).

12.	Bardin LD KP, Maher CG. Diagnostic triage for low back pain: a practical approach for primary care. . 2017;206.

13.	Savigny P WP, Underwood M. Early management of persistent nonspecific low back pain. summary of NICE guidance Bmj. 2009;4.

14.	Qaseem A WT, McLean RM, Forciea MA. Clinical Guidelines Committee of the American College of Physicians*. Noninvasive treatments for acute, subacute, and chronic low back pain: a clinical practice guideline from the American College of Physicians. . 2017;4.

15.	Rahmani N MBM, Salavati M, Vameghi R, Abdollahi I. Comparative Study of Abdominal Muscle Thickness onUltrasonography inHealthy Adolescents and Patients With Low Back Pain. 2018;37.

16.	Schryver A RH, Rizk A, Frenette S, Boily M, Fortin M. Ultrasonography of Lumbar Multifidus Muscle in University American Football Players. 2020;52.

17.	Freeman MD WM, Woodham AW. The role of the lumbar multifidus in chronic low back pain: a review. 2010;2.

18.	Arab AM SS, Rasouli O, Amiri M, Ehsani F. Automatic activity of deep and superfcial abdominal muscles during stable and unstable sitting positions in individuals with chronic low back pain. 22(3):627–631. . 2018;22(3).

19.	Scholtes SA, Gombatto SP, Van Dillen LR. Differences in lumbopelvic motion between people with and people without low back pain during two lower limb movement tests. Clinical Biomechanics. 2009;24(1):7-12.

20.	Lunde LK KM, Knardahl S, Veiersted KB. Associations of objectively measured sitting and standing with low-back pain intensity: a 6-month follow-up of construction and healthcare workers. 2017;1.

21.	Murray E BE, Twycross-Lewis R et al. The relationship between hip rotation range of movement and low back pain prevalence in amateur golfers: an observational study. 2009;10(4).

22.	Bivona G, Koch C, Garcia-Augundez A, Göbel S, Hänsel F. A case control study to investigate differences in motor control between individuals with and without non-specific low back pain during standing. 2021;15.

23.	Le Ge CW, Haohan Zhou. Effects of low back pain on balance performance in elderly people: a systematic review and meta-analysis. 2021;18.

24.	Astfalck RG, O’Sullivan, P.B., Smith, A.J., Straker, L.M., Burnett, A.F. Lumbar spine repositioning sense in adolescents with and without non-specific chronic low back pain - An analysis based on sub-classification and spinal regions. . 18. 2013;18(5).

25.	Childs JD, Piva SR, Erhard RE, Hicks G. Side-to-side weight-bearing asymmetry in subjects with low back pain. Manual Therapy. 2003;8(3):166-9.

26.	Osuka S KY, Yamanaka M, Miura T, Saito Y, Ueno R, Ishida T, Samukawa M, Tohyama H. The onset of deep abdominal muscles activity during tasks with different trunk rotational torques in subjects with non-specific chronic low back pain. . 2019;24.

27.	Teyhen DS, Williamson JN, Carlson NH, Suttles ST, O'Laughlin SJ, Whittaker JL, et al. Ultrasound characteristics of the deep abdominal muscles during the active straight leg raise test. Archives of physical medicine and rehabilitation. 2009;90(5):761-7.

28.	Ehsani F AA, Salavati M, Jaberzadeh S, Hajihasani A. Ultrasound measurement of abdominal muscle thickness with and without transducer fixation during standing postural tasks in participants with and without chronic low back pain. 2016;8.

29.	Zachovajeviene B SL, Zachovajevas P, Venclovas Z, Milonas D. Efect of diaphragm and abdominal muscle training on pelvic foor strength and endurance: results of a prospective randomized trial. 2019;9.

30.	Fortin M RA, Frenette S, Boily M, Rivaz H. Ultrasonography of multifidus muscle morphology and function in ice hockey players with and without low back pain. 2019;1.

31.	Rasouli O SS, Arab AM, ShahAli S, Sarafraz H. The effect of respiratory phase on abdominal muscle activity during stable and unstable sitting positions in individuals with and without chronic low back pain. 2020;43.

32.	Huang Q ZY, Li D, Yang D, Huo M, Maruyama H. The evaluation of chronic low back pain by determining the ratio of the lumbar multifidus muscle cross-sectional areas of the unaffected and affected sides. . 2014;26.

33.	Prentice CL MS, Massy-Westropp N, Maranna S. The reliability of rehabilitative ultrasound to measure lateral abdominal muscle thickness: A systematic review and meta-analysis. . . 2021;53.

34.	Mangum LC SM, Saliba SA, Hart JM. Reliability of Ultrasound Imaging Measures of Transverse Abdominis and Lumbar Multifidus in Various Positions. 2015;8.

35.	Ung H BJ, Johnson KA, Younger J, Hush J, Mackey S. Multivariate Classification of Structural MRI Data Detects Chronic Low Back Pain. 2014;24.

36.	Chou R QA, Owens DK, Shekelle P. Diagnostic imaging for low back pain: advice for high-value health care from the American College of Physicians. 2011;154.

37.	Yarnitsky D A-NL, Bouhassira D, Edwards RR, Fillingim RB, Granot M, Hansson P, Lautenbacher S, Marchand S, Wilder-Smith O. Recommendations on terminology and practice of psychophysical DNIC testing. 2010;14.

38.	Lewis GN RD, McNair PJ. Conditioned pain modulation in populations with chronic pain:. 2012;13.

39.	Trinderup JS FA, Juhl CB, Petersen T. Fear avoidance beliefs as a predictor for long-term sick leave, disability and pain in patients with chronic low back pain. . 2018;19.

40.	Suh JH KH, Jung GP, Ko JY, Ryu JS. The effect of lumbar stabilization and walking exercises on chronic low back pain: a randomized controlled trial. . 2019;98.

41.	Henchoz Y, So AK-L. Exercise and nonspecific low back pain: a literature review. Joint Bone Spine. 2008;75(5):533-9.

42.	Laird RA KP, Keating JL. Modifying patterns of movement in people with low back pain -does it help? A systematic review. 2012;13.

43.	Richardson C, Jull G, Hides J, Hodges P. Therapeutic exercise for spinal segmental stabilization in low back pain: Churchill Livingstone London; 1999.

44.	Leonard JH PA, Sitilertpisan P, Pirunsan U, Uthaikhup S. Changes in transversus abdominis muscle thickness after lumbo-pelvic core stabilization training among hronic low back pain individuals. 2015;166.

45.	Urquhart DM, Hodges PW, Allen TJ, Story IH. Abdominal muscle recruitment during a range of voluntary exercises. Manual therapy. 2005;10(2):144-53.

46.	Souza GM, Baker LL, Powers CM. Electromyographic activity of selected trunk muscles during dynamic spine stabilization exercises. Archives of physical medicine and rehabilitation. 2001;82(11):1551-7.

47.	Teyhen DS, Miltenberger CE, Deiters HM, Del Toro YM, Pulliam JN, Childs JD, et al. The use of ultrasound imaging of the abdominal drawing-in maneuver in subjects with low back pain. Journal of Orthopaedic & Sports Physical Therapy. 2005;35(6):346-55.

48.	Hodges PW, Richardson CA. Altered trunk muscle recruitment in people with low back pain with upper limb movement at different speeds. Archives of physical medicine and rehabilitation. 1999;80(9):1005-12.

49.	Mileva KN, Kadr M, Amin N, Bowtell JL. Acute effects of Flexi-bar vs. Sham-bar exercise on muscle electromyography activity and performance. The Journal of Strength & Conditioning Research. 2010;24(3):737-48.

50.	Cohen LG, Starr A. Vibration and muscle contraction affect somatosensory evoked potentials. Neurology. 1985;35(5):691-.

51.	Kim J-H, So K-H, Bae Y-R, Lee B-H. A comparison of flexi-bar and general lumbar stabilizing exercise effects on muscle activity and fatigue. Journal of physical therapy science. 2014;26(2):229-33.

52.	Amiri Doomari F, Roostayi MM. The effect of bar flexi-bar in patients with low back pain. The Scientific Journal of Rehabilitation Medicine. 2021.

53.	Gifford L. Pain, the tissues and the nervous system: a conceptual model. Physiotherapy. 1998;84(1):27-36.

54.	Nijs J, Meeus M, Cagnie B, Roussel NA, Dolphens M, Van Oosterwijck J, et al. A modern neuroscience approach to chronic spinal pain: combining pain neuroscience education with cognition-targeted motor control training. Physical therapy. 2014;94(5):730-8.

55.	Louw A, Diener I, Puentedura EJ. The short term effects of preoperative neuroscience education for lumbar radiculopathy: A case series. International journal of spine surgery. 2015;9.

56.	Louw A, Zimney K, Puentedura EJ, Diener I. The efficacy of pain neuroscience education on musculoskeletal pain: a systematic review of the literature. Physiotherapy theory and practice. 2016;32(5):332-55.

57.	Lee K-SKJAJ-OKM-YLB-H. Effects of Pain Neuroscience Education Combined with Lumbar Stabilization Exercise on Strength and Pain in Patients with Chronic Low Back Pain: Randomized Controlled Trial. 2020.

58.	Abbasi S, Hadian Rasanani, M.R., Olyaei, G.R., Nastaran Ghotbi, Rasouli,. Reliability of ultrasound measurement of the lateral abdominal and lumbar multifidus muscles in individuals with chronic low back pain: a cross-sectional test-retest study, . 2020.

59.	Cañeiro J, Ng L, Burnett A, Campbell A, O'Sullivan P. Cognitive functional therapy for the management of low back pain in an adolescent male rower: a case report. journal of orthopaedic & sports physical therapy. 2013;43(8):542-54.

60.	Noormohammadpour P KM, Mansournia MA, Akbari-Fakhrabadi M, Kordi R. The role of a multi-step core stability exercise program in the treatment of nurses with chronic low back pain: a single-blinded randomized controlled trial. . 2018;12.

61.	Ko KJ HG, Yook YS, Kang SJ. . Effects of 12-week lumbar stabilization exercise and sling exercise on lumbosacral region angle, lumbar muscle strength, and pain scale of patients with chronic low back pain. 2018;30.

62.	Bae CR JY, Yoon BC, Kim NH, Park KW, Lee SH. Effects of assisted sit-up exercise compared to core stabilization exercise on patients with non-specific low back pain: a randomized controlled trial. . 2018;31.

63.	Gheitasi M KA, Daneshjoo A. The effect of combined core stability and sensory-motor exercises on Pain, Performance and movement fear in retired male athletes with non-specific chronic low back pain. . 2020;11.

64.	Chung SH YY, Lee HJ, Sim SH. Effects of stabilization exercise using flexi-bar on functional disability and transverse abdominis thickness in patients with chronic low back pain. 2018;30.

65.	SUN Y-m, PEI Q, HUANG Q-c, LI L, LIU K-m. Effects of Flexi-bar on Nonspecific Low Back Pain 2021.

66.	Adriaan Louw KZ, PT, DPT. The efficacy of pain neuroscience education on musculoskeletal pain: A systematic review of the literature. 2016.

67.	Malfliet A KJ, Coppieters I, De Pauw R, Meeus M, Roussel N, Cagnie B, Danneels L, Nijs J. Effect of Pain Neuroscience Education Combined With Cognition-Targeted Motor Control Training on Chronic Spinal Pain. 2018;75.

68.	Saracoglu I AM, Afsar E, Gokpinar HH. The effectiveness of pain neuroscience education combined with manual therapy and home exercise for chronic low back pain: A single-blind randomized controlled trial. 2020.

69.	Kim KS AJ, Kim JO, Lee MY, Lee BH. . Effects of Pain Neuroscience Education Combined with Lumbar Stabilization Exercise on Strength and Pain in Patients with Chronic Low Back Pain: Randomized Controlled Trial. 2022;12.

70.	Rasool Bagheri P, Rozita Hedayati, PhD, Fatemeh Ehsani, PhD, Nasim Hemati-Boruojeni, MSc. Cognitive Behavioral Therapy With Stabilization Exercises Affects Transverse Abdominis Muscle Thickness in Patients With Chronic Low Back Pain: A Double-Blinded Randomized Trial Study. 2020.

71.	Wesselink E dRE, Pevenage P, van der Kaay N, Pool J. Fear-avoidance beliefs are associated with a high fat content in the erector spinae: a 1.5 tesla magnetic resonance imaging study. . 2019;27.

72.	Akbari A KS, Abdi G. The effect of motor control exercise versus general exercise on lumbar local stabilizing muscle thickness. 2008;21.

73.	Richardson C JG, Hides J, et al.:. Therapeutic exercise for spinal segmental stabilization in low back pain: Scientific basis and clinical approach. . 1999.

74.	Amiri Doumari F, Rustic, Mohammad Mohsen. . Effect of loadflexi in patients with low back pain (review article). 2021.

75.	Bagheri R, Hedayati R, Ehsani F, Hemati-Boruojeni N, Abri A, Delkhosh CT. Cognitive behavioral therapy with stabilization exercises affects transverse abdominis muscle thickness in patients with chronic low back pain: A double-blinded randomized trial study. Journal of Manipulative and Physiological Therapeutics. 2020;43(5):418-28.

76.	Roland M, Morris R. A study of the natural history of back pain: Part 1: Development of a reliable and sensitive measure of disability in low-back pain. spine. 1983.

77.	Gatti R FS, Tettamanti A, Barbero M, Balestri A, Calori G. Efficacy of trunk balance exercises for individuals with chronic low back pain. 2011;41.

78.	Bogaerts A VS, Delecluse C, Claessens AL, Boonen S. Effects of whole body vibration training on postural control in older individuals: A 1 year randomized controlled trial. 2007;26.

79.	Vasseljen O FA. Abdominal muscle contraction thickness and function after specific and general exercises: A randomized controlled trial in chronic low back pain patients. 2010;15.

80.	Pozo-Cruz BD HMM, Adsuar JC, Parraca JA, Muro I, Gusi N. Effects of whole body vibration therapy on main outcome measures for chronic non-specific low back pain: a single-blind randomized controlled trial. 2011;43(8).

81.	Bervis S, Kahrizi S, Parnianpour M, Amirmoezzi Y, Shokouhyan S, Motealleh A. Amplitude of Electromyographic Activity of Trunk and Lower Extremity Muscles during Oscillatory Forces of Flexi-Bar on Stable and Unstable Surfaces in People with Nonspecific Low Back Pain. Journal of Biomedical Physics and Engineering. 2020.

82.	Shafshak TSM, Elnemr RM. The Visual Analogue Scale Versus Numerical Rating Scale in Measuring Pain Severity and Predicting Disability in Low Back Pain. 2021;27(7).

83.	Mousavi SJ, Parnianpour M, Mehdian H, Montazeri A, Mobini B. The Oswestry Disability Index, the Roland-Morris Disability Questionnaire, and the Quebec Back Pain Disability Scale: Translation and Validation Studies of the Iranian Versions. Spine. 2006;31(14):E454-E9.

84.	Jafari H EI, Salavati M, Kamali M, Fata L. Psychometric properties of Iranian version of Tampa Scale for Kinesiophobia in low back pain patients. 2010;11.

85.	aeissadat SA SS, Montazeri A. Validation of the pain catastrophizing scale (PCS) in Iran. 2013;3.

86.	Rostami M NN, Mansournia MA, Sharafi E, Babaki AE, Kordi R. Validation of the Persian version of the fear avoidance belief questionnaire in patients with low back pain. 2014;27.

87.	Hosseinifar M, Akbari M, Behtash H, Amiri M, Sarrafzadeh J. The effects of stabilization and McKenzie exercises on transverse abdominis and multifidus muscle thickness, pain, and disability: a randomized controlled trial in nonspecific chronic low back pain. Journal of physical therapy science. 2013;25(12):1541-5.

88.	Stokes M RG, Newham D. Ultrasound imaging of lumbar multifidus muscle: normal reference ranges for measurements and practical guidance on the technique. . 2005;10.

89.	Larivière C, Gagnon DH, Henry SM, Preuss R, Dumas JP. The Effects of an 8‐Week Stabilization Exercise Program on Lumbar Multifidus Muscle Thickness and Activation as Measured With Ultrasound Imaging in Patients With Low Back Pain: An Exploratory Study. PM&R. 2018;10(5):483-93.

90.	Arab AM, Shanbehzadeh S, Rasouli O, Amiri M, Ehsani F. Automatic activity of deep and superficial abdominal muscles during stable and unstable sitting positions in individuals with chronic low back pain. Journal of bodywork and movement therapies. 2018;22(3):627-31.

91.	Buchmuller A NM, Milletre‐Bernardin M, Pouplin S, Presles E, Lantéri‐Minet M, et al. Value of TENS for relief of chronic low back pain with or without radicular pain. . 2012;16(5).

92.	Herasi M, Kahrizi S, Hoviattalab M. Evaluation of Trunk Muscle Activation during the Two Directions of Flexi-Bar Exercise in People with and without Low Back Pain. Modern Rehabilitation. 2022;16(4).

**پیش بینی زمان لازم برای اجرای کامل طرح:**

جدول زماني مراحل اجراء و پيشرفت كار :

| رديف | شرح هريك از فعاليتهاي اجرايي طرح به تفكيك | طول مدت | زمان اجرا | | | | | | | | | | | | | | | | | |
| --- | --- | --- | --- | --- | --- | --- | --- | --- | --- | --- | --- | --- | --- | --- | --- | --- | --- | --- | --- | --- |
|  |  |  | **1** | **2** | **3** | **4** | **5** | **6** | **7** | **8** | **9** | **10** | **11** | **12** | **13** | **14** | **15** | **16** | **17** | **18** |
|  | تهيه پروپوزال و تصویب آن |  |  |  |  |  |  |  |  |  |  |  |  |  |  |  |  |  |  |  |
|  | گرد آوری داده ها |  |  |  |  |  |  |  |  |  |  |  |  |  |  |  |  |  |  |  |
|  | آناليز آماری و تجزیه و تحليل نتایج |  |  |  |  |  |  |  |  |  |  |  |  |  |  |  |  |  |  |  |
|  | تهيه گزارش نهایي |  |  |  |  |  |  |  |  |  |  |  |  |  |  |  |  |  |  |  |

**لطفاً زمان هاي ارائه گزارش پیشرفت کار به معاونت پژوهشي را حداقل در 3 نوبت ذکر فرمایید .**

-بعد از جمع آوری نیمی از داده ها.

-بعد از انالیز آماری

-بعد از تهیه ی گزارش نهایی

3 2 - مشكلات اجرایي در انجام طرح و روش حل مشكلات :

-عدم همکاری بیماران به دلیل طولانی بودن روند درمانی که این مورد با توضیح اثرات مثبت درمانی برای بهبود علائم بیمار با تکمیل پروسه ی درمانی حل خواهد شد

-اثر گذاری فاکتورهای اجتماعی ، اقتصادی و مشکلات فردی بر نتایج مرتبط با PNE با طولانی شدن زمان درمان که این مورد با بیان توضیحاتی برای بیمار درباره ی حساسیت نتایج و کنترل شرایط روانی و محیطی تا حد امکان حل خواهد شد

-عدم دستيابي به تعداد بيمار مناسب در زمان در نظر گرفته شده، که با مراجعه به درمانگاه های مختلف و پزشكان متخصص این مشكل رفع خواهد شد.

-ایجاد مشكلات پزشكي در برخي از بيماران در طي پروسه تحقيق که با خروج بيمار از مطالعه حل خواهد شد.

24-ملاحظات اخلاقي مربوط به طرح پژوهشي :

آیا تكميل و اخذ فرم رضایت از افراد مورد پژوهش ضروری است ؟ همه ی بيماران با رضایت آگاهانه وارد مطالعه خواهند شد و به آن ها اطمينان داده مي شود که اطلاعات آنها محرمانه باقي خواهد ماند. شرکت کنندگان در این طرح حق انصراف در هر مرحله از تحقيق را دارا بوده و در صورت بروز هر گونه مشكل برای فرد̧ محققين مستول پيگيری و رفع نقصان پيش آمده خواهند بود.

در صورت مثبت بودن پاسخ سوال فوق لطفاً نمونه فرم رضایت نامه اخلاقي ضميمه گردد.

2-سایر موارد ملاحظات اخلاقي در اجرای طرح را ذکر فرمایيد

چه موسساتي از نتایج این پژوه بهره خواهند برد: کليه مراکز توانبخشي

اینجانب دکتر سارا ابوالاحراری ضمن تأیید صحت مطالب مندرج در پرسشنامه و مطالعه و آگاهي کامل از متن منشور مالكیت معنوي دانشگاه علوم پزشكي شیراز، خانم زهرا دهقانی را به عنوان رابط مالي جهت هزینه کرد اعتبارات طرح و یا مكاتبات با معاونت پژوهشي به عنوان نماینده معرفي مي نمایم.

محل امضای مجری اول طرح	محل امضای معاون پژوهشي

نظریه مشاور طراحي طرح پژوهشي :

**{تفاهم نامه پروژه هاي تحقيقاتي چند مركزي داخلي**

**با عنايت به ارج نهادن به انجام پروژه هاي تحقيقاتي چند مركزي و اهميت ويژه اي كه اينگونه پروژه ها در ارتقاء پژوهش دارند اين تفاهم نامه بين آقای/خانم................... از دانشگاه علوم پزشکی شیراز و آقای/خانم.................. از دانشگاه/ مرکز تحقیقاتی ................................ در خصوص انجام پروژه تحقيقاتي با مشخصات ذيل به امضاء مي رسد:**

**مشخصات عمومي**

**عنوان پروژه: .................................**

**مصوب صورتجلسه شوراي پژوهش مورخ ..............**

**مجري يا مجريان: .............................**

**محل اجراء: ............................**

**مدت زمان اجراء: ..............**

**مبلغ كل اعتبار: .................**

**موارد مورد تفاهم**

**همكاري هاي علمي: ..................................**

**سهم مشاركت مالي طرفين: ........................................**

**همكاري در استفاده از تجهيزات و لوازم:**

**حقوق و تعهدات**

**شرايط همكاري: ...........................................**

**شرايط فسخ:**

دكتر ................

معاون پژوهشي دانشگاه علوم پزشكي شيراز

**آقای/خانم** (مجری هیئت علمی دانشگاه همکار)............

............................

**آقای/خانم** (مجری هیئت علمی دانشگاه علوم پزشکی شیراز).................

........................

دكتر ................

معاون پژوهشي دانشگاه (همکار)

(این قرارداد در چهار نسخه تنظیم می گردد.)

1. Postural control
2. Transversus abdominis
3. Internal oblique
4. External oblique
5. orientation
6. Thoracolumbar
7. Reliability
8. central sensitization
9. diffuse noxious inhibitory controls
10. noxious
11. conditioned pain modulation (CPM)
12. Pressure pain thresholds
13. Pain catastrophizing
14. Cognitive behavioral therapy
15. nociceptive pain
16. processing mechanisms
17. Healthcare utilization
18. Plasticity
19. Cognition targeted
20. Numeric rating scale
21. neutral
23. intramuscular adipose tissue
24. individual specific motor behaviors
25. Avoidance behavior
26. fatty infiltration
27. Intra class correlation
28. Plasticity
