# Supplementary material for: The effect of integrating pain neuroscience education with stabilization exercises using a flexi-bar on functional disability, pain, and geometry of abdominal and multifidus muscles in patients with chronic non-specific low back pain
Source: PLoS One. 2026 Aug 27;21(8):e0355692. doi: 10.1371/journal.pone.0355692 (PMC13521579; doi:10.1371/journal.pone.0355692)
Supplement: Supplementary file 4 [file pone.0355692.s005.docx]

| **Muscle/condition** | **time** | **SE+PNE (n=24)**  **(Mean ±SD)** | **SE**  **(n=24)**  **(Mean ±SD)** | **Between-group difference[95%CI]** | **Effect size (Cohen’s *d*)** | **P value** |
| --- | --- | --- | --- | --- | --- | --- |
| **TA.RT.SUP.REST** | **Baseline** | 3.69±0.63 | 3.57±0.66 | 0.12[-0.25,0.50] | 0.19 | 0.513 |
|  | **Post-test** | 4.41±0.66 | 3.91±0.70 | 0.50[0.10,0.89] | 0.73 | 0.015* |
| **TA.LT.SUP.REST** | **Baseline** | 3.75±0.66 | 3.63±0.75 | 0.11[-0.29,0.53] | 0.16 | 0.568 |
|  | **Post-test** | 4.58±0.73 | 4.08±0.75 | 0.50[0.06,0.93] | 0.67 | 0.023* |
| **TA.RT.SUP.CONT** | **Baseline** | 4.48±0.68 | 4.29±0.82 | 0.20[-0.25,0.64] | 0.26 | 0.377 |
|  | **Post-test** | 5.31±0.83 | 4.74±0.86 | 0.57[0.07,1.06] | 0.67 | 0.026* |
| **TA.LT.SUP.CONT** | **Baseline** | 4.84±1.04 | 4.52±0.87 | 0.32[-0.23,0.88] | 0.33 | 0.247 |
|  | **Post-test** | 5.74±1.17 | 5.02±1.04 | 0.72[0.07,1.36] | 0.65 | 0.030* |
| **TA.RT.SIT.REST** | **Baseline** | 1.36±0.26 | 1.34±0.29 | 0.02[-0.14,0.18] | 0.07 | 0.808 |
|  | **Post-test** | 1.48±0.24 | 1.39±0.25 | 0.09[-0.07,0.25] | 0.36 | 0.263 |
| **TA.LT.SIT.REST** | **Baseline** | 1.08±0.30 | 1.07±0.27 | 0.01[-0.15,0.17] | 0.05 | 0.863 |
|  | **Post-test** | 1.29±0.24 | 1.21±0.24 | 0.08[-0.05.22] | 0.35 | 0.217 |
| **TA.RT. SIT.CONT** | **Baseline** | 1.69±0.23 | 1.63±0.21 | 0.06[-0.06,0.17] | 0.26 | 0.335 |
|  | **Post-test** | 1.84±0.25 | 1.77±0.20 | 0.07[-0.04,0.18] | 0.31 | 0.201 |
| **TA.LT. SIT.CONT** | **Baseline** | 1.48±0.33 | 1.36±0.27 | 0.12[-0.02,0.26] | 0.40 | 0.080 |
|  | **Post-test** | 1.66±0.35 | 1.55±0.29 | 0.11[-0.04,0.27] | 0.34 | 0.161 |
| **TA.RT.STND.REST** | **Baseline** | 1.37±0.24 | 1.37±0.22 | 0.00[-0.12,0.13] | 0.02 | 0.949 |
|  | **Post-test** | 1.51±0.22 | 1.46±0.26 | 0.05[-0.07,0.17] | 0.20 | 0.412 |
| **TA.LT.STND.REST** | **Baseline** | 1.10±0.28 | 1.12±0.23 | -0.02[-0.18,0.15] | 0.06 | 0.846 |
|  | **Post-test** | 1.27±0.26 | 1.25±0.27 | 0.02[-0.16,0.21] | 0.09 | 0.798 |
| **TA.RT.STND.CONT** | **Baseline** | 1.69±0.18 | 1.68±0.23 | 0.01[-0.10,0.13] | 0.06 | 0.819 |
|  | **Post-test** | 1.87±0.20 | 1.81±0.25 | 0.05[-0.07,0.18] | 0.23 | 0.390 |
| **TA.LT,STND.CONT** | **Baseline** | 1.47±0.31 | 1.42±0.24 | 0.05[-0.10,0.20] | 0.19 | 0.474 |
|  | **Post-test** | 1.66±0.33 | 1.56±0.28 | 0.10[-0.05,0.24] | 0.31 | 0.182 |
| **IO.RT.SUP.REST** | **Baseline** | 6.08±1.00 | 6.05±1.01 | 0.03[-0.55,0.62] | 0.03 | 0.913 |
|  | **Post-test** | 6.43±0.95 | 6.39±1.01 | 0.04[-0.52,0.62] | 0.05 | 0.866 |
| **IO.LT.SUP.REST** | **Baseline** | 5.74±1.46 | 5.72±0.94 | 0.01[-0.70,0.73] | 0.01 | 0.964 |
|  | **Post-test** | 6.31±1.47 | 6.05±1.01 | 0.25[-0.50,1.00] | 0.20 | 0.503 |
| **IO.RT.SUP.CONT** | **Baseline** | 6.71±0.99 | 6.70±0.97 | 0.01[-0.56,0.58] | 0.01 | 0.976 |
|  | **Post-test** | 7.11±1.03 | 7.01±0.98 | 0.10[-0.49,0.68] | 0.09 | 0.743 |
| **IO.LT.SUP.CONT** | **Baseline** | 6.56±1.45 | 6.50±1.11 | 0.05[-0.70,0.81] | 0.04 | 0.889 |
|  | **Post-test** | 6.70±1.48 | 6.93±1.25 | -0.23[-1.03,0.56] | -0.17 | 0.557 |
| **IO. RT.SIT.REST** | **Baseline** | 0.97±0.18 | 0.98±0.13 | 0.00[-0.10,0.09] | -0.03 | 0.916 |
|  | **Post-test** | 0.99±0.15 | 0.99±0.14 | 0.00[-0.09,0.08] | -0.01 | 0.960 |
| **IO. LT.SIT.REST** | **Baseline** | 1.05±0.24 | 1.04±0.28 | 0.01[-0.14,0.16] | 0.03 | 0.910 |
|  | **Post-test** | 1.06±0.22 | 1.05±0.29 | 0.00[-0.15,0.15] | 0.00 | 0.988 |
| **IO.RT.SIT.CONT** | **Baseline** | 0.91±0.15 | 0.91±0.15 | 0.00[-0.08,0.09] | 0.01 | 0.961 |
|  | **Post-test** | 093±0.12 | 0.92±0.14 | 0.01[-0.06,0.09] | 0.10 | 0.738 |
| **IO. LT.SIT.CONT** | **Baseline** | 1.05±0.22 | 1.06±0.30 | -0.01[-0.16,0.14] | -0.03 | 0.925 |
|  | **Post-test** | 1.08±0.23 | 1.06±0.28 | 0.02[-0.13,0.17] | 0.09 | 0.747 |
| **IO.RT. STND.REST** | **Baseline** | 1.11±0.18 | 1.10±0.16 | 0.01[-0.09,0.11] | 0.04 | 0.890 |
|  | **Post-test** | 1.13±0.14 | 1.12±0.15 | 0.01[-0.07,0.09] | 0.07 | 0.810 |
| **IO.LT. STND.REST** | **Baseline** | 1.08±0.25 | 1.08±0.31 | 0.00[-0.16,0.16] | 0.00 | 0.988 |
|  | **Post-test** | 1.09±0.22 | 1.09±0.32 | 0.00[-0.16,0.16] | -0.01 | 0.964 |
| **IO.RT.STND.CONT** | **Baseline** | 1.02±0.16 | 1.02±0.16 | 0.00[-0.09,0.09] | 0.02 | 0.950 |
|  | **Post-test** | 1.03±0.15 | 1.03±0.17 | 0.00[-0.10,0.09] | -0.01 | 0.965 |
| **IO.LT. STND.CONT** | **Baseline** | 1.05±0.22 | 1.07±0.30 | -0.02[-0.17,0.13] | -0.07 | 0.812 |
|  | **Post-test** | 1.11±0.22 | 1.06±0.28 | 0.04[-0.10,0.19] | 0.18 | 0.540 |
| **EO.RT.SUP.REST** | **Baseline** | 5.35±1.12 | 5.11±0.91 | 0.24[-0.40,0.87] | 0.23 | 0.465 |
|  | **Post-test** | 5.92±1.16 | 5.69±1.03 | 0.22[-0.36,0.81] | 0.20 | 0.450 |
| **EO.LT.SUP.REST** | **Baseline** | 4.85±0.95 | 4.51±0.78 | 0.34[-0.20,0.88] | 0.39 | 0.220 |
|  | **Post-test** | 5.55±1.05 | 5.09±0.92 | 0.46[-0.13,1.04] | 0.46 | 0.126 |
| **EO.RT.SUP.CONT** | **Baseline** | 4.94±0.85 | 4.92±0.92 | 0.02[-0.54,0.59] | 0.03 | 0.930 |
|  | **Post-test** | 5.42±0.90 | 5.28±0.98 | 0.13[-0.43,0.70] | 0.14 | 0.644 |
| **EO.LT.SUP.CONT** | **Baseline** | 4.35±0.86 | 4.26±0.83 | 0.09[-0.41,0.58] | 0.10 | 0.730 |
|  | **Post-test** | 4.78±0.95 | 4.57±0.90 | 0.21[-0.26,0.67] | 0.22 | 0.376 |
| **EO. RT.SIT.REST** | **Baseline** | 4.87±0.77 | 4.77±0.68 | 0.10[-0.34V0.54] | 0.13 | 0.665 |
|  | **Post-test** | 5.14±0.78 | 5.00±0.73 | 0.14[-0.31,0.59] | 0.18 | 0.545 |
| **EO. LT.SIT.REST** | **Baseline** | 4.40±0.82 | 4.40±0.63 | 0.00[-0.41,0.41] | 0.01 | 0.984 |
|  | **Post-test** | 4.90±0.84 | 4.73±0.69 | 0.17[-0.28,0.62] | 0.22 | 0.458 |
| **EO.RT.SIT.CONT** | **Baseline** | 4.68±0.81 | 4.60±0.67 | 0.07[-0.38,0.53] | 0.10 | 0.743 |
|  | **Post-test** | 4.93±0.87 | 4.79±0.71 | 0.14[-0.33,0.61] | 0.18 | 0.548 |
| **EO. LT.SIT.CONT** | **Baseline** | 4.34±0.84 | 4.36±0.79 | -0.01[-0.46,0.43] | 0.02 | 0.947 |
|  | **Post-test** | 4.52±0.86 | 4.49±0.74 | 0.03[-0.41,0.47] | 0.04 | 0.889 |
| **EO.RT.STND.REST** | **Baseline** | 4.82±0.76 | 4.85±0.83 | -0.03[-0.48,0.42] | 0.03 | 0.905 |
|  | **Post-test** | 5.13±0.76 | 5.12±0.80 | 0.02[-0.40,0.43] | 0.02 | 0.941 |
| **EO.LT. STND.REST** | **Baseline** | 4.57±0.80 | 4.60±0.73 | -0.03[0.46,0.39] | 0.04 | 0.872 |
|  | **Post-test** | 5.04±0.84 | 4.92±0.79 | 0.12[0.34,0.59] | 0.15 | 0.606 |
| **EO.RT.STND.CONT** | **Baseline** | 4.70±0.85 | 4.71±0.79 | -0.01[-0.46,0.44] | 0.01 | 0.961 |
|  | **Post-test** | 4.93±0.83 | 4.95±0.81 | -0.02[-0.46,0.44] | 0.02 | 0.940 |
| **EO.LT.STND.CONT** | **Baseline** | 4.32±0.83 | 4.33±0.75 | -0.01[0.45,0.43] | 0.02 | 0.955 |
|  | **Post-test** | 4.53±0.84 | 4.52±0.79 | 0.01[-0.43,0.45] | 0.01 | 0.973 |
| **TMF.RT.PRN.REST** | **Baseline** | 22.87±3.11 | 22.88±2.50 | -0.00[-1.64,1.63] | -0.001 | 0.998 |
|  | **Post-test** | 25.09±2.71 | 23.58±2.40 | 1.51[0.02,3.00] | 0.590 | 0.047* |
| **TMF.LT.PRN.REST** | **Baseline** | 22.75±2.92 | 22.65±2.37 | 0.10[-1.44,1.65] | 0.039 | 0.893 |
|  | **Post-test** | 24.74±2.91 | 23.20±2.31 | 1.53[0.00,3.07] | 0.584 | 0.049* |
| **TMF.RT.PRN.CONT** | **Baseline** | 25.5. ±2.83 | 25.44±2.20 | 0.05[-1.14,1.53] | 0.023 | 0.936 |
|  | **Post-test** | 27.94±2.94 | 26.06±2.07 | 1.88[0.40,3.36] | 0.742 | 0.014* |
| **TMF.LT.PRN.CONT** | **Baseline** | 25.21±2.87 | 24.99±1.95 | 0.21[-1.21,1.64] | 0.090 | 0.759 |
|  | **Post-test** | 28.21±2.79 | 25.60±1.98 | 2.61[1.19,4.02] | 1.071 | 0.001* |
| **TMF. RT.SIT.REST** | **Baseline** | 1.08±0.13 | 1.07±0.08 | 0.00[-0.05,0.07] | 0.083 | 0.775 |
|  | **Post-test** | 1.18±0.10 | 1.12±0.08 | 0.06[0.00,0.11] | 0.645 | 0.030* |
| **TMF. LT.SIT.REST** | **Baseline** | 1.05±0.11 | 1.05±0.11 | 0.00[-0.06,0.06] | 0.018 | 0.951 |
|  | **Post-test** | 1.15±0.11 | 1.08±0.11 | 0.07[0.00,0.13] | 0.612 | 0.039* |
| **TMF.RT.SIT.CONT** | **Baseline** | 1.06±0.10 | 1.06±0.07 | -0.00[-0.05,0.04] | -0.051 | 0.861 |
|  | **Post-test** | 1.16±0.09 | 1.10±0.08 | 0.06[0.00,0.11] | 0.686 | 0.022* |
| **TMF. LT.SIT.CONT** | **Baseline** | 1.04±0.11 | 1.03±0.09 | 0.00[-0.05,0.06] | 0.053 | 0.858 |
|  | **Post-test** | 1.15±0.10 | 1.08±0.10 | 0.06[0.00,0.12] | 0.648 | 0.030* |
| **TMF.RT.STND.REST** | **Baseline** | 1.09±0.15 | 1.09±0.11 | 0.00[-0.07,0.08] | 0.030 | 0.918 |
|  | **Post-test** | 1.19±0.10 | 1.13±0.10 | 0.06[0.00,0.12] | 0.603 | 0.042* |
| **TMF.LT. STND.REST** | **Baseline** | 1.13±0.13 | 1.14±0.16 | -0.00[-0.08,0.08] | -0.030 | 0.919 |
|  | **Post-test** | 1.26±0.13 | 1.17±0.15 | 0.08[0.00,0.18] | 0.618 | 0.039* |
| **TMF.RT.STND.CONT** | **Baseline** | 1.08±0.11 | 1.08±0.10 | -0.00[-0.06,0.06] | -0.016 | 0.954 |
|  | **Post-test** | 1.18±0.08 | 1.11±0.11 | 0.06[0.00,0.12] | 0.645 | 0.030* |
| **TMF.LT.STND.CONT** | **Baseline** | 1.12±0.13 | 1.12±0.13 | 0.00[-0.07,0.08] | 0.053 | 0.856 |
|  | **Post-test** | 1.23±0.10 | 1.15±0.13 | 0.07[0.00,0.14] | 0.662 | 0.027* |
| **CMF.RT.PRN.REST** | **Baseline** | 22.76±1.57 | 22.84±1.88 | -0.08[-1.09,0.92] | -0.048 | 0.868 |
|  | **Post-test** | 25.24±1.45 | 22.84±1.88 | 0.97[0.02,1.92] | 0.597 | 0.044* |
| **CMF.LT.PRN.REST** | **Baseline** | 23.42±2.08 | 23.24±1.26 | 0.18[-0.82,1.18] | 0.105 | 0.718 |
|  | **Post-test** | 25.75±2.24 | 24.63±1.31 | 1.12[0.05,2.19] | 0.609 | 0.040* |
| **CMF.RT.PRN.CONT** | **Baseline** | 25.49±1.46 | 25.26±1.70 | 0.23[-0.68,1.15] | 0.148 | 0.611 |
|  | **Post-test** | 28.33±1.44 | 26.77±2.30 | 1.55[0.44,2.66] | 0.816 | 0.007* |
| **CMF.LT.PRN.CONT** | **Baseline** | 26.30±2.12 | 25.92±1.40 | 0.37[-0.67,1.42] | 0.208 | 0.475 |
|  | **Post-test** | 29.18±2.59 | 27.82±1.74 | 1.36[0.07,2.64] | 0.614 | 0.039* |
| **CMF. RT.SIT.REST** | **Baseline** | 1.02±0.09 | 1.02±0.07 | 0.00[-0.04,0.05] | 0.028 | 0.925 |
|  | **Post-test** | 1.12±0.09 | 1.06±0.06 | 0.05[0.00,0.10] | 0.698 | 0.020* |
| **CMF. LT.SIT.REST** | **Baseline** | 0.98±0.08 | 0.98±0.08 | 0.00[-0.04,0.05] | 0.015 | 0.959 |
|  | **Post-test** | 1.08±0.08 | 1.02±0.07 | 0.05[0.01,0.10] | 0.713 | 0.017* |
| **CMF.RT.SIT.CONT** | **Baseline** | 1.01±0.09 | 1.01±0.07 | -0.00[-0.04,0.05] | -0.094 | 0.745 |
|  | **Post-test** | 1.12±0.09 | 1.05±0.07 | 0.06[0.01,0.11] | 0.764 | 0.011* |
| **CMF. LT.SIT.CONT** | **Baseline** | 0.96±0.07 | 0.96±0.05 | -0.00[-0.03,0.03] | -0.015 | 0.956 |
|  | **Post-test** | 1.08±0.08 | 1.02±0.07 | 0.05[0.00,0.09] | 0.657 | 0.028* |
| **CMF.RT.STND.REST** | **Baseline** | 1.05±0.10 | 1.05±0.07 | 0.00[-0.04,0.05] | 0.050 | 0.865 |
|  | **Post-test** | 1.15±0.08 | 1.09±0.08 | 0.05[0.00,0.11] | 0.692 | 0.020* |
| **CMF.LT. STND.REST** | **Baseline** | 1.00±0.11 | 1.01±0.06 | -0.00[-0.06,0.04] | -0.074 | 0.799 |
|  | **Post-test** | 1.10±0.10 | 1.04±0.07 | 0.05[0.00,0.11] | 0.610 | 0.040* |
| **CMF.RT.STND.CONT** | **Baseline** | 1.04±0.09 | 1.04±0.09 | -0.00[-0.05,0.05] | -0.045 | 0.879 |
|  | **Post-test** | 1.14±0.8 | 1.07±0.10 | 0.06[0.01,0.12] | 0.686 | 0.022* |
| **CMF.LT.STND.CONT** | **Baseline** | 0.99±0.11 | 0.99±0.06 | -0.00[0.05,0.05] | -0.024 | 0.935 |
|  | **Post-test** | 1.09±0.10 | 1.03±0.06 | 0.06[0.01,0.11] | 0.699 | 0.020* |

Significant at P ≤ 0.05

SE: stabilization exercise, PNE: pain neuroscience education, TA: Transverse Abdominis, IO: Internal Oblique, EO: External Oblique, TMF: thickness of Multifidus, LT: left, RT: right, CONT: contract, SUP: supine, PRN: prone, THICK: thickness, CMF: cross sectional area of Multifidus, SIT: sitting,
